# Evaluation of prognostic scores in patients with HCC undergoing first-line immunotherapy with atezolizumab and bevacizumab

**DOI:** 10.1016/j.jhepr.2024.101295

**Published:** 2024-12-05

**Authors:** Simon Johannes Gairing, Philipp Mildenberger, Jennifer Gile, Fabian Artusa, Bernhard Scheiner, Catherine Leyh, Sabine Lieb, Friedrich Sinner, Vincent Jörg, Thorben Fruendt, Vera Himmelsbach, Nada Abedin, Cennet Sahin, Katrin Böttcher, Jasmin Schuhbaur, Simon Labuhn, James Korolewicz, Claudia A.M. Fulgenzi, Antonio D'Alessio, Valentina Zanuso, Florian Hucke, Natascha Röhlen, Najib Ben Khaled, Eleonora Ramadori, Lukas Müller, Arndt Weinmann, Roman Kloeckner, Peter Robert Galle, Nguyen H. Tran, Sudhakar K. Venkatesh, Andreas Teufel, Matthias Ebert, Enrico N. De Toni, Dirk-Thomas Waldschmidt, Jens U. Marquardt, Dominik Bettinger, Markus Peck-Radosavljevic, Andreas Geier, Florian P. Reiter, Lorenza Rimassa, David J. Pinato, Christoph Roderburg, Thomas Ettrich, Michael Bitzer, Veit Scheble, Ursula Ehmer, Marie-Luise Berres, Fabian Finkelmeier, Maria Angeles Gonzalez-Carmona, Johann von Felden, Kornelius Schulze, Marino Venerito, Florian van Bömmel, Leonie S. Jochheim, Matthias Pinter, Raphael Mohr, Sumera I. Ilyas, Irene Schmidtmann, Friedrich Foerster

**Affiliations:** 1 Department of Internal Medicine I, University Medical Center of the Johannes Gutenberg University Mainz, Mainz, Germany; 2 Institute of Medical Biometry, Epidemiology and Informatics of the Johannes Gutenberg University Mainz, Mainz, Germany; 3 Department of Oncology, Mayo Clinic, Rochester, MN, USA; 4 Department of Hepatology and Gastroenterology, Charité–Universitätsmedizin Berlin, Campus Virchow-Klinikum and Campus Charité Mitte, Berlin, Germany; 5 Division of Gastroenterology & Hepatology, Department of Medicine III, Medical University of Vienna, Vienna, Austria; 6 Department of Gastroenterology and Hepatology, Essen University Hospital, Essen, Germany; 7 Department of Medicine II, Division of Hepatology, Leipzig University Medical Center, Leipzig, Germany; 8 Department of Gastroenterology, Hepatology and Infectious Diseases, Otto von Guericke University Hospital, Magdeburg, Germany; 9 I. Department of Internal Medicine, Gastroenterology & Hepatology, University Medical Center Hamburg-Eppendorf, Hamburg, Germany; 10 Medical Clinic 1, University Hospital, Goethe-University Frankfurt, Frankfurt am Main, Germany; 11 Medical Department III, University Hospital RWTH Aachen, Aachen, Germany; 12 Clinical Department for Internal Medicine II, Department of Clinical Medicine, TUM School of Medicine and Health, University Medical Center, Technical University of Munich, Germany; 13 Department of Internal Medicine I, University of Ulm, Ulm, Germany; 14 Clinic for Gastroenterology, Hepatology and Infectious Diseases, University Hospital Düsseldorf, Medical Faculty of Heinrich Heine University Düsseldorf, Düsseldorf, Germany; 15 Department of Surgery & Cancer, Faculty of Medicine, Imperial College London, Hammersmith Hospital, London, UK; 16 Department of Biomedical Sciences, Humanitas University, Pieve Emanuele (Milan), Italy; 17 Medical Oncology and Hematology Unit, Humanitas Cancer Center, IRCCS Humanitas Research Hospital, Rozzano (Milan), Italy; 18 Department of Internal Medicine and Gastroenterology (IMuG), Hepatology, Endocrinology, Rheumatology and Nephrology including Centralized Emergency Department (ZAE), Klinikum Klagenfurt am Worthersee, Klagenfurt, Kärnten, Austria; 19 Department of Medicine II, Medical Center University of Freiburg, Freiburg, Germany; 20 Department of Medicine II, University Hospital, Ludwig Maximilian University Munich, Munich, Germany; 21 Department of Gastroenterology and Hepatology, University of Cologne, Cologne, Germany; 22 Department of Diagnostic and Interventional Radiology, University Medical Center of the Johannes Gutenberg University Mainz, Mainz, Germany; 23 Institute of Interventional Radiology, University Hospital Schleswig-Holstein, Lübeck, Germany; 24 Abdominal Imaging Division, Department of Radiology, Mayo Clinic, Rochester, MN, USA; 25 Division of Hepatology, Division of Clinical Bioinformatics, Department of Medicine II, Medical Faculty Mannheim, Heidelberg University, Mannheim, Germany; 26 Clinical Cooperation Unit Healthy Metabolism Medical Faculty Mannheim, Heidelberg University, Mannheim, Germany; 27 Department of Medicine II, Medical Faculty Mannheim, Heidelberg University, Mannheim, Germany; 28 Department of Internal Medicine 1, University Hospital Schleswig-Holstein, Lübeck, Germany; 29 Division of Hepatology, Department of Medicine II, University Hospital Würzburg, Würzburg, Germany; 30 Division of Oncology, Department of Translational Medicine, University of Piemonte Orientale, Novara, Italy; 31 Department of Internal Medicine 1, Division for Gastroenterology, Hepatology, Infectiology, Gastrointestinal Oncology and Geriatrics, University Hospital of Tübingen, Tübingen, Germany; 32 Department I of Internal Medicine, University Hospital of Bonn, Bonn, Germany; 33 Division of Gastroenterology and Hepatology, Mayo Clinic Rochester, Rochester, MN, USA

**Keywords:** Hepatocellular carcinoma, Cirrhosis, Systemic therapy, Prediction model, Outcome, PD-L1, VEGF, Development, Validation, CABLE score, Immunotherapy, Immune checkpoint inhibitors

## Abstract

**Background & Aims:**

Immunotherapy with atezolizumab and bevacizumab (a + b) has improved the prognosis of patients with unresectable hepatocellular carcinoma (HCC). However, the outcome for individual patients is highly variable. This study aimed to (i) develop and validate a prognostic prediction model to estimate individual prognosis and (ii) compare it with established models.

**Methods:**

In this multicenter retrospective study, patients with HCC undergoing first-line immunotherapy with a + b from 24 centers (Europe, USA) were included. Statistical analysis and reporting followed the TRIPOD guidelines. The primary objective was overall survival (OS). A Cox model was developed and externally validated.

**Results:**

In total, 683 patients were included (training: 526, validation: 157). The C-reactive protein, albumin, bilirubin, lymphocytes, ECOG performance status, and extrahepatic spread (CABLE score) remained significantly associated with OS in Cox regression analysis. In the training set, the CABLE score had a higher discriminatory accuracy relative to ALBI, EZ-ALBI, mALBI, CRAFITY, PNI, NLR, PLR, and GPS (time-dependent AUC 0.79 and C-index 0.75 (95% CI 0.71–0.78) at 12 months). In the external validation set, the discriminatory performance of the CABLE score was comparable to ALBI, EZ-ALBI, and mALBI, but on average higher than PNI, CRAFITY, NLR, PLR, and GPS. In patients with Child-Pugh A, the CABLE score outperformed ALBI, EZ-ALBI, and mALBI in the first 9 months. We provide a web-based calculator for the CABLE score to allow estimation of individual prognosis for these patients (http://shiny.imbei.uni-mainz.de:3838/CABLE_Score/).

**Conclusions:**

The CABLE score shows good discriminatory performance in assessing the individual prognosis of patients undergoing first-line immunotherapy with a + b. Further validation studies are needed to investigate its performance compared with the ALBI score, in particular in subgroup analysis.

**Impact and implications:**

The CABLE score allows estimation of the prognosis of patients with unresectable hepatocellular carcinoma undergoing first-line immunotherapy with atezolizumab and bevacizumab at an individual level using our web-based calculator. This feature, as well as the evaluation of the score’s added benefit through an extensive comparison with other established scores, can inform clinicians on their significance and may guide clinical decision-making in the context of a malignant disease where the prognosis has become highly variable. Further large validation studies are needed to investigate the incremental value of the CABLE score compared with the ALBI score, in particular in subgroups such as patients designated as Child-Pugh A.

## Highlights

• The prognosis of patients with unresectable HCC receiving immunotherapy with atezolizumab and bevacizumab varies greatly.
• Several scores to predict the prognosis of patients have been established but their incremental value has so far been unknown.
• We developed and validated the CABLE score allowing to estimate the individual prognosis of patients using a web-based calculator.
• We found large differences in the predictive performance of the models with the CABLE score, ALBI, EZ-ALBI, and mALBI showing the highest accuracy.
• In patients with Child-Pugh A, the CABLE score outperformed ALBI, EZ-ALBI, and mALBI in the first 9 months.

## Graphical abstract

**Figure:**
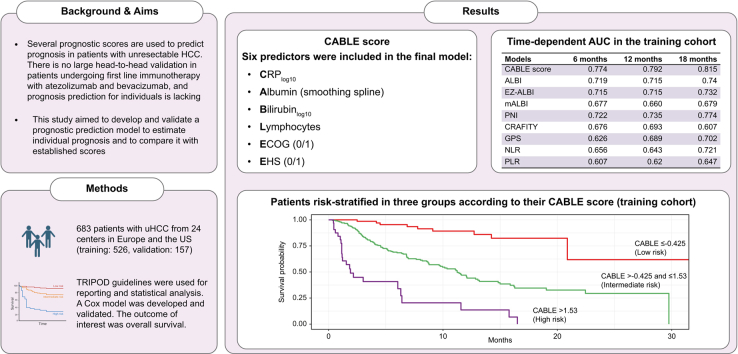

## Introduction

Liver cancer is the third leading cause of cancer-associated deaths worldwide and its incidence is predicted to continue to rise.^1^^,^^2^ In particular, unresectable hepatocellular carcinoma (uHCC) is associated with poor prognosis. Immunotherapy with atezolizumab (a; anti-programmed death-ligand 1 [PD-L1] antibody) and bevacizumab (b; anti-vascular endothelial growth factor [VEGF] antibody) has significantly improved prognosis of patients with unresectable HCC relative to sorafenib yielding a median overall survival (OS) of 19.2 months.[3], [4], [5] Thus, a + b is currently one of the recommended first-line (1L) systemic treatments for patients with uHCC.^6^^,^^7^

In addition to tumor-related characteristics, the prognosis of this population is highly dependent on several additional factors arising from the underlying chronic liver disease. In this context, systemic inflammation plays a key role by fueling the development of HCC irrespective of its underlying etiology.^8^ In addition, various biomarkers reflecting the stage of systemic inflammation including C-reactive protein (CRP), lymphocytes or the negative acute-phase protein albumin are associated with outcome in HCC.^8^

Currently, there are several prognostic scoring systems reflecting liver function, systemic inflammation or immunonutritive status.[9], [10], [11], [12] In the past, the Child-Pugh score has been used to assess liver function in patients with HCC. However, the albumin–bilirubin (ALBI) score which contains only two predictors (albumin and bilirubin) is increasingly replacing the Child-Pugh score because of its complete objectivity and at least similar discriminative performance in patients with HCC^9^^,^^13^^,^^14^ and is increasingly being used in the setting of clinical trials evaluating treatments for HCC.^9^^,^^15^^,^^16^ Correspondingly, *post hoc* analyses of phase 3 trials testing a + b,^17^ STRIDE,^18^ lenvatinib,^19^ cabozantinib,^20^ Click or tap here to enter text. and ramucirumab^21^ have demonstrated the prognostic value of the ALBI score (for a comprehensive review of the body of evidence for the role of ALBI in HCC refer to Demirtas *et al.*^22^). In addition, further modifications of the ALBI score such as easy-ALBI (EZ-ALBI)^23^ Click or tap here to enter text. and modified ALBI^24^^,^^25^ have been suggested to improve its prognostic ability and applicability. In the context of systemic treatment of uHCC with a + b, several other prognostic scores have been proposed or evaluated previously: (i) The CRAFITY (CRP and AFP in immunotherapy) score which combines CRP and alpha-fetoprotein (AFP).^10^ (ii) The Glasgow Prognostic Score (GPS) which combines albumin with CRP, and which was also shown to be associated with OS.^26^^,^^27^ (iii) The Prognostic Nutritional Index (PNI) which combines albumin and absolute lymphocyte counts which has been demonstrated to be an independent prognostic factor for OS and progression-free survival (PFS).[28], [29], [30] (iv) Scores reflecting selected cellular components of the peripheral blood and thereby mirroring the extent of systemic inflammation, namely, neutrophil-to-lymphocyte ratio (NLR) and platelet-to-lymphocyte ratio (PLR), which have been shown to be associated with OS and PFS.^11^^,^[31], [32], [33], [34], [35]

However, the incremental value of these prediction models is unknown, and it is unclear which model serves the purpose of prognosis estimation best. To address this knowledge gap, this study aimed to evaluate the prognostic value of all these candidate predictors and to integrate them into one prognostic prediction model that allows accurate estimation of the individual prognosis of patients with HCC undergoing 1L immunotherapy with a + b.

## Patients and methods

This study and manuscript are based on the *Transparent Reporting of a multivariable prediction model for Individual Prognosis Or Diagnosis* (TRIPOD) statement.^36^^,^^37^ The methodological approach was based on that of Åberg *et al.*^38^ (CLivD score).

### Study design and patients

This retrospective multicenter study included patients from a total of 24 centers in Germany (DE), Austria (AT), Italy (IT), the UK, and the USA. The data set was split into a training set (center 1–12) and a validation set (center 13–24) based on the time of their data reporting. Immunotherapy with a + b was initiated between March 2018 and November 2022. Data were received until December 2022. Further details including the outcome of interest, candidate predictors, response assessment, sample size, and calculation of scores are summarized in the Supplementary methods.

### Ethics

This study was approved by the ethics committee of the Medical Association of Rhineland Palatinate, Mainz, Germany (permit number 2022-16779) and conducted according to the ethical guidelines of the 1975 Declaration of Helsinki (6th revision, 2008).

### Statistical analysis

Details on descriptive statistics, modification of candidate predictors, and handling of missing data are given in the Supplementary methods.

#### Model building

The model development consisted of two parts. In the first part, a model was fitted on the imputed data with variables having ≤10% missingness. In the second part, we examined whether the variables with >10% missingness could improve prediction in the complete cases subset (see section *Missing data*).

In the first part, a preselection of variables was made using univariable Cox proportional hazard models with center as random effect to account for different case mixes across participating centers. Continuous variables were fitted with cubic regression splines to account for potential non-linear effects. Variables with *p* <0.1 were considered for the multivariable model. We then imputed 10 sets with predictive mean matching, using the R-package mice and fitted a model with only continuous terms on each imputation set. Shrinkage smoothers were used for variable selection as described in Marra and Wood.^39^ Knots were placed explicitly to allow for later pooling of the models. We considered three variable sets of continuous variables as candidates with variable inclusion frequency 10%, 50%, and 100%, respectively. For categorical variables, we used forward selection on each imputation, which similarly resulted in three candidate sets of categorical variables. We combined continuous and categorical variable sets to get 3 × 3 = 9 candidate models. Those candidate models were compared with repeated cross validation with 10 repetitions and five folds. This led to 50 imputations because the ‘hold-out-fold’ was ignored in the imputation procedure to prevent contamination. The model with the best time-dependent AUC was then selected.

In the second part, the variables with missingness of >10% were then added to the resulting model from the first part. A backward–forward elimination on the subset of complete cases resulted in the final model.

#### Model performance measures

##### Discrimination

The model performance in terms of discrimination was analyzed by the time-dependent AUC using the R-package timeROC and Uno’s C with bootstrapped confidence intervals.^40^^,^^41^

##### Calibration

To analyze the calibration of the prediction model, patients in the training and validation set were divided into quintiles according to their prognosis in the CABLE score. Subsequently, the agreement between the observed and predicted survival was visually compared using the Kaplan–Meier curve within each quintile.^42^

#### Risk groups

Risk groups were identified by dividing the patients into three groups according to their prognostic score (low risk: lowest 25%, intermediate risk: 25–90%, high risk: highest 10%).

#### External validation

**Fig. 1:**
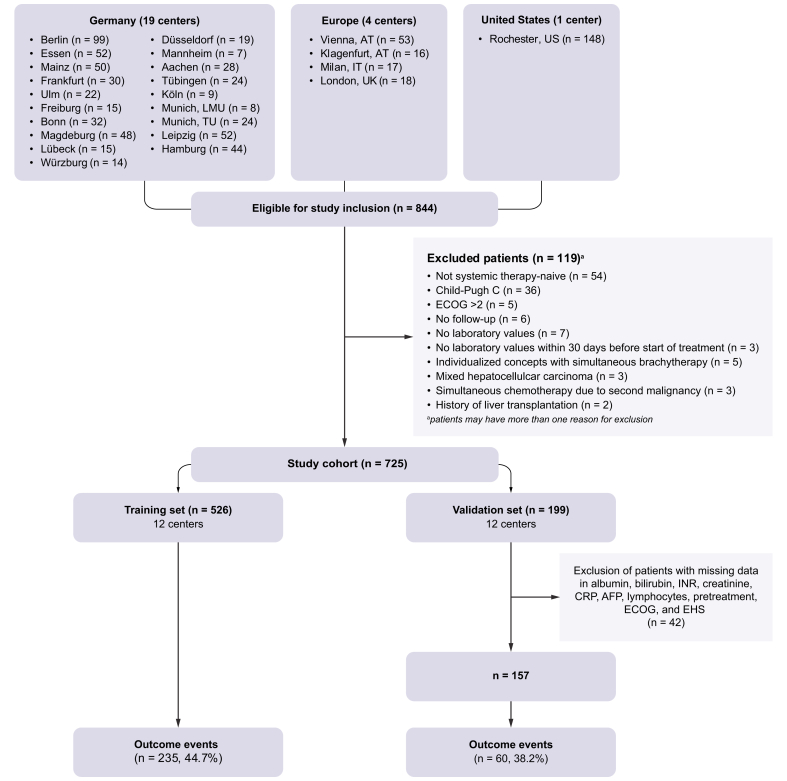
Flow chart of patients eligible for study inclusion. AT, Austria; ECOG, Eastern Cooperative Oncology Group; GB, Great Britain; IT, Italy; LMU, Ludwig Maximilian University of Munich; TU, Technical University of Munich; US, United States.

To externally validate the newly developed model, data from centers 13–24 were used. Here, only patients with complete data on the variables included in the models and the established scores were used (n = 157, outcome events = 60, Fig. 1). Again, discrimination was analyzed by comparing the time-dependent AUC and Uno’s C.

## Results

### Study cohort

The training cohort included 526 patients, the external validation cohort 157 patients. Fig. 1 displays the composition of the training and validation cohort and the number of outcome events, respectively. Before data analysis, we decided to use centers 1–12 as the training set, and centers 13–24 as the validation set. Table 1 summarizes the baseline characteristics of the study participants for both cohorts including the number of missing values for each candidate predictor. Compared with the training set, the validation cohort had significantly lower Eastern Cooperative Oncology Group (ECOG) performance stages, a lower frequency of pretreatment, different etiology composition (more alcohol-associated, less viral), lower international normalized ratio (INR) levels, and no brachytherapy. No outcome event data were missing. The median follow-up time was 12.6 months (95% CI: 11.7–14.0) for the training set, and 11.9 months (95% CI: 10.9–14.8) for the validation set (reverse Kaplan–Meier method). In total, two patients were censored because of liver transplantation during follow-up. The reasons for discontinuation of a + b are detailed in Table S2.

**Table 1:** Demographics and baseline characteristics of the study cohort.

	Training set (n = 526)	Validation set (n = 157)	*p* values
Age (years)			
Median (Min, Max)	67.0 (25.0, 89.5)	69.0 (29.0, 85.0)	0.1
Sex			
Male	427 (81.2%)	127 (80.9%)	1
Female	99 (18.8%)	30 (19.1%)	
ECOG			
0	239 (45.4%)	92 (58.6%)	0.009
1	251 (47.7%)	60 (38.2%)	
2	36 (6.8%)	5 (3.2%)	
Cirrhosis			
No	133 (25.3%)	41 (26.3%)	0.9
Yes	393 (74.7%)	115 (73.7%)	
Missing	0 (0%)	1 (0.6%)	
Etiology			
Unknown/no liver disease	91 (17.3%)	34 (21.7%)	<0.001
HBV	55 (10.5%)	7 (4.5%)	
HCV	93 (17.7%)	22 (14.0%)	
Harmful alcohol use	128 (24.3%)	62 (39.5%)	
MASLD/MASH	89 (16.9%)	15 (9.6%)	
Mixed (viral and non-viral)	34 (6.5%)	6 (3.8%)	
Mixed (only non-viral)	17 (3.2%)	3 (1.9%)	
Mixed (only viral)	1 (0.2%)	2 (1.3%)	
Other	18 (3.4%)	6 (3.8%)	
BCLC			
A	8 (1.5%)	0 (0%)	0.2
B	133 (25.3%)	47 (29.9%)	
C	385 (73.2%)	110 (70.1%)	
Pretreatment			
No	231 (43.9%)	87 (55.4%)	0.01
Yes	295 (56.1%)	70 (44.6%)	
History of surgery			
No	419 (79.7%)	131 (83.4%)	0.3
Yes	107 (20.3%)	26 (16.6%)	
History of ablation			
No	469 (89.2%)	141 (89.8%)	0.9
Yes	57 (10.8%)	16 (10.2%)	
History of TACE/TAE			
No	358 (68.1%)	115 (73.2%)	0.3
yes	168 (31.9%)	42 (26.8%)	
History of SIRT/TARE			
No	475 (90.3%)	143 (91.1%)	0.9
Yes	51 (9.7%)	14 (8.9%)	
History of radiation/SBRT			
No	517 (98.3%)	157 (100%)	0.2
Yes	9 (1.7%)	0 (0%)	
History of brachytherapy			
No	502 (95.4%)	157 (100%)	0.01
Yes	24 (4.6%)	0 (0%)	
Macrovascular invasion (MVI)			
No	295 (56.1%)	97 (61.8%)	0.2
Yes	231 (43.9%)	60 (38.2%)	
Extrahepatic spread (EHS)			
No	311 (59.1%)	105 (66.9%)	0.1
Yes	215 (40.9%)	52 (33.1%)	
Child-Pugh			
A	384 (73.4%)	114 (72.6%)	0.9
B	139 (26.6%)	43 (27.4%)	
Missing	3 (0.6%)	0 (0%)	
ALBI score			
Median (Min, Max)	-2.42 (-3.70, -0.733)	-2.40 (-3.46, -0.677)	0.7
Missing	21 (4.0%)	0 (0%)	
ALBI grade			
1	194 (38.4%)	65 (41.4%)	0.08
2	287 (56.8%)	78 (49.7%)	
3	24 (4.8%)	14 (8.9%)	
Missing	21 (4.0%)	0 (0%)	
mALBI grade			
1	194 (38.4%)	65 (41.4%)	0.2
2a	102 (20.2%)	27 (17.2%)	
2b	185 (36.6%)	51 (32.5%)	
3	24 (4.8%)	14 (8.9%)	
Missing	21 (4.0%)	0 (0%)	
EZ-ALBI			
(Min, Max)	-32.9 (-45.2, -14.6)	-32.5 (-42.7, -14.6)	0.4
Missing	21 (4.0%)	0 (0%)	
CRAFITY score			
Low	104 (29.7%)	51 (32.5%)	0.7
Intermediate	151 (43.1%)	62 (39.5%)	
High	95 (27.1%)	44 (28.0%)	
Missing	176 (33.5%)	0 (0%)	
GPS			
GPS 0	130 (37.4%)	64 (40.8%)	0.8
GPS 1	133 (38.2%)	56 (35.7%)	
GPS 2	85 (24.4%)	37 (23.6%)	
Missing	178 (33.8%)	0 (0%)	
PNI			
Median (Min, Max)	43.2 (23.0, 63.1)	42.9 (20.8, 58.0)	0.6
Missing	74 (14.1%)	0 (0%)	
NLR			
Median (min, max)	3.62 (0.337, 117)	3.87 (0.834, 21.8)	0.7
Missing	62 (11.8%)	0 (0%)	
PLR			
Median (min, max)	151 (33.3, 3300)	152 (25.8, 852)	0.5
Missing	63 (12.0%)	0 (0%)	
Bilirubin (mg/dl)			
Median (min, max)	0.800 (0.160, 12.2)	0.748 (0.170, 6.31)	0.4
Missing	1 (0.2%)	0 (0%)	
Albumin (g/dl)			
Median (min, max)	3.80 (1.70, 5.16)	3.70 (1.73, 4.80)	0.4
Missing	21 (4.0%)	0 (0%)	
INR			
Median (min, max)	1.15 (0.890, 3.90)	1.10 (0.900, 4.30)	0.01
Missing	6 (1.1%)	0 (0%)	
Creatinine (mg/dl)			
Median (min, max)	0.870 (0.386, 8.00)	0.890 (0.380, 5.40)	0.4
Missing	2 (0.4%)	0 (0%)	
CRP (mg/l)			
Median (min, max)	10.2 (0.200, 299)	10.0 (0.100, 173)	0.6
Missing	172 (32.7%)	0 (0%)	
AFP (ng/ml)			
Median (min, max)	56.1 (1.00, 2,070,000)	44.1 (1.00, 91,000)	0.7
Missing	11 (2.1%)	0 (0%)	
Platelets per nl			
Median (min, max)	164 (29.0, 760)	170 (35.0, 483)	0.5
Missing	3 (0.6%)	0 (0%)	
Neutrophils per nl			
Median (min, max)	4.10 (0.550, 21.0)	4.10 (0.730, 55.7)	1
Missing	47 (8.9%)	0 (0%)	
Lymphocytes per nl			
Median (min, max)	1.09 (0.0700, 3.59)	1.13 (0.210, 3.26)	1
Missing	62 (11.8%)	0 (0%)	
Monocytes per nl			
Median (min, max)	0.600 (0.0490, 2.29)	0.570 (0.170, 2.10)	0.6
Missing	63 (12.0%)	0 (0%)	

Continuous data are given as median and range (min, max), categorial data as frequency with percentage. Percentages refer to patients with available data. A Χ^2^ test was applied for categorial data, a Mann–Whitney *U* test or Student’s *t* test for continuous data (depending on data distribution). Patients may have received more than one category of pretreatment. AFP, alpha-fetoprotein; ALBI, albumin–bilirubin; BCLC, Barcelona Clinic Liver Cancer; CRAFITY, CRP, and AFP in immunotherapy; CRP, C-reactive protein; ECOG, Eastern Cooperative Oncology Group; EZ-ALBI, easy-ALBI; GPS, Glasgow Prognostic Score; HCC, hepatocellular carcinoma; INR, international normalized ratio; MASH, metabolic dysfunction-associated steatohepatitis; mALBI, modified ALBI; MASLD, metabolic dysfunction-associated steatotic liver disease; NLR, neutrophil-to-lymphocyte ratio, PLR, platelet-to-lymphocyte ratio; PNI, prognostic nutritional index; SBRT, stereotactic body radiation therapy; SIRT, selective internal radiation therapy; TACE, transarterial chemoembolization; TAE, transarterial embolization; TARE, transarterial radioembolization.

### Overall survival and tumor response

Median OS was 13.7 months (95% CI: 12.1–15.8) for the training cohort, and 16.0 months (95% CI: 13.5, not reached) for the validation cohort. In the subgroup of patients with Child-Pugh A and with an ECOG of 0–1, median OS was 20.9 months (95% CI: 15.7, not reached) in the training cohort and 19.0 months (95% CI: 14.9, not reached) in the validation cohort. In total, 343 patients in the training cohort (65.2%), and 142 patients (90.4%) in the validation cohort had available follow-up imaging data. The overall response rate (ORR) was 31.8% in the training, and 31.0% in the validation cohort. The disease control rate (DCR) was 71.7% in the training, and 69.7% in the validation cohort. Detailed response data are given in Table S3.

### Model building

The stepwise model building process is described in detail in the Patients and methods section. First, candidate predictors were preselected via univariable models (Table S4). In the imputed training set (n = 526), seven predictors including albumin (smoothing spline), creatinine_log10_ (smoothing spline), bilirubin_log10_, INR_log10_, ECOG (0/1/2), extrahepatic spread (EHS; no/yes), and pretreatment (no/yes) remained in the model after the variable selection procedure (Model with imputed data: Model_i_) Fig. S1A shows the smoothing splines of albumin and creatinine_log10_. Table S5 displays the adjusted log(HR) for Model_i_.

Next, the training set was trimmed to complete cases for the variables included in the Model_i_ and CRP, lymphocytes, and monocytes (n = 320, outcome events: 136). Baseline characteristics for this data set are given in Table S6. Here, six predictors remained in the model including albumin (smoothing spline), bilirubin_log10_, lymphocytes, CRP_log10_, ECOG (0/1 [whereby the value 1 includes ECOG 1 and 2]), and EHS (no/yes). This score was named CABLE score (CRP, Albumin, Bilirubin, Lymphocytes, ECOG, and EHS). Fig. S1B displays the non-linear predictor-outcome relation of albumin in the CABLE score. Table 2 displays the adjusted log(HR) for the CABLE score. Table S7 shows the variables included in the CABLE score compared with other prediction models.

**Table 2:** Adjusted multivariable Cox model of the CABLE score.

Variables	Beta∗	95% CI	*p* values
CRP_log10_	0.55	0.20–0.90	0.002
s(Albumin)			<0.001
Bilirubin_log10_	1.1	0.49–1.7	<0.001
Lymphocytes	-0.61	-0.98 to -0.25	<0.001
ECOG			
0	—	—	
1	0.60	0.21–1.0	0.003
EHS			
No	—	—	
Yes	0.40	0.03–0.77	0.033

Adjusted multivariable Cox model.

CRP, C-reactive protein; ECOG, Eastern Cooperative Oncology Group; EHS, extrahepatic spread; s, smoothing spline.

∗ log(HR).

### Internal validation

**Fig. 2:**
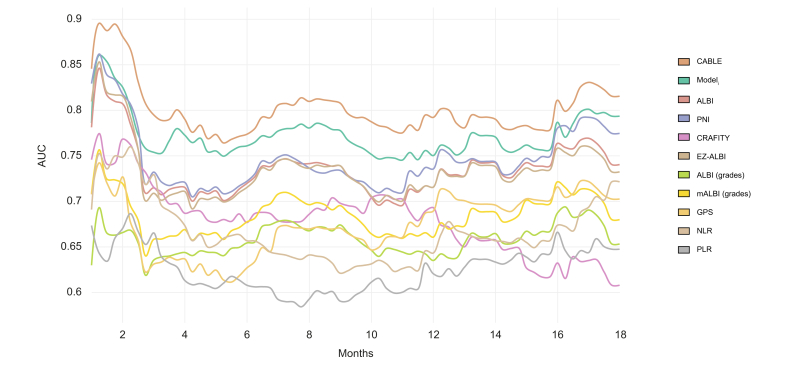
Time-dependent AUC of the CABLE score and Model_i_ in comparison with established prediction models in the training set. ALBI, albumin–bilirubin; CABLE, CRP, albumin, bilirubin, lymphocytes, ECOG, and EHS; CRAFITY, CRP and AFP in immunotherapy; EZ-ALBI, easy-ALBI; GPS, Glasgow Prognostic Score; mALBI, modified ALBI; Model_i_, Cox PH model derived from the imputed training set; NLR, neutrophil-to-lymphocyte ratio; PLR, platelet-to-lymphocyte ratio; PNI, Prognostic Nutritional Index.

For the internal and external validation, only individuals with complete data for the tested predictors were included (complete-case analysis). Fig. 2 displays the time-dependent AUC of the CABLE score compared to Model_i_, ALBI (continuous and graded), EZ-ALBI, mALBI, CRAFITY, PNI, NLR, PLR, and GPS. The CABLE score continuously had the highest AUC values (0.77–0.82). Model_i_ yielded the second highest AUC values (0.75–0.79). PNI, ALBI, and EZ-ALBI had AUC values >0.7. CRAFITY, mALBI, ALBI graded, and PLR had AUC values <0.7. Table S8 shows the AUC for all scores at 6, 12, and 18 months. Table 3 shows the respective Uno’s C indices for 6, 12, and 18 months. Here, the CABLE score showed the highest C-index at all time points. Additionally, only the CABLE score and Model_i_ yielded values >0.7. PNI, ALBI, and EZ-ALBI had C indices between 0.68 and 0.69. All other scores had C indices between 0.59 and 0.66.

**Table 3:** Uno’s C indices with bootstrapped confidence intervals for the training set.

Model	6 months	12 months	18 months
CABLE score	0.748 (0.692–0.804)	0.745 (0.709–0.781)	0.743 (0.707–0.78)
Model_i_	0.731 (0.681–0.782)	0.714 (0.666–0.762)	0.717 (0.674–0.76)
ALBI	0.692 (0.628–0.756)	0.681 (0.636–0.725)	0.689 (0.639–0.739)
ALBI grade	0.626 (0.583–0.67)	0.62 (0.582–0.659)	0.627 (0.588–0.665)
mALBI grade	0.65 (0.597–0.703)	0.638 (0.585–0.692)	0.65 (0.602–0.698)
EZ-ALBI	0.686 (0.634–0.739)	0.677 (0.635–0.719)	0.684 (0.64–0.728)
PNI	0.693 (0.636–0.751)	0.689 (0.636–0.741)	0.692 (0.646–0.739)
CRAFITY	0.662 (0.607–0.717)	0.66 (0.611–0.71)	0.633 (0.588–0.678)
GPS	0.607 (0.553–0.661)	0.627 (0.584–0.671)	0.63 (0.591–0.668)
NLR	0.643 (0.575–0.71)	0.62 (0.57–0.67)	0.623 (0.568–0.678)
PLR	0.594 (0.531–0.656)	0.589 (0.532–0.647)	0.588 (0.545–0.63)

ALBI, albumin–bilirubin; CABLE, CRP, albumin, bilirubin, lymphocytes, ECOG, and EHS; CRAFITY, CRP and AFP in immunotherapy; EZ-ALBI, easy-ALBI; GPS, Glasgow prognostic score; mALBI, modified ALBI; Model_i_, Cox PH (proportional hazards) model derived from the imputed training set; NLR, neutrophil-to-lymphocyte ratio; PLR, platelet-to-lymphocyte ratio; PNI, prognostic nutritional index.

### External validation

**Fig. 3:**
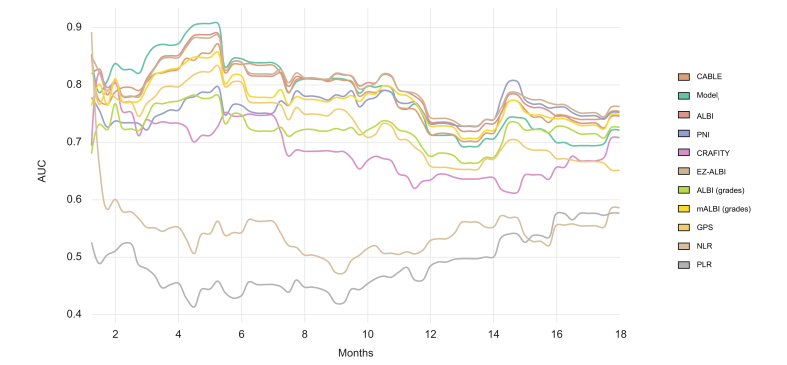
Time-dependent AUC of the CABLE score and Model_i_ compared to other prediction models in the validation cohort. ALBI, albumin–bilirubin; CABLE, CRP, albumin, bilirubin, lymphocytes, ECOG, and EHS; CRAFITY, CRP and AFP in immunotherapy; EZ-ALBI, easy-ALBI; GPS, Glasgow Prognostic Score; mALBI, modified ALBI; Model_i_, Cox PH model derived from the imputed training set; NLR, neutrophil-to-lymphocyte ratio; PLR, platelet-to-lymphocyte ratio. PNI, Prognostic Nutritional Index.

Fig. 3 displays the time-dependent AUC of the CABLE score compared to Model_i_, ALBI (continuous and graded), EZ-ALBI, mALBI, CRAFITY, PNI, NLR, PLR, and GPS in the validation cohort. The AUCs for 6, 12, and 18 months are displayed in Table S9. Compared with the training set, most models showed a higher time-dependent AUC on average at 6 months. At 6 months, the CABLE score, Model_i_, ALBI, and EZ-ALBI had the highest AUC values (AUC 0.84–0.85). mALBI and GPS had AUC values >0.8, while PNI, ALBI graded, and CRAFITY showed AUC values between 0.75 and 0.76. NLR and PLR had the lowest performance with AUC values <0.6. At 12 months, ALBI, EZ-ALBI, PNI, and mALBI had AUC values between 0.73 and 0.74, the CABLE score and Model_i_ values of 0.71. ALBI graded, GPS, CRAFITY, NLR, and PLR had values <0.7. At 18 months, the CABLE score, ALBI, EZ-ALBI, mALBI, and PNI had the highest AUC values (0.75–0.76), while ALBI graded, Model_i_, and CRAFITY had AUC values between 0.71–0.73. GPS, NLR, and PLR had values <0.7. Uno’s C indices for 6, 12, and 18 months are given in Table S10. The CABLE score, Model_i_, ALBI, EZ-ALBI, and mALBI had C indices >0.7 at all time points.

### Risk groups

**Fig. 4:**
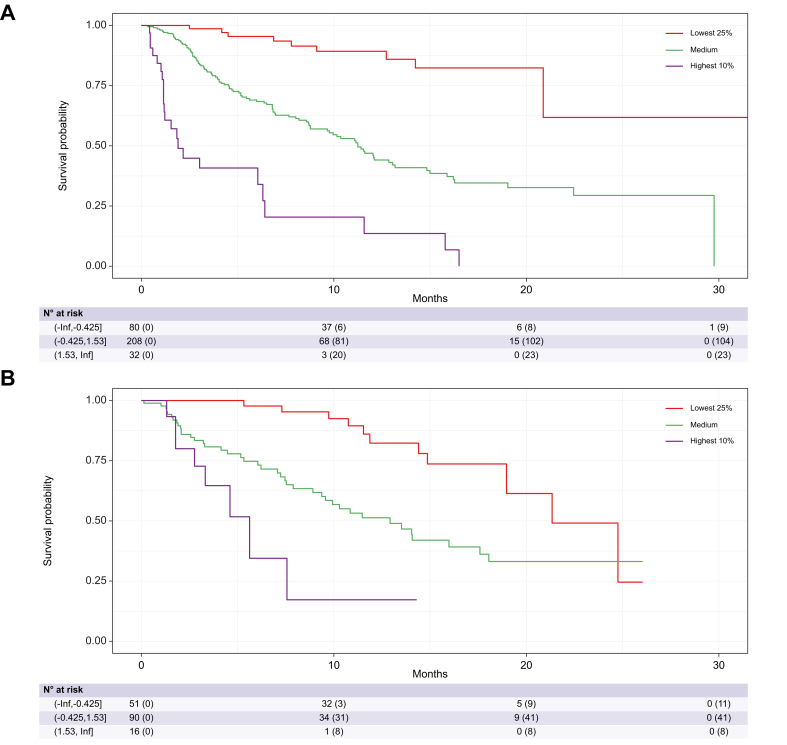
Kaplan–Meier curves of patients risk-stratified into three groups according to their CABLE score. In the (A) training and (B) validation set. The lower the CABLE score, the higher the survival probability. CABLE, CRP, albumin, bilirubin, lymphocytes, ECOG, and EHS.

For the CABLE score, risk groups were created by stratifying the training and validation set according to their prognostic score into three groups (low risk: lowest 25% (≤0.425), intermediate risk: 25–90% (>-0.425 and ≤1.53), high risk: highest 10% (>1.53)). Kaplan–Meier curves for the CABLE score for the training and validation set are shown in Fig. 4. The groups identified patients with clearly different prognosis yielding a median overall survival of *not reached* (95% CI: 20.88, *not reached*; low risk), 11.24 months (95% CI 9.67–14.83; intermediate risk), and 1.91 months (95% CI 1.18–11.57; high risk) in the training set, and 21.34 months (95% CI 18.97, *not reached*; low risk), 12.92 months (95% CI 9.37;18.05; intermediate risk), and 5.62 months (95% CI 3.32, NA; high risk) in the validation set.

### Calibration

Calibration plots for the CABLE score are shown in Fig. S2. The CABLE score showed a very good agreement in Q1–Q5 in the training set. In the validation set, the agreement in Q5 and Q4 was high, medium in Q2 and Q3, and low in Q1.

### Subgroup analysis

Next, we analyzed the performance of the CABLE score in relevant subgroups in the validation cohort (Table S11). For this purpose, we built univariable Cox regression models for each subgroup, using the score as the sole predictor. A higher HR indicates better relative performance. Here, the CABLE score performed well in all subgroups with the exception of the group of patients with Child-Pugh B.

Consequently, we analyzed the time-dependent AUC in the subgroup of patients with Child-Pugh A. In the training cohort, the CABLE score had the highest AUC over the entire observation period (Fig. S3). Corresponding Uno’s C indices are shown in Table S12. In the validation cohort, the CABLE score mostly showed higher AUCs than the ALBI score during the first 9 months after the start of a + b (Fig. S4). From 9 months onwards, CABLE and ALBI had almost the same AUC values. Uno’s C indices for 3, 6, 12, and 18 months for the CABLE score were 0.84, 0.74, 0.67, and 0.67 compared with 0.80, 0.66, 0.67, and 0.68 for the ALBI score (Table 4). EZ-ALBI and modified ALBI (mALBI) showed similar results as the ALBI score. PNI had similar results as the ALBI score in the first 5 months but showed better AUC values in the later observation period and even higher AUC values than the CABLE score from around month 8. CRAFITY showed similar results compared to the CABLE score in the first 5 months, showed a better AUC after 6 months, but had continuously lower AUC values from month 8 onwards. GPS, NLR, and PLR had continuously lower AUC values than the CABLE score (Table 4).

**Table 4:** Uno’s C indices for the validation set in the subgroup of patients with Child-Pugh A with bootstrapped confidence intervals.

Model	3 months	6 months	12 months	18 months
CABLE score	0.839 (0.673–1.004)	0.739 (0.571–0.908)	0.668 (0.566–0.77)	0.671 (0.575–0.767)
Model_i_	0.86 (0.618–1.103)	0.667 (0.406–0.928)	0.641 (0.524–0.758)	0.643 (0.528–0.757)
ALBI	0.803 (0.61–0.995)	0.664 (0.457–0.871)	0.667 (0.547–0.787)	0.676 (0.579–0.773)
EZ-ALBI	0.79 (0.612–0.969)	0.669 (0.459–0.879)	0.675 (0.567–0.783)	0.684 (0.587–0.781)
ALBI grade	0.724 (0.563–0.886)	0.63 (0.45–0.811)	0.615 (0.519–0.711)	0.632 (0.544–0.72)
mALBI grade	0.779 (0.592–0.966)	0.679 (0.489–0.868)	0.656 (0.557–0.754)	0.66 (0.568–0.753)
PNI	0.801 (0.63–0.972)	0.69 (0.501–0.879)	0.709 (0.608–0.81)	0.714 (0.623–0.805)
CRAFITY	0.836 (0.59–1.081)	0.859 (0.713–1.006)	0.613 (0.474–0.751)	0.618 (0.496–0.74)
GPS	0.78 (0.525–1.035)	0.725 (0.565–0.885)	0.626 (0.542–0.709)	0.611 (0.548–0.674)
NLR	0.627 (0.342–0.912)	0.578 (0.383–0.774)	0.564 (0.383–0.745)	0.565 (0.384–0.747)
PLR	0.454 (0.276–0.632)	0.524 (0.369–0.679)	0.475 (0.375–0.575)	0.451 (0.349–0.554)

ALBI, albumin–bilirubin; CABLE, CRP, albumin, bilirubin, lymphocytes, ECOG, and EHS; CRAFITY, CRP and AFP in immunotherapy; EZ-ALBI, easy-ALBI; GPS, Glasgow Prognostic Score; mALBI, modified ALBI; Model_i_, Cox PH model derived from the imputed training set; NLR, neutrophil-to-lymphocyte ratio; PLR, platelet-to-lymphocyte ratio; PNI, prognostic nutritional index.

### Prediction of ORR and DCR

Next, we tested whether the CABLE score and the other models tested are able to predict response to treatment with a + b. Table S13 shows the AUC of all models in the validation data set. In summary, all models had AUCs <0.7 and thus were not useful to predict either ORR or DCR. Table S14 shows the association of variables with ORR in terms of the explained deviance. Here, ECOG performance status, MVI, platelets, and neutrophils had a significant association with ORR. Etiology was not associated with ORR. In addition, we tried to fit new models aiming to predict response to a + b (data not shown), but given their very poor predictive performance, we did not validate these models.

### Online calculator

To make the CABLE score available and usable as a tool for prognosis assessment of patients with uHCC receiving a + b, we have developed an online calculator (http://shiny.imbei.uni-mainz.de:3838/CABLE_Score/). Here, the CABLE score is calculated and the survival probability at 6, 12, and 18 months is given. In addition, the R model for calculating the CABLE score is available for download from the app. The risk formula is not provided in the manuscript as it is not possible to include the non-linear terms here.

## Discussion

In this large multicenter study, we evaluated and compared the prognostic value of several clinical variables and established scores in patients with uHCC undergoing 1L immunotherapy with a + b. We developed and validated the CABLE score that allows prediction of the individual prognosis of these patients using the provided web-calculator. In the training set, the CABLE score outperformed all other scores. In the external validation set, the CABLE score had superior discriminatory performance compared with PNI, CRAFITY, NLR, PLR, and GPS and similar performance to ALBI, EZ-ALBI, and mALBI.

Although the CABLE score did not outperform ALBI, EZ-ALBI, and mALBI in the validation cohort, we believe that this study provides valuable insights for the following reasons. First, to our knowledge, we have performed the first statistically sound and thorough head-to-head validation of the currently used scores in a large, multicenter setting of a homogenous cohort including only patients with uHCC undergoing 1L immunotherapy with a + b. Here, we found large differences in the predictive performance of currently used scores, which is of great interest for clinicians using these scores in daily clinical practice. Second, all of the extracted patient- and tumor-related characteristics and laboratory values have previously been associated with outcome of patients with HCC and are part of several scoring systems. However, the incremental value of the variables used in these prediction models in terms of improving the predictive accuracy was unknown and the rationale for this study. Our study highlights the fact that adding variables which are highly associated with the outcome to a prediction model does not necessarily enhance its predictive performance. One explanation could be that our validation data set was too small or biased (see below). Another, and in our opinion more likely, explanation could be, that some of the information of the added variables is already captured to some extent by albumin and bilirubin (*e.g.* albumin is a negative acute-phase protein and could reflect changes in CRP levels). Consequently, other variables need to be investigated to improve the predictive accuracy of these models that capture completely independent information about patient prognosis (*e.g.* molecular markers or frailty). Third, in subgroup analyses including only patients with Child-Pugh A, the CABLE score outperformed ALBI, EZ-ALBI, and mALBI in the first 9 months. This could be because liver function reflected by albumin and bilirubin may be less relevant in patients with Child-Pugh A and that other variables such as CRP or lymphocytes may have a greater impact on the outcome in this subgroup. Fourth, we have launched a web-calculator which allows to estimate prognosis of patients on an individual level attributing them a survival probability value at 6, 12, and 18 months.

The CABLE score includes six variables which have exhibited the highest predictive performance in this cohort and are routinely measured at the start of therapy with a + b. It combines clinical information about systemic inflammation, liver insufficiency, tumor distribution, nutritional status, and the general condition of patients with HCC. Albumin is part of ALBI, EZ-ALBI, mALBI, PNI, and GPS and is a well-known highly prognostic parameter in patients with cirrhosis.^9^^,^[23], [24], [25], [26]^,^^28^^,^^29^ Bilirubin is included in ALBI, EZ-ALBI, and mALBI, reflecting the liver parenchymal damage.^9^^,^[23], [24], [25] CRP is incorporated in the CRAFITY score and GPS, and reflects the inflammatory state, where higher values are associated with worse outcome.^10^^,^^26^ Lymphocytes are part of PNI, NLR, and PLR.^11^^,^^28^^,^^29^^,^[31], [32], [33], [34], [35] Here, lower values at treatment start are associated with shorter survival. EHS reflects the systemic distribution of HCC, and, if positive, is frequently associated with worse prognosis. ECOG is the only not completely objective parameter included in the CABLE score. However, the prognostic value of the general condition of patients in oncology is well established.^43^ In addition, the CABLE score discriminates only ECOG 0 *vs.* ECOG 1/2, which is comparatively clear in most patients in our opinion.

Strengths of this work are the large multinational and homogenous cohort, yielding an appropriate proportion of outcome events for the development of a new prediction model. In addition, the TRIPOD-driven statistically elaborate development procedure and the external validation increase the robustness of our study. Furthermore, the CABLE score only includes broadly available and cost-effective predictors. However, this study has several limitations that have to be acknowledged. First, the retrospective nature of the study. Second, we integrated patients with missing data in the development cohort. However, the TRIPOD statement clearly advocates including patients with missing data by using multiple imputation rather than conducting a complete-case analysis.^36^^,^^37^ Third, some patients have received atezolizumab monotherapy intermittently for example because of an increased risk of bleeding. Fourth, the cohort was derived only from Western countries, which is reflected in the distribution of etiologies. Thus, the score needs to be validated in other countries not included in the current dataset. Fifth, while we could compare the CABLE score to various established scores in our study, we were not able to include several other previously published prognostic scores because of missing parameters in our data set (such as CAR (CRAFITY score and AFP-Response) classification,^44^ HCC-GRIm (hepatocellular carcinoma modified Gustave Roussy Immune) score,^45^ and ABE (atezolizumab plus bevacizumab) index.^46^ Sixth, the categorization of patients into the training and validation cohorts based on the time of data reporting of the centers may have introduced bias as a result of changes in baseline characteristics of the cohort. However, we decided not to change our statistical analysis plan once we knew the results owing to good scientific practice.

It is an important finding that the CABLE score and the other here evaluated scores do not perform well at response prediction. This underscores the need for better biomarkers to sufficiently address this question. While there has been some success in this regard when considering changes in AFP during the first weeks of treatment (either alone^47^^,^^48^ or in combination with ALBI^49^), such an approach is not a true response prediction but rather a response assessment using the serum tumor marker AFP. It also does not enable response prediction before the initiation of treatment which would be highly desirable and relevant for future clinical trials focusing on subgroups of patients with HCC who have a high chance of not responding to a + b. Therefore, the identification and validation of such predictive biomarkers remains an unmet medical need in the field of systemic treatment of HCC and should be pursued with high priority.

In this large multicenter study, we developed and validated the CABLE score to predict the individual prognosis of patients with uHCC undergoing 1L immunotherapy with a + b. To make the CABLE score usable in daily practice we have launched a web-based calculator which computes survival probabilities of this patient group at 6, 12, and 18 months. Further external and geographically diverse validation is needed to confirm the performance of the CABLE score and its incremental value compared with other models, in particular the ALBI score. The validation studies should also focus on subgroup analysis, for example in patients with Child-Pugh A. In the future, the CABLE score may be used for prognostic assessment in clinical practice and as a stratification tool in clinical trials evaluating systemic treatments of HCC.

## Abbreviations

1L, first-line; a + b, atezolizumab and bevacizumab; AFP, alpha-fetoprotein; ALBI, albumin–bilirubin; BCLC, Barcelona Clinic Liver Cancer; CR, complete response; CRAFITY, CRP and AFP in immunotherapy; CRP, C-reactive protein; DCR, disease control rate; ECOG, Eastern Cooperative Oncology Group; EHS, extrahepatic spread; EZ-ALBI, easy-ALBI; GPS, Glasgow Prognostic Score; INR, international normalized ratio; mALBI, modified ALBI; MASH, metabolic dysfunction-associated steatohepatitis; MASLD, metabolic dysfunction-associated steatotic liver disease; MR, mixed response; NLR, neutrophil-to-lymphocyte ratio; ORR, overall response rate; OS, overall survival; PD, progressive disease; PD-L1, programmed death-ligand 1; PFS, progression-free survival; PLR, platelet-to-lymphocyte ratio; PNI, Prognostic Nutritional Index; PR, partial response; SBRT, stereotactic body radiation therapy; SD, stable disease; SIRT, selective internal radiation therapy; TACE, transarterial chemoembolization; TAE, transarterial embolization; TARE, transarterial radioembolization; TRIPOD, Transparent Reporting of a multivariable prediction model for Individual Prognosis or Diagnosis; uHCC, unresectable HCC; VEGF, vascular endothelial growth factor.

## Financial support

This work was not supported by any grant or funding source.

## Authors’ contributions

Performed research: SJG, JG, FA, BS, CL, SL, FS, VJ, TF, VH, NA, CS, KB, JS, SL, JK, CAMF, AD, VZ, FH, NR, NBK, ER, LM, AW, RK, PRG, NGH, SSKV, AT, ET, DTW, JUM, DB, MPR, AG, FPR, LR, DJP, CR, TE, MB, VS, UE, MLB, FFi, MAGC, JF, KS, MV, FB, LSJ, MP, RM, SII, FFo. Contributed to acquisition of data: SJG, JG, FA, BS, CL, SL, FS, VJ, TF, VH, NA, CS, KB, JS, SL, JK, CAMF, AD, VZ, FH, NR, NBK, ER, LM, AW, RK, PRG, NGH, SSKV, AT, ET, DTW, JUM, DB, MPR, AG, FPR, LR, DJP, CR, TE, MB, VS, UE, MLB, FFi, MAGC, JF, KS, MV, FB, LSJ, MP, RM, SII, FFo. Designed the experiments and analyzed the data: PM, IS, SJG, FFo. Contributed reagents/materials/analysis tools: PM, IS, SJG, FFo. Statistical analysis: PM, IS, SJG. Wrote the paper: SJG, FFo, PM.

## Data availability statement

The data that support the findings of this study are available from the corresponding author (FF) upon reasonable request.

## Conflicts of interest

SJG has received travel expenses from Ipsen and Gilead. NA has received travel support and speaker fees from AbbVie and Gilead. FFo has received honoraria for lectures from AstraZeneca, Lilly, MSD, Pfizer, and Roche. He has served as advisory board member to AstraZeneca, BMS, Eisai, and Roche and has received travel support from Merck KGaA and Servier. JUM received honoraria from AstraZeneca, Eisai, MSD, MERZ, AbbVie, and Roche. DB has received lecture and speaker fees from W.L. Gore & Associates GmbH, the Falk Foundation Germany, and a travel grant from Gilead Science. KB has received honoraria for lectures from Ipsen. NHT has received honorarium (to the institution) from Helsinn and is a consultant for AZ, Genentech, and TEMPUS. FPR has received honoraria for lectures, consulting activities, and travel support from the Falk Foundation, AbbVie, Gilead, Ipsen, AstraZeneca, Roche, and Novartis. EDT has served as a paid consultant for AstraZeneca, Bayer, BMS, Eisai, Eli Lilly & Co, Pfizer, Ipsen, and Roche. He has received reimbursement of meeting attendance fees and travel expenses from Arqule, AstraZeneca, BMS, Bayer, Celsion, and Roche, and lecture honoraria from BMS and Falk. In addition, he has received third-party funding for scientific research from Arqule, AstraZeneca, BMS, Bayer, Eli Lilly, and Roche. NBK has received reimbursement of meeting attendance fees and travel expenses from Eisai and a lecture honorarium from Falk and AstraZeneca. KS has received honoraria for lectures from AstraZeneca, MSD. He has served as advisory board member to AstraZeneca, MSD, Servier, Eisai, and Roche. LM has received an honorarium for lecture from Bayer. UE has received honoraria for lectures from AstraZeneca, Eisai, the Falk Foundation, Ipsen, MSD, and Novartis. She has served as advisory board member to AstraZeneca, Bayer, MSD, and Eisai and has received travel support from AstraZeneca and Biotest. LR received consulting fees from AstraZeneca, Basilea, Bayer, BMS, Eisai, Elevar Therapeutics, Exelixis, Genenta, Hengrui, Incyte, Ipsen, IQVIA, Jazz Pharmaceuticals, MSD, Nerviano Medical Sciences, Roche, Servier, Taiho Oncology, Zymeworks; lecture fees from AstraZeneca, Bayer, BMS, Eisai, Incyte, Ipsen, Merck Serono, Roche, Servier; travel expenses from AstraZeneca; research grants (to Institution) from Agios, AstraZeneca, BeiGene, Eisai, Exelixis, Fibrogen, Incyte, Ipsen, Lilly, MSD, Nerviano Medical Sciences, Roche, Servier, Zymeworks. AD received educational support for congress attendance and consultancy fees from Roche, and speaker fees from Roche, AstraZeneca, Eisai, and Chugai. FFi has received travel support from Ipsen, AbbVie, AstraZeneca and speaker’s fees from AbbVie, MSD, Ipsen, Astra. MP received speaker honoraria from Bayer, BMS, Eisai, Ipsen, Lilly, MSD, and Roche; he is a consultant for AstraZeneca, Bayer, BMS, Eisai, Ipsen, Lilly, MSD, and Roche; he received grants from Roche and BMS; he received travel support from Bayer, BMS, Ipsen, and Roche. MPR has received consulting fees from Bayer, BMS, Boehringer-Ingelheim, Eisai, Gilead, Intercept-Advanz, Ipsen, Lilly, MSD, Roche, Sanofi and was an investigator for Bayer, BMS, Boehringer-Ingelheim, Exelixis, Eisai, Falk, Gilead, Lilly, Ipsen, Novartis, and Roche. DJP received lecture fees from Bayer Healthcare, Eisai, BMS, Roche, Boston Scientific, travel expenses from BMS and Bayer Healthcare; consulting fees for Mina Therapeutics, DaVolterra, Mursla, Ipsen, Exact Sciences, Avamune, Eisai, Roche, Starpharma, LiFT biosciences and AstraZeneca; received research funding (to institution) from MSD, GSK, and BMS. LSJ received speaker honoraria from Falk Foundation, Boston Scientific, AbbVie, and AstraZeneca. She is consultant for Boston Scientific and AstraZeneca. She received travel support from Biotest and AbbVie. MAGC has contributed to advisory boards for Roche, Eisai, MSD, BMS, AZ, Amgen and Servier (these activities have no potential conflicts of interest with the manuscript). AW received compensations as a member of scientific advisory boards for Bayer, BMS, Eisai, and Sanofi and served as a speaker for Leo Pharma, Eisai, Ipsen, and Roche and received travel support from Merck and Servier. RK has received consultancy fees from Boston Scientific, Bristol-Myers Squibb, Guerbet, Roche, and SIRTEX, and lecture fees from AstraZeneca, BTG, Eisai, Guerbet, Ipsen, Roche, Siemens, SIRTEX, and MSD Sharp & Dohme (none are related to this work). PRG received honoraria for lectures and advisory boards from Bayer, BMS, MSD, AstraZeneca, Lilly, Ipsen, Roche, Eisai, Guerbet, and Boston Scientific. SII served as advisory board member for AstraZeneca and received consulting fees from AstraZeneca. All other authors disclose no potential financial or non-financial conflict of interests.

Please refer to the accompanying ICMJE disclosure forms for further details.
