# Supplementary material for: Evaluation of prognostic scores in patients with HCC undergoing first-line immunotherapy with atezolizumab and bevacizumab
Source: JHEP Rep. 2024 Dec 5;7(3):101295. doi: 10.1016/j.jhepr.2024.101295 (PMC11889551; doi:10.1016/j.jhepr.2024.101295)
Supplement: Multimedia component 1 [file mmc1.pdf]

Evaluation of prognostic scores in patients with HCC undergoing first-line immunotherapy with atezolizumab and bevacizumab

Simon Johannes Gairing, Philipp Mildenberger, Jennifer Gile, Fabian Artusa, Bernhard Scheiner, Catherine Leyh, Sabine Lieb, Friedrich Sinner, Vincent Jörg, Thorben Fruendt, Vera Himmelsbach, Nada Abedin, Cennet Sahin, Katrin Böttcher, Jasmin Schuhbaur, Simon Labuhn, James Korolewicz, Claudia A.M. Fulgenzi, Antonio D'Alessio, Valentina Zanuso, Florian Hucke, Natascha Röhlen, Najib Ben Khaled, Eleonora Ramadori, Lukas Müller, Arndt Weinmann, Roman Kloeckner, Peter Robert Galle, Nguyen H. Tran, Sudhakar K. Venkatesh, Andreas Teufel, Matthias Ebert, Enrico N. De Toni, Dirk-Thomas Waldschmidt, Jens U. Marquardt, Dominik Bettinger, Markus Peck-Radosavljevic, Andreas Geier, Florian P. Reiter, Lorenza Rimassa, David J. Pinato, Christoph Roderburg, Thomas Ettrich, Michael Bitzer, Veit Scheble, Ursula Ehmer, Marie-Luise Berres, Fabian Finkelmeier, Maria Angeles Gonzalez-Carmona, Johann von Felden, Kornelius Schulze, Marino Venerito, Florian van Bömmel, Leonie S. Jochheim, Matthias Pinter, Raphael Mohr, Sumera I. Ilyas, Irene Schmidtmann, Friedrich Foerster

Table of contents

Supplementary methods 2

Supplementary tables 7

Supplementary figures 22

TRIPOD checklist 26

Supplementary methods

For statistical analysis, R (version 4.1.3, a language and environment for statistical computing. R Foundation for Statistical Computing, Vienna, Austria. https://www.R-project.org/.), and RStudio (version 2023.06.2+561, Integrated Development for R. RStudio, PBC, Boston, MA, http://www.rstudio.com/.) were used.

Study design and patients

The training set included patients from Berlin (DE), Essen (DE), Mainz (DE), Frankfurt (DE), Ulm (DE), Freiburg (DE), Munich (TUM, DE), Bonn (DE), Magdeburg (DE), Aachen (DE), Vienna (AT), and Rochester (US). The external validation set was derived from Lübeck (DE), Würzburg (DE), Cologne (DE), Düsseldorf (DE), Mannheim (DE), Tübingen (DE), Munich (LMU, DE), Leipzig (DE), Hamburg (DE), Klagenfurt (AT), Milan (IT), London (UK).

All patients ≥ 18 years old with a radiologically and/or histologically confirmed uHCC undergoing immunotherapy with a+b were screened for eligibility. Exclusion criteria were: a+b not 1L systemic therapy, Child-Pugh C, ECOG (Eastern Cooperative Oncology Group) status > 2, no follow-up data available, simultaneous brachytherapy, no baseline laboratory values within 30 days prior to the treatment start available, mixed hepatocellular/cholangiocellular carcinoma histology, simultaneous chemotherapy due to second malignancy, and a history of liver transplantation (details on how many patients are excluded respectively are given in **Fig. 1**). Some patient data from some centers have been used as part of other research projects [1–7].

Outcome

The outcome of interest was death from any cause. Information on patients' vital status was obtained from hospital records. Baseline was defined at the start of immunotherapy with a+b. Patients still alive at the time of data cut-off were censored on the day of last contact. Overall survival was calculated from the day of the first cycle of a+b until death or last contact. Patients who underwent liver transplantation following immunotherapy with a+b were censored on the day of transplantation. As only two patients underwent liver transplantation, no competing risk model was used.

Candidate predictors

Candidate predictors included patient characteristics, tumor characteristics and several laboratory values all of which were determined at baseline. **Table S1** provides an overview of variables which have been extracted for all patients. The candidate predictors were selected based on available evidence in the literature and because they are easily and cost-effective available for every HCC patient before treatment start. If laboratory values were not determined at the day of the first a+b cycle, values up to 30 days before treatment start were accepted. If multiple measurements for a laboratory value were available within 30 days, the one closest to the day of the first cycle was selected. The assessment of predictors was not blinded. The presence of cirrhosis was determined by histology or typical findings on imaging, endoscopy and/or medical history.

Response assessment

Tumor response following treatment with a+b was assessed by computed tomography or magnetic resonance imaging. Response was graded as complete response (CR), partial response (PR), stable disease (SD), progressive disease (PD) or mixed response (MR) according to the modified Response Evaluation Criteria in Solid Tumors

(mRECIST) or, if not available, according to the local standard. MR was defined as progression at one site, while shrinking at another. The objective response rate (ORR) included patients with CR and PR, the disease control rate (DCR) included patients with CR, PR, SD, and MR.

Sample size

Given the retrospective study design and the routinely collected data, all patients meeting the eligibility criteria without fulfilling exclusion criteria were included. Consequently, a formal sample size calculation was not conducted.

Calculation of scores

The ALBI score was calculated as follows: ALBI = ($\log_{10}$ bilirubin [μmol/L] × 0.66) + (albumin [g/L] × -0.085) [8]. Regarding the CRAFITY score, CRP levels ≥1 mg/dl and AFP levels AFP ≥100 ng/ml were each scored with one point, yielding three risk groups [1]. The PNI was calculated using the following equation: PNI = (albumin [g/L]) + (0.005 × total lymphocyte count per mm^3) [9, 10]. The NLR was calculated by dividing the total neutrophil count per nL by the total lymphocyte count per nL, the PLR by dividing the platelet count per nL by the total lymphocyte count per nL [11]. The EZ-ALBI score was calculated by (bilirubin [mg/dl] – (9 × albumin [g/dL])) [12]. mALBI grades were defined as described in Hiraoka et al. [13]. The Glasgow Prognostic Score (GPS) was calculated as detailed in [14]. Seven centers did not provide Child-Pugh stages for patients without cirrhosis. Here, the Child-Pugh stage was calculated using albumin, INR, and bilirubin.

Statistical analysis

For descriptive statistics, continuous data are provided as median with range (min – max). Categorical data are given as frequencies with percentages. For the comparison of the training and validation set, a Chi square test was used for categorial data and a Mann-Whitney U test or Students t-test for continuous data depending on data distribution. The reverse Kaplan-Meier method was used to estimate median follow-up time.

Modification of candidate predictors

AFP, creatinine, and neutrophils had few extreme values and were therefore trimmed as follows: AFP: 13 cases with an AFP level > 100,000 ng/ml were set to 100,000 ng/ml; creatinine: 10 cases with a creatinine level > 3 mg/dl were set to 3 mg/dl; neutrophils: 2 cases with absolute neutrophil counts > 20/nl were set to 20/nl. AFP, INR, bilirubin, creatinine, CRP, and platelets were log_{10}-transformed due to their skewed distributions.

Missing data

Candidate predictors with less than 10% of missing values were imputed in 5 multiple imputations. This was done with predictive mean matching in the R-package mice. The proportion of missing data in each candidate predictor are given in **Table 1**.

The variables with more than 0% but ≤ 10% missingness were INR, albumin, bilirubin, creatinine, AFP, neutrophils and platelets (**Table 1**). These variables, along with age, sex, center, cirrhosis, pretreatment, ECOG, extrahepatic spread (EHS), macrovascular invasion (MVI), time-to-event and status were used to derive missing values in this context. CRP, lymphocytes and monocytes had more than 10% missing values in the training set. Thus, these variables were neither imputed nor included in the initial model building. After building a preliminary model based on the imputed data set, we analyzed

whether these variables could enhance the predictive performance on the complete-case training set. This approach was similar to that of Åberg et al. when building the CLivD score [15]. Regarding the outcome (death from any cause), no data were missing.

Supplementary tables

Table S1: Candidate predictors at baseline.

Patient characteristics	Tumor characteristics	Laboratory values
Gender (male (1), female (2))	Presence of extrahepatic spread (0/1)	Alpha-fetoprotein (AFP), ng/ml
Age (years)	Presence of macrovascular invasion (0/1)	International Normalized Ratio (INR)
Etiology		Albumin (g/dl)
Presence of cirrhosis (0/1)		Bilirubin (mg/dl)
Pretreatments		Creatinine (mg/dl)
ECOG performance status (0, 1, 2, 3, 4)		C-reactive protein (CRP), mg/l
Child-Pugh stage (A, B, C)		Neutrophils (X/nl and in %)
BCLC stage (0, A, B, C, D)		Lymphocytes (X/nl and in %)
		Monocytes (X/nl and in %)
		Platelets (X/nl)

Table S2. Reasons for discontinuation of a+b.

	**Total cohort (n = 683)**
Ongoing therapy	173 (25.3%)
Progression	274 (40.1%)
Death	87 (12.7%)
Toxicity	58 (8.5%)
Patient request	22 (3.2%)
Unknown	22 (3.2%)
Decompensation/liver function decline	10 (1.5%)
Deterioration of general health	7 (1.0%)
Lost to follow-up	7 (1.0%)
CR	6 (0.9%)
Secondary surgical resection	5 (0.7%)
Toxicity/progression	3 (0.4%)
Bleeding and/or thrombotic event	3 (0.4%)
Liver transplantation	2 (0.3%)
Toxicity/death	1 (0.1%)
Toxicity/patient request	1 (0.1%)
Planned liver transplantation	1 (0.1%)
Insurance issues	1 (0.1%)

Abbreviations: CR, complete response.

Table S3. Radiological response in the training and validation cohort.

	Total cohort (N=683)	Training set (N=526)	Validation set (N=157)
**Tumor response**			
CR	24 (4.9%)	18 (5.2%)	6 (4.2%)
PR	129 (26.6%)	91 (26.5%)	38 (26.8%)
SD	187 (38.6%)	132 (38.5%)	55 (38.7%)
PD	140 (28.9%)	97 (28.3%)	43 (30.3%)
MR	5 (1.0%)	5 (1.5%)	0 (0%)
Missing	198 (29.0%)	183 (34.8%)	15 (9.6%)
**ORR**			
SD+PD	332 (68.5%)	234 (68.2%)	98 (69.0%)
CR+PR	153 (31.5%)	109 (31.8%)	44 (31.0%)
Missing	198 (29.0%)	183 (34.8%)	15 (9.6%)
**DCR**			
PD	140 (28.9%)	97 (28.3%)	43 (30.3%)
CR+PR+SD	345 (71.1%)	246 (71.7%)	99 (69.7%)
Missing	198 (29.0%)	183 (34.8%)	15 (9.6%)

Abbreviations: CR, complete response; PR, partial response; SD, stable disease; PD, progressive disease; MR, mixed response; ORR, objective response rate; DCR, disease control rate.

Table S4. Univariable Cox proportional hazards models.

Variable	Explained Deviance	Chi² test (p-value)
Age (years)	2.77%	0.4
Gender (male vs female)	2.20%	0.2
Etiology		
9 categories[a]	3.03%	0.2
Nonviral vs viral vs mixed etiology	1.93%	0.8
MASLD/MASH vs others	1.93%	0.3
Cirrhosis (yes vs no)	2.48%	0.041
Pretreatment (yes vs no)	2.65%	<0.001
ECOG	5.65%	<0.001
BCLC	3.92%	<0.001
Child-Pugh stage	6.42%	<0.001
EHS (yes vs no)	2.48%	0.002
MVI (yes vs no)	3.39%	<0.001
AFP, log10 (ng/ml)	3.45%	<0.001
Albumin (g/dl)	9.30%	<0.001
Bilirubin, log10 (mg/dl)	6.61%	<0.001
Creatinine, log10 (mg/dl)	2.56%	0.002
CRP, log10 (mg/l)	7.58%	<0.001
INR, log10	3.39%	<0.001
Platelets, log10 (/nl)	2.66%	0.007
Neutrophils (/nl)	3.48%	<0.001
Lymphocytes (/nl)	5.70%	<0.001
Monocytes (/nl)	2.19%	<0.001

[a]unknown/no underlying liver disease, HBV, HCV, alcohol, MASLD/MASH/metabolic, mixed etiology (viral + non-viral), mixed etiology (only non-viral), mixed (only viral), other etiologies. A Chi² test was carried out to assess statistical significance.

Table S5. Adjusted Cox PH model of the Model$_{i}$.

Variables	Beta[a]	95% CI	p-value
INR_{log10}	1.5	-0.28, 3.3	0.10
$Bilirubin_{log10}$	1.3	0.78, 1.8	<0.001
Pretreatment			
no	—	—	
yes	-0.29	-0.56, -0.02	0.035
ECOG			
0	—	—	
1	0.50	0.21, 0.79	<0.001
2	0.94	0.43, 1.5	<0.001
EHS			
no	—	—	
yes	0.47	0.20, 0.73	<0.001
s(Albumin)			<0.001
s($Creatinine_{log10}$)			0.032

Adjusted Cox PH model. [a] log(HR). Abbreviations: CI: confidence interval; INR: International Normalized Ratio; ECOG: Eastern Cooperative Oncology Group; EHS: extrahepatic spread; s: smoothing spline.

Table S6. Baseline characteristics of the complete-case training set.

	Overall (N=320)
**Age (years)**	
Median [Min, Max]	67.0 [25.0, 89.5]
**Gender**	
male	260 (81.3%)
female	60 (18.8%)
**ECOG**	
0	142 (44.4%)
1	152 (47.5%)
2	26 (8.1%)
**Cirrhosis**	
no	67 (20.9%)
yes	253 (79.1%)
**Etiology**	
unknown/no liver disease	36 (11.3%)
HBV	41 (12.8%)
HCV	53 (16.6%)
Harmful alcohol use	83 (25.9%)
MASLD/MASH	64 (20.0%)
Mixed (viral and non-viral)	16 (5.0%)
Mixed (only non-viral)	15 (4.7%)
Mixed (only viral)	1 (0.3%)
Other	11 (3.4%)
**BCLC**	
A	6 (1.9%)
B	70 (21.9%)
C	244 (76.3%)
**Pretreatment**	
no	148 (46.3%)
yes	172 (53.8%)
**History of surgery**	
no	255 (79.7%)
yes	65 (20.3%)
**History of ablation**	
no	284 (88.8%)
yes	36 (11.3%)
**History of TACE/TAE**	
no	219 (68.4%)
yes	101 (31.6%)
**History of SIRT/TARE**	
no	291 (90.9%)
yes	29 (9.1%)
**History of radiatio/SBRT**	
no	313 (97.8%)
yes	7 (2.2%)
**History of brachytherapy**	
no	310 (96.9%)
yes	10 (3.1%)
**Macrovascular invasion (MVI)**	
no	161 (50.3%)
yes	159 (49.7%)
**Extrahepatic spread (EHS)**	
no	199 (62.2%)
yes	121 (37.8%)
**Child-Pugh**	
A	229 (71.6%)
B	91 (28.4%)

	Overall (N=320)
**ALBI score**	
Median [Min, Max]	-2.31 [-3.61, -0.733]
**ALBI grade**	
1	103 (32.2%)
2	200 (62.5%)
3	17 (5.3%)
**mALBI grade**	
1	103 (32.2%)
2a	64 (20.0%)
2b	136 (42.5%)
3	17 (5.3%)
**EZ-ALBI**	
Median [Min, Max]	-31.4 [-45.2, -14.6]
**CRAFITY score**	
low	92 (28.8%)
intermediate	138 (43.1%)
high	90 (28.1%)
**GPS**	
GPS 0	113 (35.3%)
GPS 1	125 (39.1%)
GPS 2	82 (25.6%)
**PNI**	
Median [Min, Max]	42.3 [23.0, 63.1]
**NLR**	
Median [Min, Max]	3.69 [0.337, 117]
**PLR**	
Median [Min, Max]	152 [33.3, 3300]
**Bilirubin (mg/dl)**	
Median [Min, Max]	0.827 [0.160, 12.2]
**Albumin (g/dl)**	
Median [Min, Max]	3.65 [1.70, 5.16]
**INR**	
Median [Min, Max]	1.16 [0.900, 3.90]
**Creatinine (mg/dl)**	
Median [Min, Max]	0.870 [0.386, 8.00]
**CRP (mg/l)**	
Median [Min, Max]	11.2 [0.400, 279]
**AFP (ng/ml)**	
Median [Min, Max]	79.0 [1.00, 2070000]
**Platelets per nl**	
Median [Min, Max]	160 [34.0, 760]
**Neutrophils per nl**	
Median [Min, Max]	4.15 [0.550, 15.8]
**Lymphocytes per nl**	
Median [Min, Max]	1.08 [0.0700, 3.59]
**Monocytes per nl**	
Median [Min, Max]	0.584 [0.0490, 2.08]
Missing	1 (0.3%)

Abbreviations: ECOG, Eastern Cooperative Oncology Group; HBV, hepatitis B virus; HCV, hepatitis C virus; MASLD, Metabolic dysfunction–associated steatotic liver disease; MASH, Metabolic dysfunction–associated steatohepatitis; BCLC, Barcelona Clinic Liver Cancer; HCC; hepatocellular carcinoma, TACE, transarterial chemoembolization; TAE, transarterial embolization; SIRT, selective internal radiation therapy; TARE, transarterial radioembolization; SBRT, stereotactic body radiation therapy; ALBI, albumin-bilirubin; mALBI, modified ALBI; EZ-ALBI, easy-ALBI; CRAFITY, CRP and AFP in immunotherapy; GPS, Glasgow Prognostic Score; PNI, prognostic nutritional index; NLR, neutrophil-to-

lymphocyte ratio, PLR, platelet-to-lymphocyte ratio; INR, International Normalized Ratio; CRP, C-reactive protein; AFP, alpha-fetoprotein.

Table S7. Comparison of variables included in different prognostic models.

	Albumin	Bilirubin	Lymphocytes	Neutrophils	Platelets	CRP	AFP	Creatinine	INR	ECOG	EHS	Pretreatment
**CABLE score**	X	X	X			X				X	X	
**Model**$_i$	X	X						X	X	X	X	X
**ALBI [8]**	X	X										
**EZ-ALBI [12]**	X	X										
**mALBI [13]**	X	X										
**PNI [9, 10]**	X		X									
**CRAFITY [1]**						X	X					
**NLR [11]**			X	X								
**PLR [11]**			X		X							
**GPS [14]**	X					X						

Abbreviations: ALBI, albumin-bilirubin; EZ-ALBI, easy ALBI; mALBI, modified ALBI; PNI, prognostic nutritional index; CRAFITY, CRP and AFP in Immunotherapy; NLR, neutrophiles to lymphocytes ratio; PLR, platelets to lymphocytes ratio; GPS, Glasgow Prognostic Score; CRP, C-reactive protein; AFP, alpha-fetoprotein; INR, International Normalized Ratio; ECOG, Eastern Cooperative Oncology Group; EHS, extrahepatic spread.

Table S8. Time-dependent AUC of the CABLE score and $Model_i$ compared to several other prognostic models after 6, 12 and 18 months in the training set (internal validation).

Models	6 months	12 months	18 months
**CABLE score**	0.774	0.792	0.815
**$Model_i$**	0.761	0.75	0.793
**ALBI**	0.719	0.715	0.74
**EZ-ALBI**	0.715	0.715	0.732
**ALBI grades**	0.654	0.635	0.653
**mALBI**	0.677	0.660	0.679
**PNI**	0.722	0.735	0.774
**CRAFITY**	0.676	0.693	0.607
**GPS**	0.626	0.689	0.702
**NLR**	0.656	0.643	0.721
**PLR**	0.607	0.62	0.647

Abbreviations: CABLE: CRP, albumin, bilirubin, lymphocytes, ECOG and EHS; $Model_i$: Cox PH model derived from the imputed training set; ALBI: albumin-bilirubin; PNI: prognostic nutritional index; CRAFITY: CRP and AFP in immunotherapy; EZ-ALBI: Easy-ALBI; mALBI: modified ALBI; GPS: Glasgow prognostic score; NLR: neutrophil-to-lymphocyte ratio; PLR: platelet-to-lymphocyte ratio.

Table S9. Time-dependent AUC of the CABLE score compared to several other prognostic models after 6, 12 and 18 months in the validation set (external validation).

Models	6 months	12 months	18 months
**CABLE score**	0.835	0.713	0.754
**$Model_i$**	0.845	0.714	0.721
**ALBI**	0.839	0.735	0.745
**EZ-ALBI**	0.839	0.743	0.762
**PNI**	0.764	0.732	0.752
**CRAFITY**	0.745	0.634	0.708
**ALBI grades**	0.748	0.675	0.726
**mALBI**	0.816	0.728	0.748
**GPS**	0.804	0.658	0.651
**NLR**	0.542	0.529	0.586
**PLR**	0.433	0.485	0.577

Abbreviations: CABLE: CRP, albumin, bilirubin, lymphocytes, ECOG and EHS; $Model_i$: Cox PH model derived from the imputed training set; ALBI: albumin-bilirubin; PNI: prognostic nutritional index; CRAFITY: CRP and AFP in immunotherapy; EZ-ALBI: Easy-ALBI; mALBI: modified ALBI; GPS: Glasgow prognostic score; NLR: neutrophil-to-lymphocyte ratio; PLR: platelet-to-lymphocyte ratio.

Table S10. Uno's C indices for the validation set with bootstrapped confidence intervals.

Model	6 months	12 months	18 months
CABLE score	0.778 [0.703-0.852]	0.706 [0.627-0.786]	0.706 [0.643-0.769]
$Model_i$	0.79 [0.702-0.879]	0.711 [0.628-0.795]	0.703 [0.629-0.778]
ALBI	0.783 [0.711-0.855]	0.722 [0.642-0.803]	0.719 [0.645-0.793]
EZ-ALBI	0.785 [0.694-0.875]	0.723 [0.654-0.793]	0.72 [0.644-0.796]
ALBI grade	0.714 [0.634-0.795]	0.659 [0.591-0.727]	0.665 [0.617-0.714]
mALBI grade	0.766 [0.695-0.838]	0.706 [0.631-0.781]	0.704 [0.636-0.771]
PNI	0.722 [0.624-0.82]	0.703 [0.634-0.772]	0.702 [0.631-0.774]
CRAFITY	0.719 [0.622-0.817]	0.62 [0.543-0.697]	0.629 [0.559-0.7]
GPS	0.767 [0.689-0.846]	0.675 [0.606-0.743]	0.653 [0.593-0.714]
NLR	0.53 [0.375-0.685]	0.518 [0.413-0.624]	0.532 [0.417-0.647]
PLR	0.565 [0.434-0.696]	0.535 [0.422-0.647]	0.513 [0.413-0.612]

Abbreviations: CABLE: CRP, albumin, bilirubin, lymphocytes, ECOG and EHS; $Model_i$: Cox PH model derived from the imputed training set; ALBI: albumin-bilirubin; PNI: prognostic nutritional index; CRAFITY: CRP and AFP in immunotherapy; EZ-ALBI: Easy-ALBI; mALBI: modified ALBI; GPS: Glasgow prognostic score; NLR: neutrophil-to-lymphocyte ratio; PLR: platelet-to-lymphocyte ratio.

Table S11. Performance of the CABLE score in relevant subgroups of the validation cohort.

Subgroup	n	HR CABLE (95%-CI)
All	157	1.75 [1.397; 2.192]
Child A	114	1.915 [1.274; 2.88]
Child B	43	1.086 [0.75; 1.572]
EHS: no	105	1.641 [1.272; 2.116]
EHS: yes	52	2.173 [1.321; 3.575]
MVI: no	97	1.86 [1.284; 2.694]
MVI: yes	60	1.709 [1.256; 2.326]
ECOG: 0	92	1.89 [1.264; 2.825]
ECOG: 1+2	65	1.677 [1.185; 2.373]
Etiology: non-viral	120	1.669 [1.289; 2.161]
Etiology: viral	31	2.38 [1.364; 4.152]

Abbreviations: HR: hazard ratio; CABLE: CRP, albumin, bilirubin, lymphocytes, ECOG and EHS; CI: confidence interval; EHS: extrahepatic spread; MVI: macrovascular invasion; ECOG: Eastern Cooperative Oncology Group.

Table S12. Uno's C indices for the training set in the subgroup of patients with Child-Pugh A with bootstrapped confidence intervals.

Model	3 months	6 months	12 months	18 months
**CABLE score**	0.769 [0.679-0.859]	0.727 [0.649-0.806]	0.727 [0.67-0.783]	0.718 [0.648-0.787]
**$Model_i$**	0.721 [0.644-0.797]	0.72 [0.647-0.794]	0.704 [0.644-0.763]	0.701 [0.642-0.76]
**ALBI**	0.657 [0.556-0.757]	0.651 [0.569-0.732]	0.641 [0.578-0.705]	0.645 [0.577-0.713]
**EZ-ALBI**	0.637 [0.511-0.763]	0.636 [0.542-0.731]	0.627 [0.558-0.697]	0.629 [0.57-0.688]
**ALBI grade**	0.577 [0.49-0.663]	0.593 [0.507-0.679]	0.584 [0.524-0.643]	0.593 [0.538-0.648]
**mALBI grade**	0.592 [0.485-0.699]	0.611 [0.533-0.689]	0.593 [0.523-0.663]	0.605 [0.541-0.67]
**PNI**	0.66 [0.553-0.768]	0.649 [0.563-0.735]	0.655 [0.579-0.732]	0.649 [0.584-0.714]
**CRAFITY**	0.731 [0.64-0.821]	0.697 [0.626-0.769]	0.672 [0.617-0.727]	0.628 [0.57-0.686]
**GPS**	0.603 [0.513-0.693]	0.576 [0.512-0.639]	0.591 [0.537-0.645]	0.584 [0.532-0.636]
**NLR**	0.713 [0.631-0.795]	0.629 [0.547-0.711]	0.611 [0.536-0.686]	0.596 [0.516-0.677]
**PLR**	0.67 [0.53-0.81]	0.632 [0.557-0.707]	0.613 [0.543-0.683]	0.595 [0.537-0.652]

Abbreviations: CABLE: CRP, albumin, bilirubin, lymphocytes, ECOG and EHS; $Model_i$: Cox PH model derived from the imputed training set; ALBI: albumin-bilirubin; PNI: prognostic nutritional index; CRAFITY: CRP and AFP in immunotherapy; EZ-ALBI: Easy-ALBI; mALBI: modified ALBI; GPS: Glasgow prognostic score; NLR: neutrophil-to-lymphocyte ratio; PLR: platelet-to-lymphocyte ratio.

Table S13. Logistic regression models for the prediction of ORR and DCR in the validation cohort.

Model	AUC (ORR)	AUC (DCR)
CABLE score	0.614 [0.517;0.711]	0.613 [0.509;0.716]
$Model_i$	0.625 [0.529;0.721]	0.592 [0.48;0.704]
ALBI	0.644 [0.545;0.742]	0.636 [0.535;0.737]
EZ-ALBI	0.648 [0.549;0.747]	0.658 [0.559;0.757]
ALBI grade	0.595 [0.508;0.683]	0.627 [0.536;0.717]
mALBI grade	0.599 [0.508;0.69]	0.633 [0.538;0.728]
PNI	0.631 [0.53;0.733]	0.653 [0.558;0.748]
CRAFITY	0.565 [0.47;0.659]	0.598 [0.503;0.694]
GPS	0.606 [0.529;0.682]	0.623 [0.531;0.715]
NLR	0.608 [0.506;0.71]	0.471 [0.366;0.576]
PLR	0.59 [0.491;0.69]	0.514 [0.408;0.62]

Logistic regression models. Abbreviations: ORR: objective response rate; DCR: disease control rate; CABLE: CRP, albumin, bilirubin, lymphocytes, ECOG and EHS; $Model_i$: Cox PH model derived from the imputed training set; ALBI: albumin-bilirubin; PNI: prognostic nutritional index; CRAFITY: CRP and AFP in immunotherapy; EZ-ALBI: Easy-ALBI; mALBI: modified ALBI; GPS: Glasgow prognostic score; NLR: neutrophil-to-lymphocyte ratio; PLR: platelet-to-lymphocyte ratio.

Table S14. Univariable models to evaluate the association of variables with response to a+b (ORR).

Variable	Explained Deviance	Chi² test (p-value)
Age (years)	2.39 %	0.458
Gender (male vs female)	2.24 %	0.9
Etiology		
9 categories[a]	3.14 %	0.8
Nonviral vs viral vs mixed etiology	2.4 %	0.745
MASLD/MASH vs others	2.26 %	0.58
Cirrhosis (yes vs no)	2.53 %	0.353
Pretreatment (yes vs no)	2.87 %	0.164
**ECOG**	**4.11 %**	**0.0249**
BCLC	3.05 %	0.242
Child-Pugh stage	2.57 %	0.21
EHS (yes vs no)	2.17 %	0.254
**MVI (yes vs no)**	**3.39 %**	**0.0107**
AFP, log10 (ng/ml)	4.52 %	0.253
Albumin (g/dl)	5.17 %	0.197
Bilirubin, log10 (mg/dl)	2.45 %	0.35
Creatinine, log10 (mg/dl)	3.21 %	0.386
CRP, log10 (mg/l)	2.4 %	0.0942
INR, log10	2.3 %	0.442
**Platelets, log10 (/nl)**	**7.05 %**	**0.0157**
**Neutrophils (/nl)**	**5.04 %**	**0.0027**
Lymphocytes (/nl)	1.11 %	0.196
Monocytes (/nl)	0.44 %	0.397

[a]unknown/no underlying liver disease, HBV, HCV, alcohol, MASLD/MASH/metabolic, mixed etiology (viral + non-viral), mixed etiology (only non-viral), mixed (only viral), other etiologies. A Chi² test was used for assessing statistical significance.

Supplementary figures

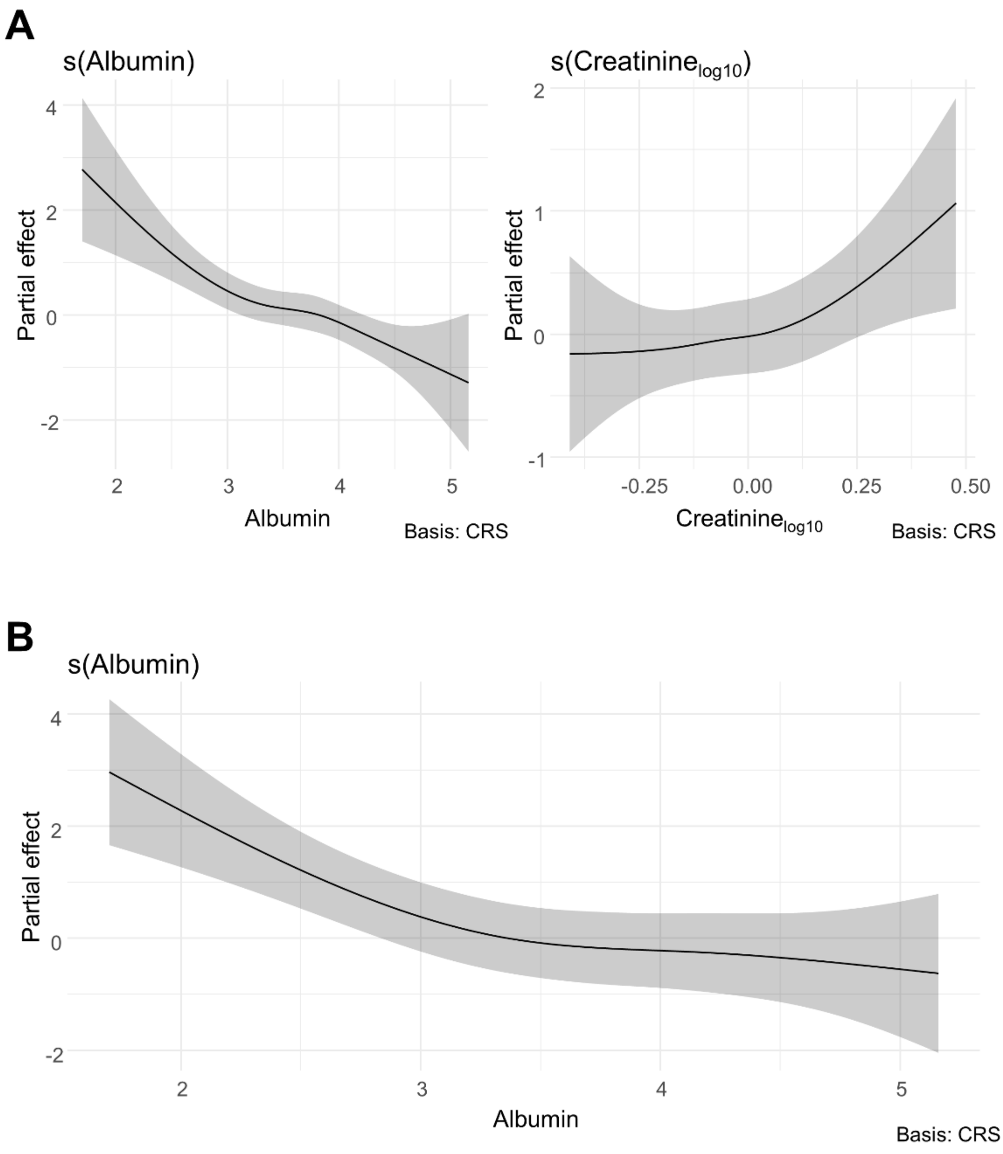

Fig. S1: Smoothing splines of albumin and creatinine$_{log10}$ in the Model$_i$ (A) and of albumin in the CABLE score (B).

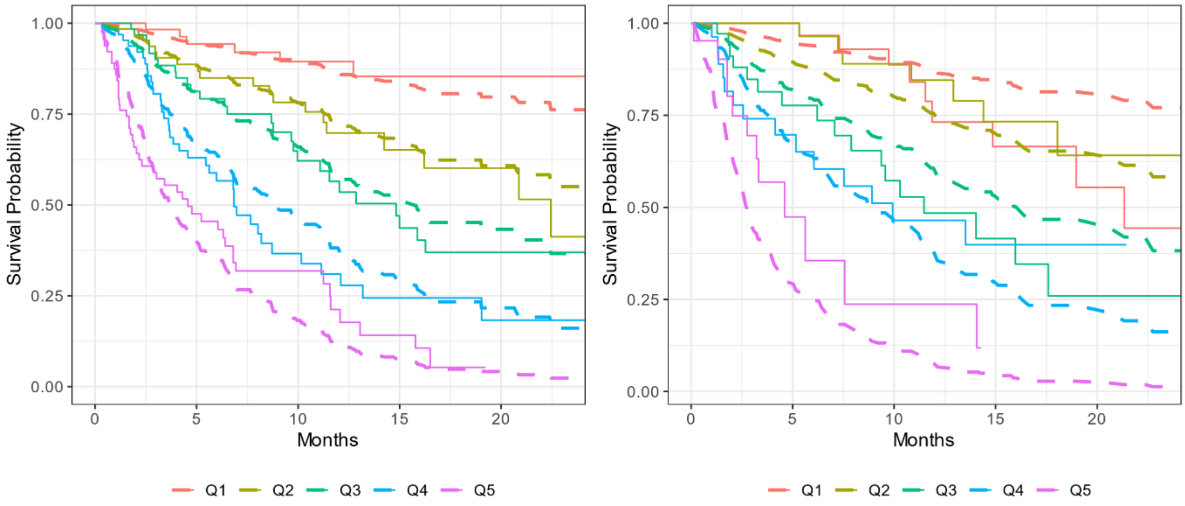

Fig S2. Calibration plots showing the observed (solid line) vs. predicted (dashed line) OS in the training set (left plot) and the test set (right plot) after stratifying patients into Quintiles (Q1-Q5) according to their prognostic score calculated by the CABLE score.

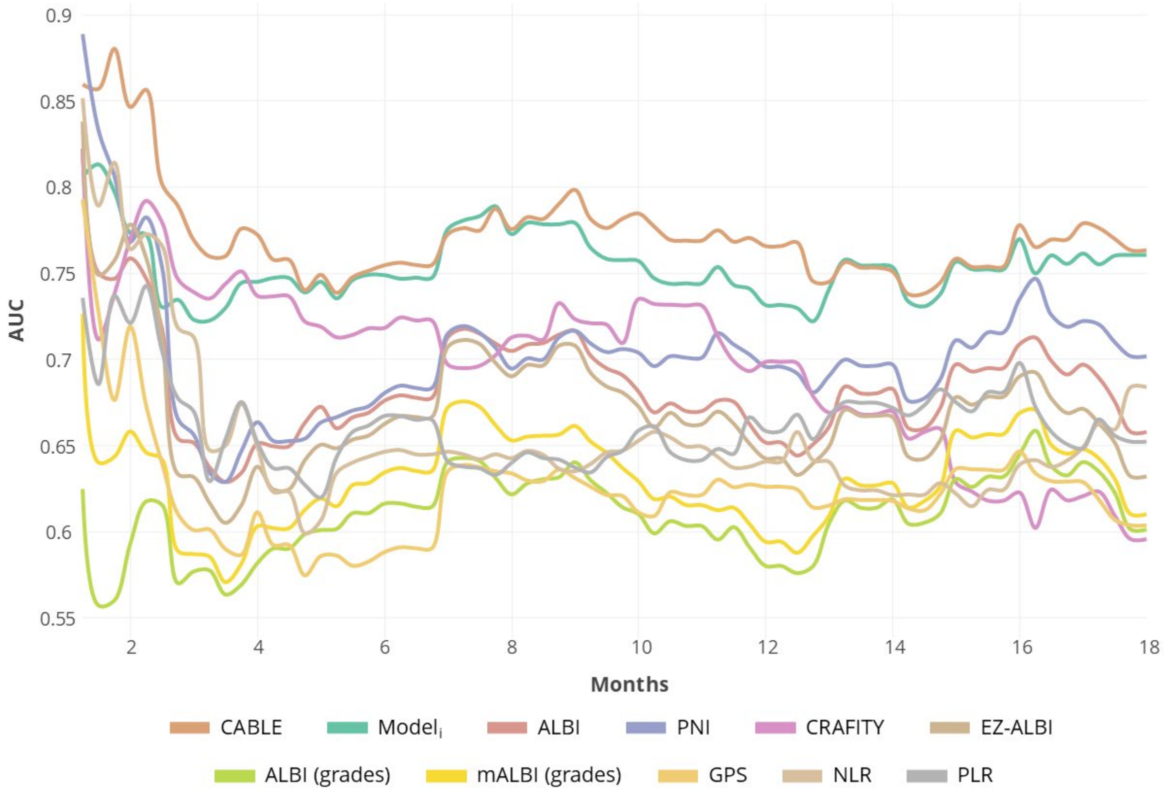

Fig. S3. Time-dependent AUC of the CABLE score and Model$_i$ in comparison with established prediction models in the training set in the subgroup of patients with Child-Pugh A.

Abbreviations: CABLE: **C**RP, **a**lbumin, **b**ilirubin, **l**ymphocytes, **E**COG and **E**HS; Model$_i$: Cox PH model derived from the imputed training set; ALBI: albumin-bilirubin; PNI: prognostic nutritional index; CRAFITY: CRP and AFP in immunotherapy; EZ-ALBI: Easy-ALBI; mALBI: modified ALBI; GPS: Glasgow prognostic score; NLR: neutrophil-to-lymphocyte ratio; PLR: platelet-to-lymphocyte ratio.

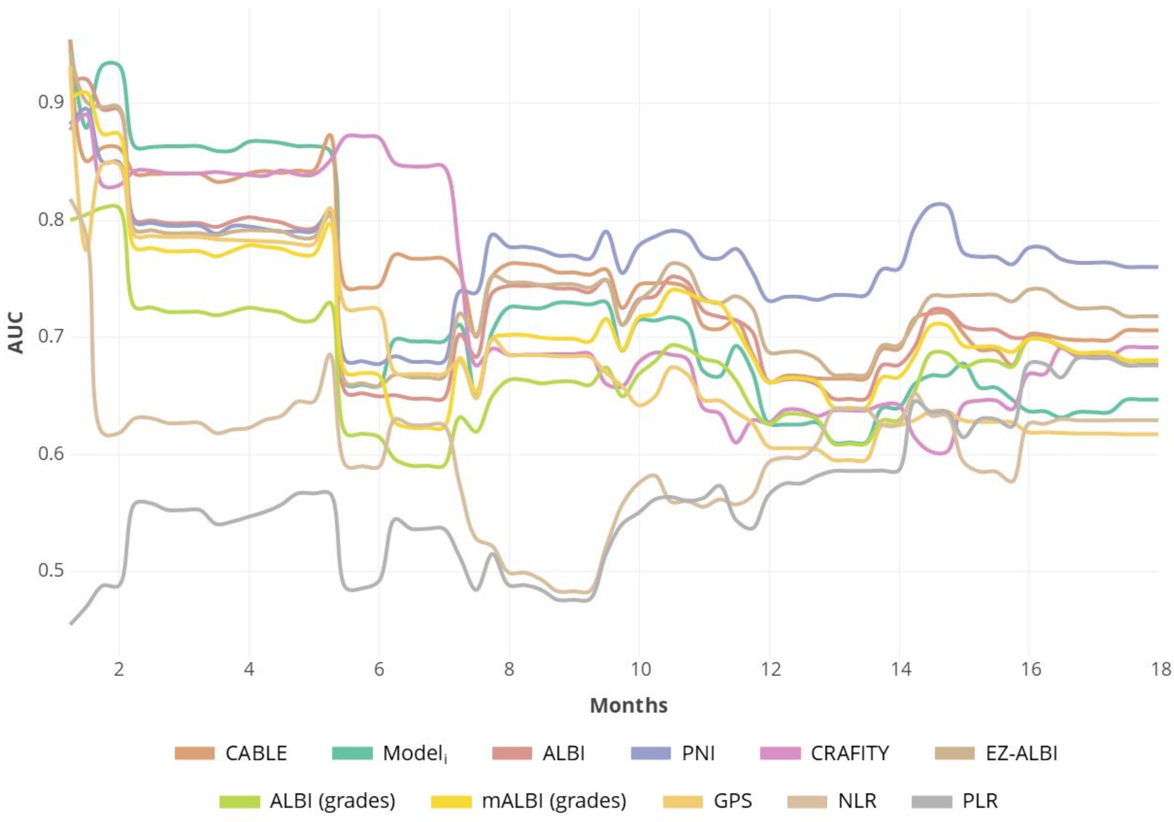

Fig. S4. Time-dependent AUC of the CABLE score and Model$_i$ in comparison with established prediction models in the validation set in the subgroup of patients with Child-Pugh A.

Abbreviations: CABLE: **C**RP, **a**lbumin, **b**ilirubin, **l**ymphocytes, **E**COG and **E**HS; Model$_i$: Cox PH model derived from the imputed training set; ALBI: albumin-bilirubin; PNI: prognostic nutritional index; CRAFITY: CRP and AFP in immunotherapy; EZ-ALBI: Easy-ALBI; mALBI: modified ALBI; GPS: Glasgow prognostic score; NLR: neutrophil-to-lymphocyte ratio; PLR: platelet-to-lymphocyte ratio.

TRIPOD checklist

Section/Topic	Item		Checklist Item	Page
**Title and abstract**				
Title	1	D;V	Identify the study as developing and/or validating a multivariable prediction model, the target population, and the outcome to be predicted.	1 (developme nt/validation not possible due to limitation in title length)
Abstract	2	D;V	Provide a summary of objectives, study design, setting, participants, sample size, predictors, outcome, statistical analysis, results, and conclusions.	8
**Introduction**				
Background and objectives	3a	D;V	Explain the medical context (including whether diagnostic or prognostic) and rationale for developing or validating the multivariable prediction model, including references to existing models.	10-11
	3b	D;V	Specify the objectives, including whether the study describes the development or validation of the model or both.	11
**Methods**				
Source of data	4a	D;V	Describe the study design or source of data (e.g., randomized trial, cohort, or registry data), separately for the development and validation data sets, if applicable.	12
	4b	D;V	Specify the key study dates, including start of accrual; end of accrual; and, if applicable, end of follow-up.	12
Participants	5a	D;V	Specify key elements of the study setting (e.g., primary care, secondary care, general population) including number and location of centres.	12 + Suppl. 2
	5b	D;V	Describe eligibility criteria for participants.	Suppl. 2
	5c	D;V	Give details of treatments received, if relevant.	Suppl. 2
Outcome	6a	D;V	Clearly define the outcome that is predicted by the prediction model, including how and when assessed.	Suppl. 2-3
	6b	D;V	Report any actions to blind assessment of the outcome to be predicted.	n/a
Predictors	7a	D;V	Clearly define all predictors used in developing or validating the multivariable prediction model, including how and when they were measured.	Suppl. 3
	7b	D;V	Report any actions to blind assessment of predictors for the outcome and other predictors.	n/a
Sample size	8	D;V	Explain how the study size was arrived at.	Suppl. 4
Missing data	9	D;V	Describe how missing data were handled (e.g., complete-case analysis, single imputation, multiple imputation) with details of any imputation method.	Suppl. 5-6
Statistical analysis methods	10a	D	Describe how predictors were handled in the analyses.	12-14 + Suppl. 4-6
	10b	D	Specify type of model, all model-building procedures (including any predictor selection), and method for internal validation.	12-14 + Suppl. 4-6
	10c	V	For validation, describe how the predictions were calculated.	12-14 + Suppl. 4
	10d	D;V	Specify all measures used to assess model performance and, if relevant, to compare multiple models.	13-14
	10e	V	Describe any model updating (e.g., recalibration) arising from the validation, if done.	n/a
Risk groups	11	D;V	Provide details on how risk groups were created, if done.	14
Development vs. validation	12	V	For validation, identify any differences from the development data in setting, eligibility criteria, outcome, and predictors.	12 + Suppl. 2
**Results**				
Participants	13a	D;V	Describe the flow of participants through the study, including the number of participants with and without the outcome and, if applicable, a summary of the follow-up time. A diagram may be helpful.	Fig 1
	13b	D;V	Describe the characteristics of the participants (basic demographics, clinical features, available predictors), including the number of participants with missing data for predictors and outcome.	Table 1
	13c	V	For validation, show a comparison with the development data of the distribution of important variables (demographics, predictors and outcome).	Table 1
Model development	14a	D	Specify the number of participants and outcome events in each analysis.	Fig 1
	14b	D	If done, report the unadjusted association between each candidate predictor and outcome.	Table S4
Model specification	15a	D	Present the full prediction model to allow predictions for individuals (i.e., all regression coefficients, and model intercept or baseline survival at a given time point).	Web calculator
	15b	D	Explain how to the use the prediction model.	19-20
Model performance	16	D;V	Report performance measures (with CIs) for the prediction model.	16-19
Model-updating	17	V	If done, report the results from any model updating (i.e., model specification, model performance).	n/a
**Discussion**				

Limitations	18	D;V	Discuss any limitations of the study (such as nonrepresentative sample, few events per predictor, missing data).	23
Interpretation	19a	V	For validation, discuss the results with reference to performance in the development data, and any other validation data.	21-22
	19b	D;V	Give an overall interpretation of the results, considering objectives, limitations, results from similar studies, and other relevant evidence.	24
Implications	20	D;V	Discuss the potential clinical use of the model and implications for future research.	24
**Other information**				
Supplementary information	21	D;V	Provide information about the availability of supplementary resources, such as study protocol, Web calculator, and data sets.	Suppl.
Funding	22	D;V	Give the source of funding and the role of the funders for the present study.	5

References

Author names in bold designate shared co-first authorship

[1] Scheiner B, Pomej K, Kirstein MM et al. Prognosis of patients with hepatocellular carcinoma treated with immunotherapy - development and validation of the CRAFITY score. Journal of Hepatology 2022;76(2):353–63.

[2] **Fulgenzi CAM, Cheon J,** D'Alessio A et al. Reproducible safety and efficacy of atezolizumab plus bevacizumab for HCC in clinical practice: Results of the AB-real study. European journal of cancer (Oxford, England 1990) 2022;175:204–13.

[3] Himmelsbach V, Pinter M, Scheiner B et al. Efficacy and Safety of Atezolizumab and Bevacizumab in the Real-World Treatment of Advanced Hepatocellular Carcinoma: Experience from Four Tertiary Centers. Cancers 2022;14(7):1722.

[4] **Castro T de, Jochheim LS,** Bathon M et al. Atezolizumab and bevacizumab in patients with advanced hepatocellular carcinoma with impaired liver function and prior systemic therapy: a real-world experience. Therapeutic advances in medical oncology 2022;14:17588359221080298.

[5] D’Alessio A, Fulgenzi CAM, Nishida N et al. Preliminary evidence of safety and tolerability of atezolizumab plus bevacizumab in patients with hepatocellular carcinoma and Child-Pugh A and B cirrhosis: a real-world study. Hepatology 2022.

[6] Sinner F, Pinter M, Scheiner B et al. Atezolizumab Plus Bevacizumab in Patients with Advanced and Progressing Hepatocellular Carcinoma: Retrospective Multicenter Experience. Cancers 2022;14(23).

[7] Ben Khaled N, Mörtl B, Beier D et al. Changing treatment landscape associated with improved survival in advanced hepatocellular carcinoma: a nationwide,

population-based study. European journal of cancer (Oxford, England 1990) 2023;192:113248.

[8] Johnson PJ, Berhane S, Kagebayashi C et al. Assessment of liver function in patients with hepatocellular carcinoma: a new evidence-based approach-the ALBI grade. JCO 2015;33(6):550–8.

[9] Onodera T, Goseki N, Kosaki G. Prognostic nutritional index in gastrointestinal surgery of malnourished cancer patients. Nihon Geka Gakkai zasshi 1984;85(9):1001–5.

[10] Nozoe T, Ninomiya M, Maeda T et al. Prognostic nutritional index: a tool to predict the biological aggressiveness of gastric carcinoma. Surgery today 2010;40(5):440–3.

[11] Wu YL, Fulgenzi CAM, D'Alessio A et al. Neutrophil-to-Lymphocyte and Platelet-to-Lymphocyte Ratios as Prognostic Biomarkers in Unresectable Hepatocellular Carcinoma Treated with Atezolizumab plus Bevacizumab. Cancers 2022;14(23).

[12] Kariyama K, Nouso K, Hiraoka A et al. EZ-ALBI Score for Predicting Hepatocellular Carcinoma Prognosis. Liver Cancer 2020;9(6):734–43.

[13] Hiraoka A, Kumada T, Tsuji K et al. Validation of Modified ALBI Grade for More Detailed Assessment of Hepatic Function in Hepatocellular Carcinoma Patients: A Multicenter Analysis. Liver Cancer 2019;8(2):121–9.

[14] Tada T, Kumada T, Hiraoka A et al. New prognostic system based on inflammation and liver function predicts prognosis in patients with advanced unresectable hepatocellular carcinoma treated with atezolizumab plus bevacizumab: A validation study. Cancer medicine 2023;12(6):6980–93.

[15] Åberg F, Luukkonen PK, But A et al. Development and validation of a model to predict incident chronic liver disease in the general population: The CLivD score. Journal of Hepatology 2022;77(2):302–11.
