# Supplementary material for: Evaluation of prognostic scores in patients with HCC undergoing first-line immunotherapy with atezolizumab and bevacizumab
Source: JHEP Rep. 2024 Dec 5;7(3):101295. doi: 10.1016/j.jhepr.2024.101295 (PMC11889551; doi:10.1016/j.jhepr.2024.101295)
Supplement: Multimedia component 4 [file mmc4.pdf]

Research article

JHEP|Reports

Evaluation of prognostic scores in patients with HCC undergoing first-line immunotherapy with atezolizumab and bevacizumab

Authors

Simon Johannes Gairing, Philipp Mildenberger, Jennifer Gile, …, Sumera I. Ilyas, Irene Schmidtmann, Friedrich Foerster

Correspondence

friedrich.foerster@unimedizin-mainz.de (F. Foerster).

Graphical abstract

Background & Aims

- Several prognostic scores are used to predict prognosis in patients with unresectable HCC. There is no large head-to-head validation in patients undergoing first line immunotherapy with atezolizumab and bevacizumab, and prognosis prediction for individuals is lacking
- This study aimed to develop and validate a prognostic prediction model to estimate individual prognosis and to compare it with established scores

Methods

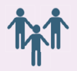

683 patients with uHCC from 24 centers in Europe and the US (training: 526, validation: 157)

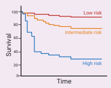

TRIPOD guidelines were used for reporting and statistical analysis. A Cox model was developed and validated. The outcome of interest was overall survival.

Results

CABLE score

Six predictors were included in the final model:

- **C**RP$_{log10}$
- **A**lbumin (smoothing spline)
- **B**ilirubin$_{log10}$
- **L**ymphocytes
- **E**COG (0/1)
- **E**HS (0/1)

Time-dependent AUC in the training cohort

Models	6 months	12 months	18 months
CABLE score	0.774	0.792	0.815
ALBI	0.719	0.715	0.74
EZ-ALBI	0.715	0.715	0.732
mALBI	0.677	0.660	0.679
PNI	0.722	0.735	0.774
CRAFITY	0.676	0.693	0.607
GPS	0.626	0.689	0.702
NLR	0.656	0.643	0.721
PLR	0.607	0.62	0.647

Patients risk-stratified in three groups according to their CABLE score (training cohort)

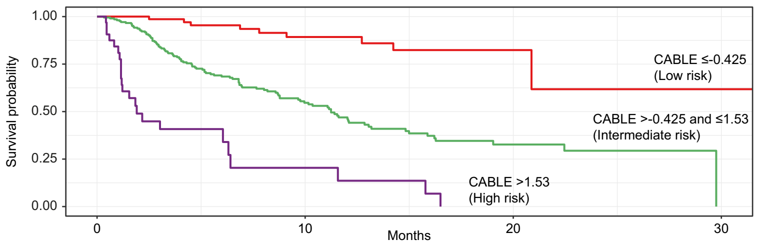

Highlights

- The prognosis of patients with unresectable HCC receiving immunotherapy with atezolizumab and bevacizumab varies greatly.
- Several scores to predict the prognosis of patients have been established but their incremental value has so far been unknown.
- We developed and validated the prognostic CABLE score that can be applied using a web-based calculator.
- We found large differences in the predictive performance of the tested models.
- In patients with Child-Pugh A, the CABLE score outperformed ALBI, EZ-ALBI, and mALBI in the first 9 months.

Impact and implications

The CABLE score allows estimation of the prognosis of patients with unresectable hepatocellular carcinoma undergoing first-line immunotherapy with atezolizumab and bevacizumab at an individual level using our web-based calculator. This feature, as well as the evaluation of the score's added benefit through an extensive comparison with other established scores, can inform clinicians on their significance and may guide clinical decision-making in the context of a malignant disease where the prognosis has become highly variable. Further large validation studies are needed to investigate the incremental value of the CABLE score compared with the ALBI score, in particular in subgroups such as patients designated as Child-Pugh A.

UNCORRECTED PROOF

https://doi.org/10.1016/j.jhepr.2024.101295
© 2024 The Author(s). Published by Elsevier B.V. on behalf of European Association for the Study of the Liver (EASL). This is an open access article under the CC BY license (http://creativecommons.org/licenses/by/4.0/).

JHEP|Reports

Evaluation of prognostic scores in patients with HCC undergoing first-line immunotherapy with atezolizumab and bevacizumab

Simon Johannes Gairing[1], Philipp Mildenberger[2], Jennifer Gile[3], Fabian Artusa[4], Bernhard Scheiner[5], Catherine Leyh[6], Sabine Lieb[7], Friedrich Sinner[8], Vincent Jörg[9], Thorben Fruendt[9], Vera Himmelsbach[10], Nada Abedin[10], Cennet Sahin[11], Katrin Böttcher[12], Jasmin Schuhbaur[13], Simon Labuhn[14], James Korolewicz[15], Claudia A.M. Fulgenzi[15], Antonio D'Alessio[15], Valentina Zanuso[16,17], Florian Hucke[18], Natascha Röhlen[19], Najib Ben Khaled[20], Eleonora Ramadori[21], Lukas Müller[22], Arndt Weinmann[1], Roman Kloeckner[23], Peter Robert Galle[1], Nguyen H. Tran[3], Sudhakar K. Venkatesh[24], Andreas Teufel[25,26], Matthias Ebert[26,27], Enrico N. De Toni[20], Dirk-Thomas Waldschmidt[21], Jens U. Marquardt[28], Dominik Bettinger[19], Markus Peck-Radosavljevic[18], Andreas Geier[29], Florian P. Reiter[29], Lorenza Rimassa[16,17], David J. Pinato[15,30], Christoph Roderburg[14], Thomas Ettrich[13], Michael Bitzer[31], Veit Scheble[31], Ursula Ehmer[12], Marie-Luise Berres[11], Fabian Finkelmeier[10], Maria Angeles Gonzalez-Carmona[32], Johann von Felden[9], Kornelius Schulze[9], Marino Venerito[8], Florian van Bömmel[7], Leonie S. Jochheim[6], Matthias Pinter[5], Raphael Mohr[4], Sumera I. Ilyas[33], Irene Schmidtmann[2], Friedrich Foerster[1,*]

JHEP Reports **2025**. vol. 7 | 1–13

Check for updates

Background & Aims: Immunotherapy with atezolizumab and bevacizumab (a + b) has improved the prognosis of patients with unresectable hepatocellular carcinoma (HCC). However, the outcome for individual patients is highly variable. This study aimed to (i) develop and validate a prognostic prediction model to estimate individual prognosis and (ii) compare it with established models.

Methods: In this multicenter retrospective study, patients with HCC undergoing first-line immunotherapy with a + b from 24 centers (Europe, USA) were included. Statistical analysis and reporting followed the TRIPOD guidelines. The primary objective was overall survival (OS). A Cox model was developed and externally validated.

Results: In total, 683 patients were included (training: 526, validation: 157). The C-reactive protein, albumin, bilirubin, lymphocytes, ECOG performance status, and extrahepatic spread (CABLE score) remained significantly associated with OS in Cox regression analysis. In the training set, the CABLE score had a higher discriminatory accuracy relative to ALBI, EZ-ALBI, mALBI, CRAFITY, PNI, NLR, PLR, and GPS (time-dependent AUC 0.79 and C-index 0.75 (95% CI 0.71–0.78) at 12 months). In the external validation set, the discriminatory performance of the CABLE score was comparable to ALBI, EZ-ALBI, and mALBI, but on average higher than PNI, CRAFITY, NLR, PLR, and GPS. In patients with Child-Pugh A, the CABLE score outperformed ALBI, EZ-ALBI, and mALBI in the first 9 months. We provide a web-based calculator for the CABLE score to allow estimation of individual prognosis for these patients (http://shiny.imbei.uni-mainz.de:3838/CABLE_Score/).

Conclusions: The CABLE score shows good discriminatory performance in assessing the individual prognosis of patients undergoing first-line immunotherapy with a + b. Further validation studies are needed to investigate its performance compared with the ALBI score, in particular in subgroup analysis.

© 2024 The Author(s). Published by Elsevier B.V. on behalf of European Association for the Study of the Liver (EASL). This is an open access article under the CC BY license (http://creativecommons.org/licenses/by/4.0/).

Introduction

Liver cancer is the third leading cause of cancer-associated deaths worldwide and its incidence is predicted to continue to rise.[1,2] In particular, unresectable hepatocellular carcinoma (uHCC) is associated with poor prognosis. Immunotherapy with atezolizumab (a; anti-programmed death-ligand 1 [PD-L1] antibody) and bevacizumab (b; anti-vascular endothelial growth factor [VEGF] antibody) has significantly improved prognosis of patients with unresectable HCC relative to sorafenib yielding a median overall survival (OS) of 19.2 months.[3–5] Thus, a + b is currently one of the recommended first-line (1L) systemic treatments for patients with uHCC.[6,7]

In addition to tumor-related characteristics, the prognosis of this population is highly dependent on several additional factors arising from the underlying chronic liver disease. In this context, systemic inflammation plays a key role by fueling the development of HCC irrespective of its underlying etiology.[8] In addition, various biomarkers reflecting the stage of systemic inflammation including C-reactive protein (CRP), lymphocytes or the negative acute-phase protein albumin are associated with outcome in HCC.[8]

* Corresponding author. Address: Department of Internal Medicine I, University Medical Center of the Johannes Gutenberg University Mainz, Langenbeckstr. 1, 55131 Mainz, Germany. Tel.: +49 6131 17 6077.
E-mail address: friedrich.foerster@unimedizin-mainz.de (F. Foerster).
https://doi.org/10.1016/j.jhepr.2024.101295

ELSEVIER

EASL The Home of Hepatology

Currently, there are several prognostic scoring systems reflecting liver function, systemic inflammation or immunonutritive status.[9–12] In the past, the Child-Pugh score has been used to assess liver function in patients with HCC. However, the albumin–bilirubin (ALBI) score which contains only two predictors (albumin and bilirubin) is increasingly replacing the Child-Pugh score because of its complete objectivity and at least similar discriminative performance in patients with HCC[9,13,14] and is increasingly being used in the setting of clinical trials evaluating treatments for HCC.[9,15,16] Correspondingly, *post hoc* analyses of phase 3 trials testing a + b,[17] STRIDE,[18] lenvatinib,[19] cabozantinib,[20] Click or tap here to enter text. and ramucirumab[21] have demonstrated the prognostic value of the ALBI score (for a comprehensive review of the body of evidence for the role of ALBI in HCC refer to Demirtas *et al.*[22]). In addition, further modifications of the ALBI score such as easy-ALBI (EZ-ALBI)[23] Click or tap here to enter text. and modified ALBI[24,25] have been suggested to improve its prognostic ability and applicability. In the context of systemic treatment of uHCC with a + b, several other prognostic scores have been proposed or evaluated previously: (i) The CRAFITY (CRP and AFP in immunotherapy) score which combines CRP and alpha-fetoprotein (AFP).[10] (ii) The Glasgow Prognostic Score (GPS) which combines albumin with CRP, and which was also shown to be associated with OS.[26,27] (iii) The Prognostic Nutritional Index (PNI) which combines albumin and absolute lymphocyte counts which has been demonstrated to be an independent prognostic factor for OS and progression-free survival (PFS).[28–30] (iv) Scores reflecting selected cellular components of the peripheral blood and thereby mirroring the extent of systemic inflammation, namely, neutrophil-to-lymphocyte ratio (NLR) and platelet-to-lymphocyte ratio (PLR), which have been shown to be associated with OS and PFS.[11,31–35]

However, the incremental value of these prediction models is unknown, and it is unclear which model serves the purpose of prognosis estimation best. To address this knowledge gap, this study aimed to evaluate the prognostic value of all these candidate predictors and to integrate them into one prognostic prediction model that allows accurate estimation of the individual prognosis of patients with HCC undergoing 1L immunotherapy with a + b.

Patients and methods

This study and manuscript are based on the *Transparent Reporting of a multivariable prediction model for Individual Prognosis Or Diagnosis* (TRIPOD) statement.[36,37] The methodological approach was based on that of Åberg *et al.*[38] (CLivD score).

Study design and patients

This retrospective multicenter study included patients from a total of 24 centers in Germany (DE), Austria (AT), Italy (IT), the UK, and the USA. The data set was split into a training set (center 1–12) and a validation set (center 13–24) based on the time of their data reporting. Immunotherapy with a + b was initiated between March 2018 and November 2022. Data were received until December 2022. Further details including the outcome of interest, candidate predictors, response assessment, sample size, and calculation of scores are summarized in the Supplementary methods.

Ethics

This study was approved by the ethics committee of the Medical Association of Rhineland Palatinate, Mainz, Germany (permit number 2022-16779) and conducted according to the ethical guidelines of the 1975 Declaration of Helsinki (6th revision, 2008).

Statistical analysis

Details on descriptive statistics, modification of candidate predictors, and handling of missing data are given in the Supplementary methods.

Model building

The model development consisted of two parts. In the first part, a model was fitted on the imputed data with variables having ≤10% missingness. In the second part, we examined whether the variables with >10% missingness could improve prediction in the complete cases subset (see section *Missing data*).

In the first part, a preselection of variables was made using univariable Cox proportional hazard models with center as random effect to account for different case mixes across participating centers. Continuous variables were fitted with cubic regression splines to account for potential non-linear effects. Variables with $p < 0.1$ were considered for the multivariable model. We then imputed 10 sets with predictive mean matching, using the R-package mice and fitted a model with only continuous terms on each imputation set. Shrinkage smoothers were used for variable selection as described in Marra and Wood.[39] Knots were placed explicitly to allow for later pooling of the models. We considered three variable sets of continuous variables as candidates with variable inclusion frequency 10%, 50%, and 100%, respectively. For categorical variables, we used forward selection on each imputation, which similarly resulted in three candidate sets of categorical variables. We combined continuous and categorical variable sets to get 3 × 3 = 9 candidate models. Those candidate models were compared with repeated cross validation with 10 repetitions and five folds. This led to 50 imputations because the 'hold-out-fold' was ignored in the imputation procedure to prevent contamination. The model with the best time-dependent AUC was then selected.

In the second part, the variables with missingness of >10% were then added to the resulting model from the first part. A backward–forward elimination on the subset of complete cases resulted in the final model.

Model performance measures

Discrimination. The model performance in terms of discrimination was analyzed by the time-dependent AUC using the R-package timeROC and Uno's C with bootstrapped confidence intervals.[40,41]

Calibration. To analyze the calibration of the prediction model, patients in the training and validation set were divided into quintiles according to their prognosis in the CABLE score. Subsequently, the agreement between the observed and

predicted survival was visually compared using the Kaplan–Meier curve within each quintile.[42]

Risk groups

Risk groups were identified by dividing the patients into three groups according to their prognostic score (low risk: lowest 25%, intermediate risk: 25–90%, high risk: highest 10%).

External validation

To externally validate the newly developed model, data from centers 13–24 were used. Here, only patients with complete data on the variables included in the models and the established scores were used (n = 157, outcome events = 60, Fig. 1). Again, discrimination was analyzed by comparing the time-dependent AUC and Uno's C.

Results

Study cohort

The training cohort included 526 patients, the external validation cohort 157 patients. Fig. 1 displays the composition of the training and validation cohort and the number of outcome

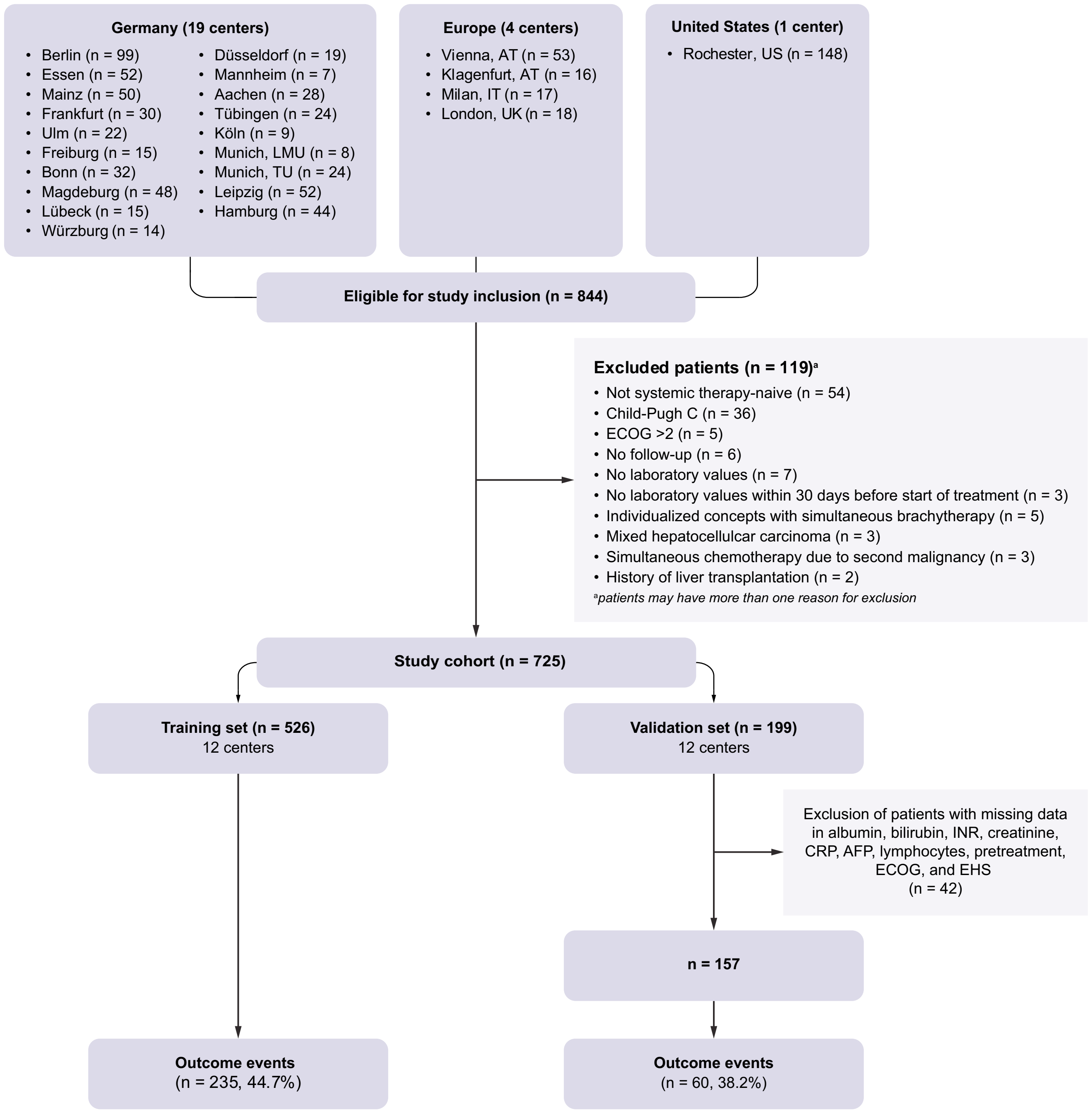

Fig. 1. Flow chart of patients eligible for study inclusion. AT, Austria; ECOG, Eastern Cooperative Oncology Group; GB, Great Britain; IT, Italy; LMU, Ludwig Maximilian University of Munich; TU, Technical University of Munich; US, United States.

events, respectively. Before data analysis, we decided to use centers 1–12 as the training set, and centers 13–24 as the validation set. Table 1 summarizes the baseline characteristics of the study participants for both cohorts including the number of missing values for each candidate predictor. Compared with the training set, the validation cohort had significantly lower Eastern Cooperative Oncology Group (ECOG) performance stages, a lower frequency of pretreatment, different etiology composition (more alcohol-associated, less viral), lower international normalized ratio (INR) levels, and no brachytherapy. No outcome event data were missing. The median follow-up time was 12.6 months (95% CI: 11.7–14.0) for the training set, and 11.9 months (95% CI: 10.9–14.8) for the validation set (reverse Kaplan–Meier method). In total, two patients were censored because of liver transplantation during follow-up. The reasons for discontinuation of a + b are detailed in Table S2.

Overall survival and tumor response

Median OS was 13.7 months (95% CI: 12.1–15.8) for the training cohort, and 16.0 months (95% CI: 13.5, not reached) for the validation cohort. In the subgroup of patients with Child-Pugh A and with an ECOG of 0–1, median OS was 20.9 months (95% CI: 15.7, not reached) in the training cohort and 19.0 months (95% CI: 14.9, not reached) in the validation cohort. In total, 343 patients in the training cohort (65.2%), and 142 patients (90.4%) in the validation cohort had available follow-up imaging data. The overall response rate (ORR) was 31.8% in the training, and 31.0% in the validation cohort. The disease control rate (DCR) was 71.7% in the training, and 69.7% in the validation cohort. Detailed response data are given in Table S3.

Model building

The stepwise model building process is described in detail in the Patients and methods section. First, candidate predictors were preselected via univariable models (Table S4). In the imputed training set (n = 526), seven predictors including albumin (smoothing spline), $creatinine_{log10}$ (smoothing spline), $bilirubin_{log10}$, INR_{log10}, ECOG (0/1/2), extrahepatic spread (EHS; no/yes), and pretreatment (no/yes) remained in the model after the variable selection procedure (Model with imputed data: $Model_i$) Fig. S1A shows the smoothing splines of albumin and $creatinine_{log10}$. Table S5 displays the adjusted log(HR) for $Model_i$.

Next, the training set was trimmed to complete cases for the variables included in the $Model_i$ and CRP, lymphocytes, and monocytes (n = 320, outcome events: 136). Baseline characteristics for this data set are given in Table S6. Here, six predictors remained in the model including albumin (smoothing spline), $bilirubin_{log10}$, lymphocytes, CRP_{log10}, ECOG (0/1 [whereby the value 1 includes ECOG 1 and 2]), and EHS (no/yes). This score was named CABLE score (CRP, Albumin, Bilirubin, Lymphocytes, ECOG, and EHS). Fig. S1B displays the non-linear predictor-outcome relation of albumin in the CABLE score. Table 2 displays the adjusted log(HR) for the CABLE score. Table S7 shows the variables included in the CABLE score compared with other prediction models.

Internal validation

For the internal and external validation, only individuals with complete data for the tested predictors were included (complete-case analysis). Fig. 2 displays the time-dependent AUC of the CABLE score compared to $Model_i$, ALBI (continuous and graded), EZ-ALBI, mALBI, CRAFITY, PNI, NLR, PLR, and GPS. The CABLE score continuously had the highest AUC values (0.77–0.82). $Model_i$ yielded the second highest AUC values (0.75–0.79). PNI, ALBI, and EZ-ALBI had AUC values >0.7. CRAFITY, mALBI, ALBI graded, and PLR had AUC values <0.7. Table S8 shows the AUC for all scores at 6, 12, and 18 months. Table 3 shows the respective Uno's C indices for 6, 12, and 18 months. Here, the CABLE score showed the highest C-index at all time points. Additionally, only the CABLE score and $Model_i$ yielded values >0.7. PNI, ALBI, and EZ-ALBI had C indices between 0.68 and 0.69. All other scores had C indices between 0.59 and 0.66.

External validation

Fig. 3 displays the time-dependent AUC of the CABLE score compared to $Model_i$, ALBI (continuous and graded), EZ-ALBI, mALBI, CRAFITY, PNI, NLR, PLR, and GPS in the validation cohort. The AUCs for 6, 12, and 18 months are displayed in Table S9. Compared with the training set, most models showed a higher time-dependent AUC on average at 6 months. At 6 months, the CABLE score, $Model_i$, ALBI, and EZ-ALBI had the highest AUC values (AUC 0.84–0.85). mALBI and GPS had AUC values >0.8, while PNI, ALBI graded, and CRAFITY showed AUC values between 0.75 and 0.76. NLR and PLR had the lowest performance with AUC values <0.6. At 12 months, ALBI, EZ-ALBI, PNI, and mALBI had AUC values between 0.73 and 0.74, the CABLE score and $Model_i$ values of 0.71. ALBI graded, GPS, CRAFITY, NLR, and PLR had values <0.7. At 18 months, the CABLE score, ALBI, EZ-ALBI, mALBI, and PNI had the highest AUC values (0.75–0.76), while ALBI graded, $Model_i$, and CRAFITY had AUC values between 0.71–0.73. GPS, NLR, and PLR had values <0.7. Uno's C indices for 6, 12, and 18 months are given in Table S10. The CABLE score, $Model_i$, ALBI, EZ-ALBI, and mALBI had C indices >0.7 at all time points.

Risk groups

For the CABLE score, risk groups were created by stratifying the training and validation set according to their prognostic score into three groups (low risk: lowest 25% (≤0.425), intermediate risk: 25–90% (>-0.425 and ≤1.53), high risk: highest 10% (>1.53)). Kaplan–Meier curves for the CABLE score for the training and validation set are shown in Fig. 4. The groups identified patients with clearly different prognosis yielding a median overall survival of *not reached* (95% CI: 20.88, *not reached*; low risk), 11.24 months (95% CI 9.67–14.83; intermediate risk), and 1.91 months (95% CI 1.18–11.57; high risk) in the training set, and 21.34 months (95% CI 18.97, *not reached*; low risk), 12.92 months (95% CI 9.37;18.05; intermediate risk), and 5.62 months (95% CI 3.32, NA; high risk) in the validation set.

Calibration

Calibration plots for the CABLE score are shown in Fig. S2. The CABLE score showed a very good agreement in Q1–Q5 in the training set. In the validation set, the agreement in Q5 and Q4 was high, medium in Q2 and Q3, and low in Q1.

Table 1. Demographics and baseline characteristics of the study cohort.

	Training set (n = 526)	Validation set (n = 157)	*p* values
Age (years)			
Median (Min, Max)	67.0 (25.0, 89.5)	69.0 (29.0, 85.0)	0.1
Sex			
Male	427 (81.2%)	127 (80.9%)	1
Female	99 (18.8%)	30 (19.1%)	
ECOG			
0	239 (45.4%)	92 (58.6%)	0.009
1	251 (47.7%)	60 (38.2%)	
2	36 (6.8%)	5 (3.2%)	
Cirrhosis			
No	133 (25.3%)	41 (26.3%)	0.9
Yes	393 (74.7%)	115 (73.7%)	
Missing	0 (0%)	1 (0.6%)	
Etiology			
Unknown/no liver disease	91 (17.3%)	34 (21.7%)	<0.001
HBV	55 (10.5%)	7 (4.5%)	
HCV	93 (17.7%)	22 (14.0%)	
Harmful alcohol use	128 (24.3%)	62 (39.5%)	
MASLD/MASH	89 (16.9%)	15 (9.6%)	
Mixed (viral and non-viral)	34 (6.5%)	6 (3.8%)	
Mixed (only non-viral)	17 (3.2%)	3 (1.9%)	
Mixed (only viral)	1 (0.2%)	2 (1.3%)	
Other	18 (3.4%)	6 (3.8%)	
BCLC			
A	8 (1.5%)	0 (0%)	0.2
B	133 (25.3%)	47 (29.9%)	
C	385 (73.2%)	110 (70.1%)	
Pretreatment			
No	231 (43.9%)	87 (55.4%)	0.01
Yes	295 (56.1%)	70 (44.6%)	
History of surgery			
No	419 (79.7%)	131 (83.4%)	0.3
Yes	107 (20.3%)	26 (16.6%)	
History of ablation			
No	469 (89.2%)	141 (89.8%)	0.9
Yes	57 (10.8%)	16 (10.2%)	
History of TACE/TAE			
No	358 (68.1%)	115 (73.2%)	0.3
yes	168 (31.9%)	42 (26.8%)	
History of SIRT/TARE			
No	475 (90.3%)	143 (91.1%)	0.9
Yes	51 (9.7%)	14 (8.9%)	
History of radiation/SBRT			
No	517 (98.3%)	157 (100%)	0.2
Yes	9 (1.7%)	0 (0%)	
History of brachytherapy			
No	502 (95.4%)	157 (100%)	0.01
Yes	24 (4.6%)	0 (0%)	
Macrovascular invasion (MVI)			
No	295 (56.1%)	97 (61.8%)	0.2
Yes	231 (43.9%)	60 (38.2%)	
Extrahepatic spread (EHS)			
No	311 (59.1%)	105 (66.9%)	0.1
Yes	215 (40.9%)	52 (33.1%)	
Child-Pugh			
A	384 (73.4%)	114 (72.6%)	0.9
B	139 (26.6%)	43 (27.4%)	
Missing	3 (0.6%)	0 (0%)	
ALBI score			
Median (Min, Max)	-2.42 (-3.70, -0.733)	-2.40 (-3.46, -0.677)	0.7
Missing	21 (4.0%)	0 (0%)	
ALBI grade			
1	194 (38.4%)	65 (41.4%)	0.08
2	287 (56.8%)	78 (49.7%)	
3	24 (4.8%)	14 (8.9%)	
Missing	21 (4.0%)	0 (0%)	

(*continued on next page*)

Table 1. (*continued*)

	Training set (n = 526)	Validation set (n = 157)	*p* values
mALBI grade			
1	194 (38.4%)	65 (41.4%)	0.2
2a	102 (20.2%)	27 (17.2%)	
2b	185 (36.6%)	51 (32.5%)	
3	24 (4.8%)	14 (8.9%)	
Missing	21 (4.0%)	0 (0%)	
EZ-ALBI			
(Min, Max)	-32.9 (-45.2, -14.6)	-32.5 (-42.7, -14.6)	0.4
Missing	21 (4.0%)	0 (0%)	
CRAFITY score			
Low	104 (29.7%)	51 (32.5%)	0.7
Intermediate	151 (43.1%)	62 (39.5%)	
High	95 (27.1%)	44 (28.0%)	
Missing	176 (33.5%)	0 (0%)	
GPS			
GPS 0	130 (37.4%)	64 (40.8%)	0.8
GPS 1	133 (38.2%)	56 (35.7%)	
GPS 2	85 (24.4%)	37 (23.6%)	
Missing	178 (33.8%)	0 (0%)	
PNI			
Median (Min, Max)	43.2 (23.0, 63.1)	42.9 (20.8, 58.0)	0.6
Missing	74 (14.1%)	0 (0%)	
NLR			
Median (min, max)	3.62 (0.337, 117)	3.87 (0.834, 21.8)	0.7
Missing	62 (11.8%)	0 (0%)	
PLR			
Median (min, max)	151 (33.3, 3300)	152 (25.8, 852)	0.5
Missing	63 (12.0%)	0 (0%)	
Bilirubin (mg/dl)			
Median (min, max)	0.800 (0.160, 12.2)	0.748 (0.170, 6.31)	0.4
Missing	1 (0.2%)	0 (0%)	
Albumin (g/dl)			
Median (min, max)	3.80 (1.70, 5.16)	3.70 (1.73, 4.80)	0.4
Missing	21 (4.0%)	0 (0%)	
INR			
Median (min, max)	1.15 (0.890, 3.90)	1.10 (0.900, 4.30)	0.01
Missing	6 (1.1%)	0 (0%)	
Creatinine (mg/dl)			
Median (min, max)	0.870 (0.386, 8.00)	0.890 (0.380, 5.40)	0.4
Missing	2 (0.4%)	0 (0%)	
CRP (mg/l)			
Median (min, max)	10.2 (0.200, 299)	10.0 (0.100, 173)	0.6
Missing	172 (32.7%)	0 (0%)	
AFP (ng/ml)			
Median (min, max)	56.1 (1.00, 2,070,000)	44.1 (1.00, 91,000)	0.7
Missing	11 (2.1%)	0 (0%)	
Platelets per nl			
Median (min, max)	164 (29.0, 760)	170 (35.0, 483)	0.5
Missing	3 (0.6%)	0 (0%)	
Neutrophils per nl			
Median (min, max)	4.10 (0.550, 21.0)	4.10 (0.730, 55.7)	1
Missing	47 (8.9%)	0 (0%)	
Lymphocytes per nl			
Median (min, max)	1.09 (0.0700, 3.59)	1.13 (0.210, 3.26)	1
Missing	62 (11.8%)	0 (0%)	
Monocytes per nl			
Median (min, max)	0.600 (0.0490, 2.29)	0.570 (0.170, 2.10)	0.6
Missing	63 (12.0%)	0 (0%)	

Continuous data are given as median and range (min, max), categorial data as frequency with percentage. Percentages refer to patients with available data. A X^2 test was applied for categorial data, a Mann–Whitney *U* test or Student's *t* test for continuous data (depending on data distribution). Patients may have received more than one category of pretreatment. AFP, alpha-fetoprotein; ALBI, albumin–bilirubin; BCLC, Barcelona Clinic Liver Cancer; CRAFITY, CRP, and AFP in immunotherapy; CRP, C-reactive protein; ECOG, Eastern Cooperative Oncology Group; EZ-ALBI, easy-ALBI; GPS, Glasgow Prognostic Score; HCC, hepatocellular carcinoma; INR, international normalized ratio; MASH, metabolic dysfunction-associated steatohepatitis; mALBI, modified ALBI; MASLD, metabolic dysfunction-associated steatotic liver disease; NLR, neutrophil-to-lymphocyte ratio, PLR, platelet-to-lymphocyte ratio; PNI, prognostic nutritional index; SBRT, stereotactic body radiation therapy; SIRT, selective internal radiation therapy; TACE, transarterial chemo-embolization; TAE, transarterial embolization; TARE, transarterial radioembolization.

Table 2. Adjusted multivariable Cox model of the CABLE score.

Variables	Beta*	95% CI	*p* values
CRP_{log10}	0.55	0.20–0.90	0.002
s(Albumin)			<0.001
$Bilirubin_{log10}$	1.1	0.49–1.7	<0.001
Lymphocytes	-0.61	-0.98 to -0.25	<0.001
ECOG			
0	—	—	
1	0.60	0.21–1.0	0.003
EHS			
No	—	—	
Yes	0.40	0.03–0.77	0.033

Adjusted multivariable Cox model.
CRP, C-reactive protein; ECOG, Eastern Cooperative Oncology Group; EHS, extrahepatic spread; s, smoothing spline.
*log(HR).

Subgroup analysis

Next, we analyzed the performance of the CABLE score in relevant subgroups in the validation cohort (Table S11). For this purpose, we built univariable Cox regression models for each subgroup, using the score as the sole predictor. A higher HR indicates better relative performance. Here, the CABLE score performed well in all subgroups with the exception of the group of patients with Child-Pugh B.

Consequently, we analyzed the time-dependent AUC in the subgroup of patients with Child-Pugh A. In the training cohort, the CABLE score had the highest AUC over the entire observation period (Fig. S3). Corresponding Uno's C indices are shown in Table S12. In the validation cohort, the CABLE score mostly showed higher AUCs than the ALBI score during the first 9 months after the start of a + b (Fig. S4). From 9 months onwards, CABLE and ALBI had almost the same AUC values. Uno's C indices for 3, 6, 12, and 18 months for the CABLE score were 0.84, 0.74, 0.67, and 0.67 compared with 0.80, 0.66, 0.67, and 0.68 for the ALBI score (Table 4). EZ-ALBI and modified ALBI (mALBI) showed similar results as the ALBI score. PNI had similar results as the ALBI score in the first 5 months but showed better AUC values in the later observation period and even higher AUC values than the CABLE score from around month 8. CRAFITY showed similar results compared to the CABLE score in the first 5 months, showed a better AUC after 6 months, but had continuously lower AUC values from month 8 onwards. GPS, NLR, and PLR had continuously lower AUC values than the CABLE score (Table 4).

Prediction of ORR and DCR

Next, we tested whether the CABLE score and the other models tested are able to predict response to treatment with a + b. Table S13 shows the AUC of all models in the validation data set. In summary, all models had AUCs <0.7 and thus were not useful to predict either ORR or DCR. Table S14 shows the association of variables with ORR in terms of the explained deviance. Here, ECOG performance status, MVI, platelets, and neutrophils had a significant association with ORR. Etiology was not associated with ORR. In addition, we tried to fit new models aiming to predict response to a + b (data not shown), but given their very poor predictive performance, we did not validate these models.

Online calculator

To make the CABLE score available and usable as a tool for prognosis assessment of patients with uHCC receiving a + b, we have developed an online calculator (http://shiny.imbei.uni-mainz.de:3838/CABLE_Score/). Here, the CABLE score is calculated and the survival probability at 6, 12, and 18 months

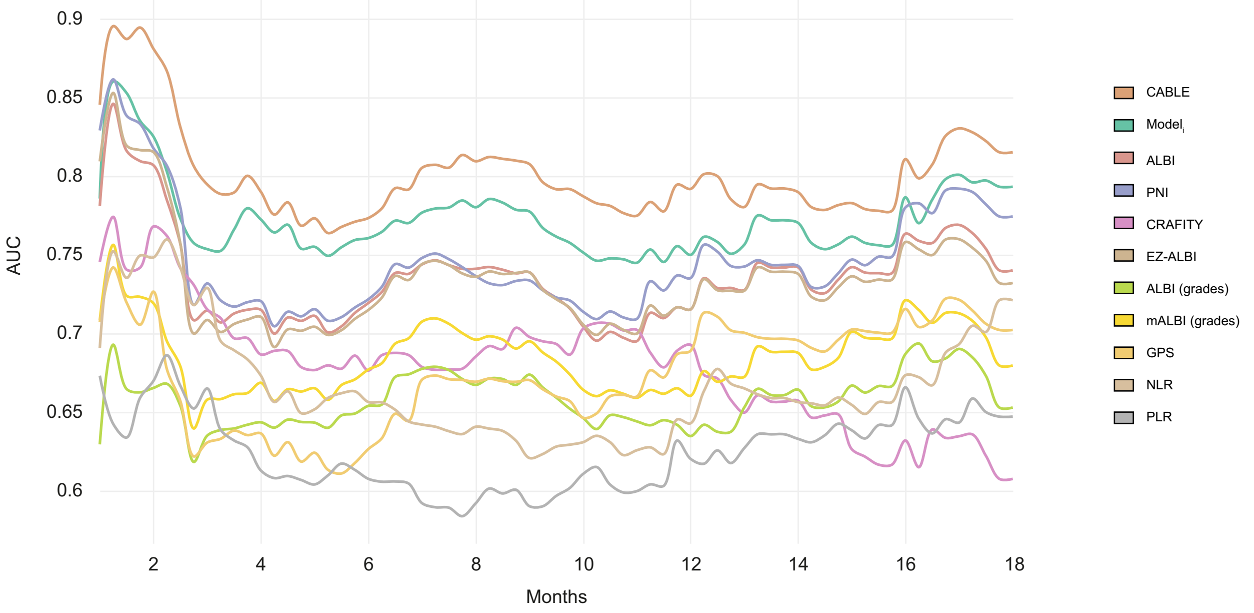

Fig. 2. Time-dependent AUC of the CABLE score and Model$_i$ in comparison with established prediction models in the training set. ALBI, albumin–bilirubin; CABLE, CRP, albumin, bilirubin, lymphocytes, ECOG, and EHS; CRAFITY, CRP and AFP in immunotherapy; EZ-ALBI, easy-ALBI; GPS, Glasgow Prognostic Score; mALBI, modified ALBI; Model$_i$, Cox PH model derived from the imputed training set; NLR, neutrophil-to-lymphocyte ratio; PLR, platelet-to-lymphocyte ratio; PNI, Prognostic Nutritional Index.

Table 3. Uno's C indices with bootstrapped confidence intervals for the training set.

Model	6 months	12 months	18 months
CABLE score	0.748 (0.692–0.804)	0.745 (0.709–0.781)	0.743 (0.707–0.78)
$Model_i$	0.731 (0.681–0.782)	0.714 (0.666–0.762)	0.717 (0.674–0.76)
ALBI	0.692 (0.628–0.756)	0.681 (0.636–0.725)	0.689 (0.639–0.739)
ALBI grade	0.626 (0.583–0.67)	0.62 (0.582–0.659)	0.627 (0.588–0.665)
mALBI grade	0.65 (0.597–0.703)	0.638 (0.585–0.692)	0.65 (0.602–0.698)
EZ-ALBI	0.686 (0.634–0.739)	0.677 (0.635–0.719)	0.684 (0.64–0.728)
PNI	0.693 (0.636–0.751)	0.689 (0.636–0.741)	0.692 (0.646–0.739)
CRAFITY	0.662 (0.607–0.717)	0.66 (0.611–0.71)	0.633 (0.588–0.678)
GPS	0.607 (0.553–0.661)	0.627 (0.584–0.671)	0.63 (0.591–0.668)
NLR	0.643 (0.575–0.71)	0.62 (0.57–0.67)	0.623 (0.568–0.678)
PLR	0.594 (0.531–0.656)	0.589 (0.532–0.647)	0.588 (0.545–0.63)

ALBI, albumin–bilirubin; CABLE, CRP, albumin, bilirubin, lymphocytes, ECOG, and EHS; CRAFITY, CRP and AFP in immunotherapy; EZ-ALBI, easy-ALBI; GPS, Glasgow prognostic score; mALBI, modified ALBI; $Model_i$, Cox PH (proportional hazards) model derived from the imputed training set; NLR, neutrophil-to-lymphocyte ratio; PLR, platelet-to-lymphocyte ratio; PNI, prognostic nutritional index.

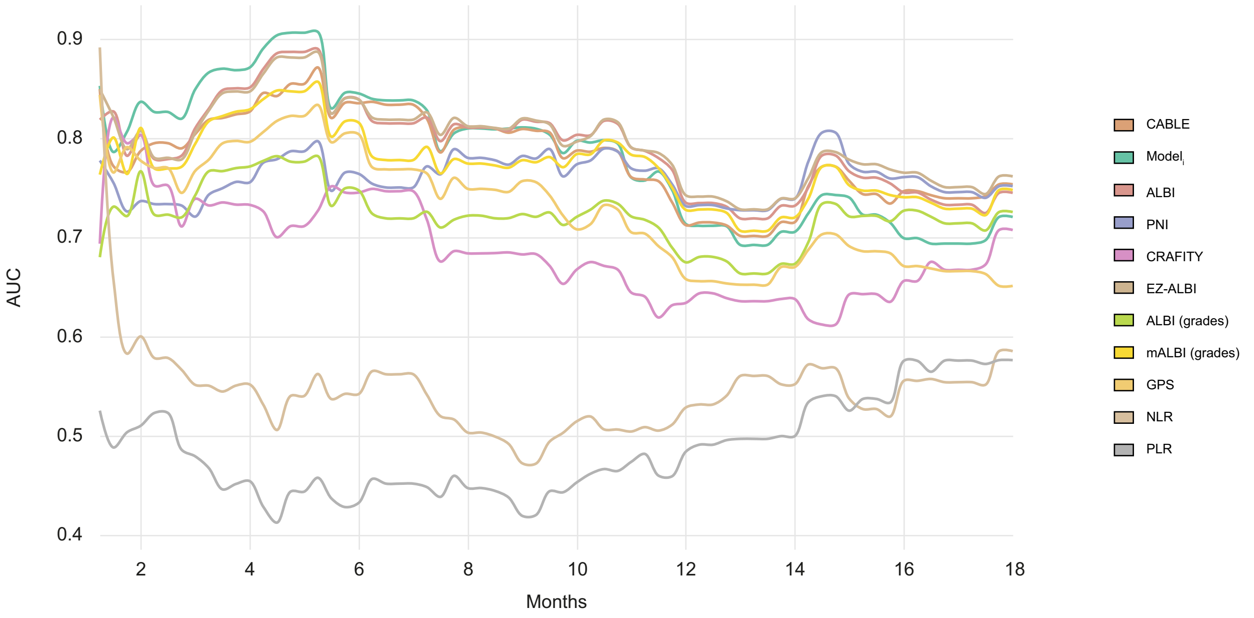

Fig. 3. Time-dependent AUC of the CABLE score and $Model_i$ compared to other prediction models in the validation cohort. ALBI, albumin–bilirubin; CABLE, CRP, albumin, bilirubin, lymphocytes, ECOG, and EHS; CRAFITY, CRP and AFP in immunotherapy; EZ-ALBI, easy-ALBI; GPS, Glasgow Prognostic Score; mALBI, modified ALBI; $Model_i$, Cox PH model derived from the imputed training set; NLR, neutrophil-to-lymphocyte ratio; PLR, platelet-to-lymphocyte ratio. PNI, Prognostic Nutritional Index.

is given. In addition, the R model for calculating the CABLE score is available for download from the app. The risk formula is not provided in the manuscript as it is not possible to include the non-linear terms here.

Discussion

In this large multicenter study, we evaluated and compared the prognostic value of several clinical variables and established scores in patients with uHCC undergoing 1L immunotherapy with a + b. We developed and validated the CABLE score that allows prediction of the individual prognosis of these patients using the provided web-calculator. In the training set, the CABLE score outperformed all other scores. In the external validation set, the CABLE score had superior discriminatory performance compared with PNI, CRAFITY, NLR, PLR, and GPS and similar performance to ALBI, EZ-ALBI, and mALBI.

Although the CABLE score did not outperform ALBI, EZ-ALBI, and mALBI in the validation cohort, we believe that this study provides valuable insights for the following reasons. First, to our knowledge, we have performed the first statistically sound and thorough head-to-head validation of the currently used scores in a large, multicenter setting of a homogenous cohort including only patients with uHCC undergoing 1L immunotherapy with a + b. Here, we found large differences in the predictive performance of currently used scores, which is of great interest for clinicians using these scores in daily clinical practice. Second, all of the extracted patient- and tumor-related characteristics and laboratory values have previously been associated with outcome of patients with HCC and are part of several scoring systems. However, the incremental value of the variables used in these prediction models in terms of improving the predictive accuracy was unknown and the rationale for this study. Our study highlights the fact that adding variables which are highly associated with the outcome to a

A

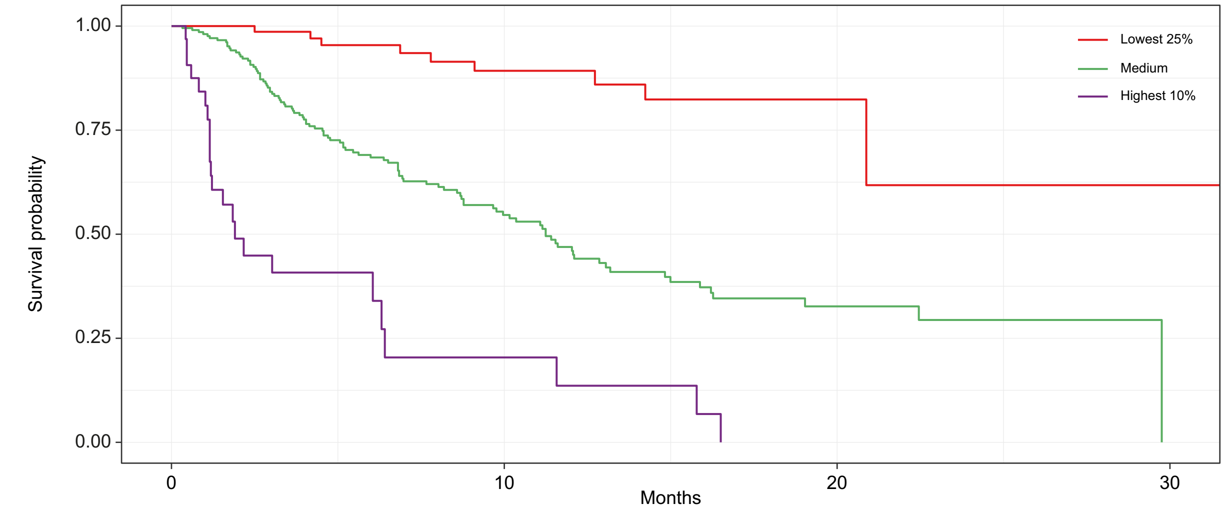

N° at risk				
(-Inf,-0.425]	80 (0)	37 (6)	6 (8)	1 (9)
(-0.425,1.53]	208 (0)	68 (81)	15 (102)	0 (104)
(1.53, Inf]	32 (0)	3 (20)	0 (23)	0 (23)

B

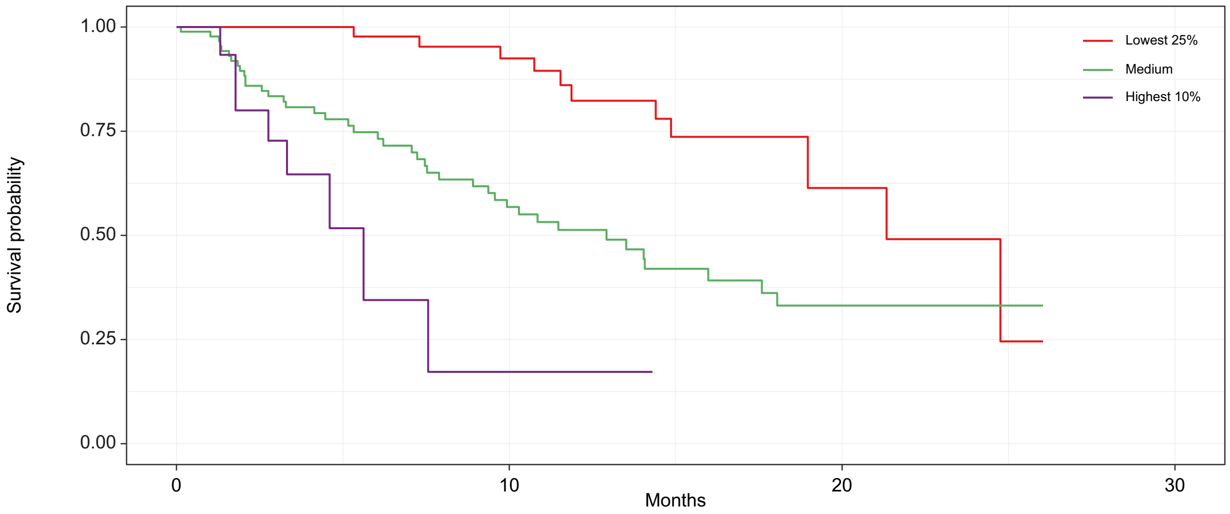

N° at risk				
(-Inf,-0.425]	51 (0)	32 (3)	5 (9)	0 (11)
(-0.425,1.53]	90 (0)	34 (31)	9 (41)	0 (41)
(1.53, Inf]	16 (0)	1 (8)	0 (8)	0 (8)

Fig. 4. Kaplan–Meier curves of patients risk-stratified into three groups according to their CABLE score. In the (A) training and (B) validation set. The lower the CABLE score, the higher the survival probability. CABLE, CRP, albumin, bilirubin, lymphocytes, ECOG, and EHS.

prediction model does not necessarily enhance its predictive performance. One explanation could be that our validation data set was too small or biased (see below). Another, and in our opinion more likely, explanation could be, that some of the information of the added variables is already captured to some extent by albumin and bilirubin (*e.g.* albumin is a negative acute-phase protein and could reflect changes in CRP levels). Consequently, other variables need to be investigated to improve the predictive accuracy of these models that capture completely independent information about patient prognosis (*e.g.* molecular markers or frailty). Third, in subgroup analyses including only patients with Child-Pugh A, the CABLE score outperformed ALBI, EZ-ALBI, and mALBI in the first 9 months. This could be because liver function reflected by albumin and bilirubin may be less relevant in patients with Child-Pugh A and that other variables such as CRP or lymphocytes may have a greater impact on the outcome in this subgroup. Fourth, we have launched a web-calculator which allows to estimate prognosis of patients on an individual level attributing them a survival probability value at 6, 12, and 18 months.

The CABLE score includes six variables which have exhibited the highest predictive performance in this cohort and

Table 4. Uno's C indices for the validation set in the subgroup of patients with Child-Pugh A with bootstrapped confidence intervals.

Model	3 months	6 months	12 months	18 months
CABLE score	0.839 (0.673–1.004)	0.739 (0.571–0.908)	0.668 (0.566–0.77)	0.671 (0.575–0.767)
$Model_i$	0.86 (0.618–1.103)	0.667 (0.406–0.928)	0.641 (0.524–0.758)	0.643 (0.528–0.757)
ALBI	0.803 (0.61–0.995)	0.664 (0.457–0.871)	0.667 (0.547–0.787)	0.676 (0.579–0.773)
EZ-ALBI	0.79 (0.612–0.969)	0.669 (0.459–0.879)	0.675 (0.567–0.783)	0.684 (0.587–0.781)
ALBI grade	0.724 (0.563–0.886)	0.63 (0.45–0.811)	0.615 (0.519–0.711)	0.632 (0.544–0.72)
mALBI grade	0.779 (0.592–0.966)	0.679 (0.489–0.868)	0.656 (0.557–0.754)	0.66 (0.568–0.753)
PNI	0.801 (0.63–0.972)	0.69 (0.501–0.879)	0.709 (0.608–0.81)	0.714 (0.623–0.805)
CRAFITY	0.836 (0.59–1.081)	0.859 (0.713–1.006)	0.613 (0.474–0.751)	0.618 (0.496–0.74)
GPS	0.78 (0.525–1.035)	0.725 (0.565–0.885)	0.626 (0.542–0.709)	0.611 (0.548–0.674)
NLR	0.627 (0.342–0.912)	0.578 (0.383–0.774)	0.564 (0.383–0.745)	0.565 (0.384–0.747)
PLR	0.454 (0.276–0.632)	0.524 (0.369–0.679)	0.475 (0.375–0.575)	0.451 (0.349–0.554)

ALBI, albumin–bilirubin; CABLE, CRP, albumin, bilirubin, lymphocytes, ECOG, and EHS; CRAFITY, CRP and AFP in immunotherapy; EZ-ALBI, easy-ALBI; GPS, Glasgow Prognostic Score; mALBI, modified ALBI; $Model_i$, Cox PH model derived from the imputed training set; NLR, neutrophil-to-lymphocyte ratio; PLR, platelet-to-lymphocyte ratio; PNI, prognostic nutritional index.

are routinely measured at the start of therapy with a + b. It combines clinical information about systemic inflammation, liver insufficiency, tumor distribution, nutritional status, and the general condition of patients with HCC. Albumin is part of ALBI, EZ-ALBI, mALBI, PNI, and GPS and is a well-known highly prognostic parameter in patients with cirrhosis.[9,23–26,28,29] Bilirubin is included in ALBI, EZ-ALBI, and mALBI, reflecting the liver parenchymal damage.[9,23–25] CRP is incorporated in the CRAFITY score and GPS, and reflects the inflammatory state, where higher values are associated with worse outcome.[10,26] Lymphocytes are part of PNI, NLR, and PLR.[11,28,29,31–35] Here, lower values at treatment start are associated with shorter survival. EHS reflects the systemic distribution of HCC, and, if positive, is frequently associated with worse prognosis. ECOG is the only not completely objective parameter included in the CABLE score. However, the prognostic value of the general condition of patients in oncology is well established.[43] In addition, the CABLE score discriminates only ECOG 0 *vs.* ECOG 1/2, which is comparatively clear in most patients in our opinion.

Strengths of this work are the large multinational and homogenous cohort, yielding an appropriate proportion of outcome events for the development of a new prediction model. In addition, the TRIPOD-driven statistically elaborate development procedure and the external validation increase the robustness of our study. Furthermore, the CABLE score only includes broadly available and cost-effective predictors. However, this study has several limitations that have to be acknowledged. First, the retrospective nature of the study. Second, we integrated patients with missing data in the development cohort. However, the TRIPOD statement clearly advocates including patients with missing data by using multiple imputation rather than conducting a complete-case analysis.[36,37] Third, some patients have received atezolizumab monotherapy intermittently for example because of an increased risk of bleeding. Fourth, the cohort was derived only from Western countries, which is reflected in the distribution of etiologies. Thus, the score needs to be validated in other countries not included in the current dataset. Fifth, while we could compare the CABLE score to various established scores in our study, we were not able to include several other previously published prognostic scores because of missing parameters in our data set (such as CAR (CRAFITY score and AFP-Response) classification,[44] HCC-GRIm (hepatocellular carcinoma modified Gustave Roussy Immune) score,[45] and ABE (atezolizumab plus bevacizumab) index.[46] Sixth, the categorization of patients into the training and validation cohorts based on the time of data reporting of the centers may have introduced bias as a result of changes in baseline characteristics of the cohort. However, we decided not to change our statistical analysis plan once we knew the results owing to good scientific practice.

It is an important finding that the CABLE score and the other here evaluated scores do not perform well at response prediction. This underscores the need for better biomarkers to sufficiently address this question. While there has been some success in this regard when considering changes in AFP during the first weeks of treatment (either alone[47,48] or in combination with ALBI[49]), such an approach is not a true response prediction but rather a response assessment using the serum tumor marker AFP. It also does not enable response prediction before the initiation of treatment which would be highly desirable and relevant for future clinical trials focusing on subgroups of patients with HCC who have a high chance of not responding to a + b. Therefore, the identification and validation of such predictive biomarkers remains an unmet medical need in the field of systemic treatment of HCC and should be pursued with high priority.

In this large multicenter study, we developed and validated the CABLE score to predict the individual prognosis of patients with uHCC undergoing 1L immunotherapy with a + b. To make the CABLE score usable in daily practice we have launched a web-based calculator which computes survival probabilities of this patient group at 6, 12, and 18 months. Further external and geographically diverse validation is needed to confirm the performance of the CABLE score and its incremental value compared with other models, in particular the ALBI score. The validation studies should also focus on subgroup analysis, for example in patients with Child-Pugh A. In the future, the CABLE score may be used for prognostic assessment in clinical practice and as a stratification tool in clinical trials evaluating systemic treatments of HCC.

Affiliations

[1]Department of Internal Medicine I, University Medical Center of the Johannes Gutenberg University Mainz, Mainz, Germany; [2]Institute of Medical Biometry, Epidemiology and Informatics of the Johannes Gutenberg University Mainz, Mainz, Germany; [3]Department of Oncology, Mayo Clinic, Rochester, MN, USA; [4]Department of Hepatology and Gastroenterology, Charité–Universitätsmedizin Berlin, Campus Virchow-Klinikum and Campus Charité Mitte, Berlin, Germany; [5]Division of Gastroenterology & Hepatology, Department of Medicine III, Medical University of Vienna, Vienna, Austria; [6]Department of Gastroenterology and Hepatology, Essen University Hospital, Essen, Germany; [7]Department of Medicine II, Division of Hepatology, Leipzig University Medical Center, Leipzig, Germany; [8]Department of Gastroenterology, Hepatology and Infectious Diseases, Otto von Guericke University Hospital, Magdeburg, Germany; [9]I. Department of Internal Medicine, Gastroenterology & Hepatology, University Medical Center Hamburg-Eppendorf, Hamburg, Germany; [10]Medical Clinic 1, University Hospital, Goethe-University Frankfurt, Frankfurt am Main, Germany; [11]Medical Department III, University Hospital RWTH Aachen, Aachen, Germany; [12]Clinical Department for Internal Medicine II, Department of Clinical Medicine, TUM School of Medicine and Health, University Medical Center, Technical University of Munich, Germany; [13]Department of Internal Medicine I, University of Ulm, Ulm, Germany; [14]Clinic for Gastroenterology, Hepatology and Infectious Diseases, University Hospital Düsseldorf, Medical Faculty of Heinrich Heine University Düsseldorf, Düsseldorf, Germany; [15]Department of Surgery & Cancer, Faculty of Medicine, Imperial College London, Hammersmith Hospital, London, UK; [16]Department of Biomedical Sciences, Humanitas University, Pieve Emanuele (Milan), Italy; [17]Medical Oncology and Hematology Unit, Humanitas Cancer Center, IRCCS Humanitas Research Hospital, Rozzano (Milan), Italy; [18]Department of Internal Medicine and Gastroenterology (IMuG), Hepatology, Endocrinology, Rheumatology and Nephrology including Centralized Emergency Department (ZAE), Klinikum Klagenfurt am Worthersee, Klagenfurt, Kärnten, Austria; [19]Department of Medicine II, Medical Center University of Freiburg, Freiburg, Germany; [20]Department of Medicine II, University Hospital, Ludwig Maximilian University Munich, Munich, Germany; [21]Department of Gastroenterology and Hepatology, University of Cologne, Cologne, Germany; [22]Department of Diagnostic and Interventional Radiology, University Medical Center of the Johannes Gutenberg University Mainz, Mainz, Germany; [23]Institute of Interventional Radiology, University Hospital Schleswig-Holstein, Lübeck, Germany; [24]Abdominal Imaging Division, Department of Radiology, Mayo Clinic, Rochester, MN, USA; [25]Division of Hepatology, Division of Clinical Bioinformatics, Department of Medicine II, Medical Faculty Mannheim, Heidelberg University, Mannheim, Germany; [26]Clinical Cooperation Unit Healthy Metabolism Medical Faculty Mannheim, Heidelberg University, Mannheim, Germany; [27]Department of Medicine II, Medical Faculty Mannheim, Heidelberg University, Mannheim, Germany; [28]Department of Internal Medicine 1, University Hospital Schleswig-Holstein, Lübeck, Germany; [29]Division of Hepatology, Department of Medicine II, University Hospital Würzburg, Würzburg, Germany; [30]Division of Oncology, Department of Translational Medicine, University of Piemonte Orientale, Novara, Italy; [31]Department of Internal Medicine 1, Division for Gastroenterology, Hepatology, Infectiology, Gastrointestinal Oncology and Geriatrics, University Hospital of Tübingen, Tübingen, Germany; [32]Department I of Internal Medicine, University Hospital of Bonn, Bonn, Germany; [33]Division of Gastroenterology and Hepatology, Mayo Clinic Rochester, Rochester, MN, USA

Abbreviations

1L, first-line; a + b, atezolizumab and bevacizumab; AFP, alpha-fetoprotein; ALBI, albumin–bilirubin; BCLC, Barcelona Clinic Liver Cancer; CR, complete response; CRAFITY, CRP and AFP in immunotherapy; CRP, C-reactive protein; DCR, disease control rate; ECOG, Eastern Cooperative Oncology Group; EHS, extrahepatic spread; EZ-ALBI, easy-ALBI; GPS, Glasgow Prognostic Score; INR, international normalized ratio; mALBI, modified ALBI; MASH, metabolic dysfunction-associated steatohepatitis; MASLD, metabolic dysfunction-associated steatotic liver disease; MR, mixed response; NLR, neutrophil-to-lymphocyte ratio; ORR, overall response rate; OS, overall survival; PD, progressive disease; PD-L1, programmed death-ligand 1; PFS, progression-free survival; PLR, platelet-to-lymphocyte ratio; PNI, Prognostic Nutritional Index; PR, partial response; SBRT, stereotactic body radiation therapy; SD, stable disease; SIRT, selective internal radiation therapy; TACE, transarterial chemoembolization; TAE, transarterial embolization; TARE, transarterial radioembolization; TRIPOD, Transparent Reporting of a multivariable prediction model for Individual Prognosis or Diagnosis; uHCC, unresectable HCC; VEGF, vascular endothelial growth factor.

Financial support

This work was not supported by any grant or funding source.

Conflicts of interest

SJG has received travel expenses from Ipsen and Gilead. NA has received travel support and speaker fees from AbbVie and Gilead. FFo has received honoraria for lectures from AstraZeneca, Lilly, MSD, Pfizer, and Roche. He has served as advisory board member to AstraZeneca, BMS, Eisai, and Roche and has received travel support from Merck KGaA and Servier. JUM received honoraria from AstraZeneca, Eisai, MSD, MERZ, AbbVie, and Roche. DB has received lecture and speaker fees from W.L. Gore & Associates GmbH, the Falk Foundation Germany, and a travel grant from Gilead Science. KB has received honoraria for lectures from Ipsen. NHT has received honorarium (to the institution) from Helsinn and is a consultant for AZ, Genentech, and TEMPUS. FPR has received honoraria for lectures, consulting activities, and travel support from the Falk Foundation, AbbVie, Gilead, Ipsen, AstraZeneca, Roche, and Novartis. EDT has served as a paid consultant for AstraZeneca, Bayer, BMS, Eisai, Eli Lilly & Co, Pfizer, Ipsen, and Roche. He has received reimbursement of meeting attendance fees and travel expenses from Arqule, AstraZeneca, BMS, Bayer, Celsion, and Roche, and lecture honoraria from BMS and Falk. In addition, he has received third-party funding for scientific research from Arqule, AstraZeneca, BMS, Bayer, Eli Lilly, and Roche. NBK has received reimbursement of meeting attendance fees and travel expenses from Eisai and a lecture honorarium from Falk and AstraZeneca. KS has received honoraria for lectures from AstraZeneca, MSD. He has served as advisory board member to AstraZeneca, MSD, Servier, Eisai, and Roche. LM has received an honorarium for lecture from Bayer. UE has received honoraria for lectures from AstraZeneca, Eisai, the Falk Foundation, Ipsen, MSD, and Novartis. She has served as advisory board member to AstraZeneca, Bayer, MSD, and Eisai and has received travel support from AstraZeneca and Biotest. LR received consulting fees from AstraZeneca, Basilea, Bayer, BMS, Eisai, Elevar Therapeutics, Exelixis, Genenta, Hengrui, Incyte, Ipsen, IQVIA, Jazz Pharmaceuticals, MSD, Nerviano Medical Sciences, Roche, Servier, Taiho Oncology, Zymeworks; lecture fees from AstraZeneca, Bayer, BMS, Eisai, Incyte, Ipsen, Merck Serono, Roche, Servier; travel expenses from AstraZeneca; research grants (to Institution) from Agios, AstraZeneca, BeiGene, Eisai, Exelixis, Fibrogen, Incyte, Ipsen, Lilly, MSD, Nerviano Medical Sciences, Roche, Servier, Zymeworks. AD received educational support for congress attendance and consultancy fees from Roche, and speaker fees from Roche, AstraZeneca, Eisai, and Chugai. FFi has received travel support from Ipsen, AbbVie, AstraZeneca and speaker's fees from AbbVie, MSD, Ipsen, Astra. MP received speaker honoraria from Bayer, BMS, Eisai, Ipsen, Lilly, MSD, and Roche; he is a consultant for AstraZeneca, Bayer, BMS, Eisai, Ipsen, Lilly, MSD, and Roche; he received grants from Roche and BMS; he received travel support from Bayer, BMS, Ipsen, and Roche. MPR has received consulting fees from Bayer, BMS, Boehringer-Ingelheim, Eisai, Gilead, Intercept-Advanz, Ipsen, Lilly, MSD, Roche, Sanofi and was an investigator for Bayer, BMS, Boehringer-Ingelheim, Exelixis, Eisai, Falk, Gilead, Lilly, Ipsen, Novartis, and Roche. DJP received lecture fees from Bayer Healthcare, Eisai, BMS, Roche, Boston Scientific, travel expenses from BMS and Bayer Healthcare; consulting fees for Mina Therapeutics, DaVolterra, Mursla, Ipsen, Exact Sciences, Avamune, Eisai, Roche, Starpharma, LiFT biosciences and AstraZeneca; received research funding (to institution) from MSD, GSK, and BMS. LSJ received speaker honoraria from Falk Foundation, Boston Scientific, AbbVie, and AstraZeneca. She is consultant for Boston Scientific and AstraZeneca. She received travel support from Biotest and AbbVie. MAGC has contributed to advisory boards for Roche, Eisai, MSD, BMS, AZ, Amgen and Servier (these activities have no potential conflicts of interest with the manuscript). AW received compensations as a member of scientific advisory boards for Bayer, BMS, Eisai, and Sanofi and served as a speaker for Leo Pharma, Eisai, Ipsen, and Roche and received travel support from Merck and Servier. RK has received consultancy fees from Boston Scientific, Bristol-Myers Squibb, Guerbet, Roche, and SIRTEX, and lecture fees from AstraZeneca, BTG, Eisai, Guerbet, Ipsen, Roche, Siemens, SIRTEX, and MSD Sharp & Dohme (none are related to this work). PRG received honoraria for lectures and advisory boards from Bayer, BMS, MSD, AstraZeneca, Lilly, Ipsen, Roche, Eisai, Guerbet, and Boston Scientific. SII served as advisory board member for AstraZeneca and received consulting fees from AstraZeneca. All other authors disclose no potential financial or non-financial conflict of interests.

Please refer to the accompanying ICMJE disclosure forms for further details.

Authors' contributions

Performed research: SJG, JG, FA, BS, CL, SL, FS, VJ, TF, VH, NA, CS, KB, JS, SL, JK, CAMF, AD, VZ, FH, NR, NBK, ER, LM, AW, RK, PRG, NGH, SSKV, AT, ET, DTW, JUM, DB, MPR, AG, FPR, LR, DJP, CR, TE, MB, VS, UE, MLB, FFi, MAGC, JF, KS, MV, FB, LSJ, MP, RM, SII, FFo. Contributed to acquisition of data: SJG, JG, FA, BS, CL, SL, FS, VJ, TF, VH, NA, CS, KB, JS, SL, JK, CAMF, AD, VZ, FH, NR, NBK, ER, LM, AW, RK, PRG, NGH, SSKV, AT, ET, DTW, JUM, DB, MPR, AG, FPR, LR, DJP, CR, TE, MB, VS, UE, MLB, FFi, MAGC, JF, KS, MV, FB, LSJ, MP, RM, SII, FFo. Designed the experiments and analyzed the data: PM, IS, SJG, FFo. Contributed reagents/materials/analysis tools: PM, IS, SJG, FFo. Statistical analysis: PM, IS, SJG. Wrote the paper: SJG, FFo, PM.

Data availability statement

The data that support the findings of this study are available from the corresponding author (FF) upon reasonable request.

Acknowledgements

SJG and LM were supported by the Clinician Scientist Fellowship “Else Kröner Research College: 2018_Kolleg.05”. NHT is a recipient of the K23MD017217-01A1 award. AT is supported by the State Ministry of Baden-Wuerttemberg for Sciences, Research and Arts supporting the Clinical Cooperation Unit Healthy Metabolism, Center for Preventive Medicine and Digital Health (grant identifier: CCU Healthy Metabolism) and the Federal Ministry of Education and Research for development of an integrated quality controlled data set for HCC (BMBF, identifier 01KD2214). SKV acknowledges support from NIH (R01 EB001981), U.S. Department of Defense (W81XWH-19-1-0583-01); and receives textbook royalties from Springer. KB is supported by grants from the German Research Foundation (BO 5548/1-1 and TRR179/3, 25) and German Cancer Aid (70115059). AD is supported by the National Institute for Health Research (NIHR) Imperial BRC, by grant funding from the European Association for the Study of the Liver (2021 Andrew Burroughs Fellowship) and from Cancer Research UK (RCCPDB- Nov21/100008). DJP is supported by grant funding from the Wellcome Trust Strategic Fund (PS3416), the Associazione Italiana per la Ricerca sul Cancro (AIRC MFAG 25697), the Roger Williams Foundation for Liver Disease (Small Project Grant) and acknowledges grant support from the Cancer Treatment and Research Trust (CTRT), infrastructural support by the Imperial Experimental Cancer Medicine Centre and the NIHR Imperial Biomedical Research Centre. The views expressed are those of the authors and not necessarily those of the NIHR or the Department of Health and Social Care. NR is supported by the Berta-Ottenstein Programme, Faculty of Medicine, University of Freiburg.

Supplementary data

Supplementary data to this article can be found online at https://doi.org/10.1016/j.jhepr.2024.101295.

References

Author names in bold designate shared co-first authorship

[1] Sung H, Ferlay J, Siegel RL, et al. Global cancer statistics 2020: GLOBOCAN estimates of incidence and mortality worldwide for 36 cancers in 185 countries. CA Cancer J Clin 2021;71:209–249.
[2] Rumgay H, Arnold M, Ferlay J, et al. Global burden of primary liver cancer in 2020 and predictions to 2040. J Hepatol 2022;77:1598–1606.
[3] Finn RS, Qin S, Ikeda M, et al. IMbrave150: updated overall survival (OS) data from a global, randomized, open-label phase III study of atezolizumab (atezo) + bevacizumab (bev) versus sorafenib (sor) in patients (pts) with unresectable hepatocellular carcinoma (HCC). J Clin Oncol 2021;39(3 Suppl):267.
[4] Finn RS, Qin S, Ikeda M, et al. Atezolizumab plus bevacizumab in unresectable hepatocellular carcinoma. N Engl J Med 2020;382:1894–1905.
[5] Cheng A-L, Qin S, Ikeda M, et al. Updated efficacy and safety data from IMbrave150: atezolizumab plus bevacizumab vs. sorafenib for unresectable hepatocellular carcinoma. J Hepatol 2022;76:862–873.
[6] Bruix J, Chan SL, Galle PR, et al. Systemic treatment of hepatocellular carcinoma: an EASL position paper. J Hepatol 2021;75:960–974.
[7] Singal AG, Llovet JM, Yarchoan M, et al. AASLD Practice Guidance on prevention, diagnosis, and treatment of hepatocellular carcinoma. Hepatology 2023;78:1922–1965.
[8] Sanghera C, Teh JJ, Pinato DJ. The systemic inflammatory response as a source of biomarkers and therapeutic targets in hepatocellular carcinoma. Liver Int 2019;39:2008–2023.
[9] Johnson PJ, Berhane S, Kagebayashi C, et al. Assessment of liver function in patients with hepatocellular carcinoma: a new evidence-based approach - the ALBI grade. J Clin Oncol 2015;33:550–558.
[10] Scheiner B, Pomej K, Kirstein MM, et al. Prognosis of patients with hepatocellular carcinoma treated with immunotherapy - development and validation of the CRAFITY score. J Hepatol 2022;76:353–363.
[11] Wu YL, Fulgenzi CAM, D'Alessio A, et al. Neutrophil-to-lymphocyte and platelet-to-lymphocyte ratios as prognostic biomarkers in unresectable hepatocellular carcinoma treated with atezolizumab plus bevacizumab. Cancers 2022;14:5834.
[12] Hiraoka A, Kumada T, Tada T, et al. Nutritional index as prognostic indicator in patients receiving lenvatinib treatment for unresectable hepatocellular carcinoma. Oncology 2020;98:295–302.
[13] Johnson PJ, Pinato DJ, Kalyuzhnyy A, et al. Breaking the Child-Pugh dogma in hepatocellular carcinoma. J Clin Oncol 2022;40:2078–2082.
[14] Peng Y, Wei Q, He Y, et al. ALBI versus Child-Pugh in predicting outcome of patients with HCC: a systematic review. Expert Rev Gastroenterol Hepatol 2020;14:383–400.
[15] **Pinato DJ**, **Sharma R**, Allara E, et al. The ALBI grade provides objective hepatic reserve estimation across each BCLC stage of hepatocellular carcinoma. J Hepatol 2017;66:338–346.
[16] Chan AWH, Kumada T, Toyoda H, et al. Integration of albumin-bilirubin (ALBI) score into Barcelona Clinic Liver Cancer (BCLC) system for hepatocellular carcinoma. J Gastroenterol Hepatol 2016;31:1300–1306.
[17] Kudo M, Finn RS, Cheng A-L, et al. Albumin-bilirubin grade analyses of atezolizumab plus bevacizumab versus sorafenib in patients with unresectable hepatocellular carcinoma: a post hoc analysis of the phase III IMbrave150 study. Liver Cancer 2023;12:479–493.
[18] Vogel A, Chan S, Furuse J, et al. O-5 Outcomes by baseline liver function in patients with unresectable hepatocellular carcinoma treated with tremelimumab and durvalumab in the phase 3 HIMALAYA study. Ann Oncol 2022;33:S380–S381.
[19] Vogel A, Frenette C, Sung M, et al. Baseline liver function and subsequent outcomes in the phase 3 REFLECT study of patients with unresectable hepatocellular carcinoma. Liver Cancer 2021;10:510–521.
[20] Chan SL, Miksad R, Cicin I, et al. Outcomes based on albumin-bilirubin (ALBI) grade in the phase III CELESTIAL trial of cabozantinib versus placebo in patients with advanced hepatocellular carcinoma (HCC). Ann Oncol 2019;30:ix45–i46.
[21] Kudo M, Galle PR, Brandi G, et al. Effect of ramucirumab on ALBI grade in patients with advanced HCC: results from REACH and REACH-2. JHEP Rep 2021;3:100215.
[22] **Demirtas CO**, **D'Alessio A**, Rimassa L, et al. ALBI grade: evidence for an improved model for liver functional estimation in patients with hepatocellular carcinoma. JHEP Rep 2021;3:100347.
[23] Kariyama K, Nouso K, Hiraoka A, et al. EZ-ALBI score for predicting hepatocellular carcinoma prognosis. Liver Cancer 2020;9:734–743.
[24] Hiraoka A, Kumada T, Tsuji K, et al. Validation of modified ALBI grade for more detailed assessment of hepatic function in hepatocellular carcinoma patients: a multicenter analysis. Liver Cancer 2019;8:121–129.
[25] Hsu W-F, Hsu S-C, Chen T-H, et al. Modified albumin-bilirubin model for stratifying survival in patients with hepatocellular carcinoma receiving anticancer therapy. Cancers 2022;14:5083.
[26] Tada T, Kumada T, Hiraoka A, et al. New prognostic system based on inflammation and liver function predicts prognosis in patients with advanced unresectable hepatocellular carcinoma treated with atezolizumab plus bevacizumab: a validation study. Cancer Med 2023;12:6980–6993.
[27] Pinato DJ, Stebbing J, Ishizuka M, et al. A novel and validated prognostic index in hepatocellular carcinoma: the inflammation based index (IBI). J Hepatol 2012;57:1013–1020.
[28] Rimini M, Persano M, Tada T, et al. Role of the prognostic nutritional index in predicting survival in advanced hepatocellular carcinoma treated with atezolizumab plus bevacizumab. Oncology 2023;101:283–291.
[29] Tada T, Kumada T, Hiraoka A, et al. Nutritional status is associated with prognosis in patients with advanced unresectable hepatocellular carcinoma treated with atezolizumab plus bevacizumab. Oncology 2023;101:270–282.
[30] Pinato DJ, North BV, Sharma R. A novel, externally validated inflammation-based prognostic algorithm in hepatocellular carcinoma: the prognostic nutritional index (PNI). Br J Cancer 2012;106:1439–1445.

[31] Tada T, Kumada T, Hiraoka A, et al. Neutrophil-lymphocyte ratio predicts early outcomes in patients with unresectable hepatocellular carcinoma treated with atezolizumab plus bevacizumab: a multicenter analysis. Eur J Gastroenterol Hepatol 2022;34:698–706.
[32] Wang J-H, Chen Y-Y, Kee K-M, et al. The prognostic value of neutrophil-to-lymphocyte ratio and platelet-to-lymphocyte ratio in patients with hepatocellular carcinoma receiving atezolizumab plus bevacizumab. Cancers 2022;14:343.
[33] Ochi H, Kurosaki M, Joko K, et al. Usefulness of neutrophil-to-lymphocyte ratio in predicting progression and survival outcomes after atezolizumab-bevacizumab treatment for hepatocellular carcinoma. Hepatol Res 2023;53:61–71.
[34] Eso Y, Takeda H, Taura K, et al. Pretreatment neutrophil-to-lymphocyte ratio as a predictive marker of response to atezolizumab plus bevacizumab for hepatocellular carcinoma. Curr Oncol 2021;28:4157–4166.
[35] Jost-Brinkmann F, Demir M, Wree A, et al. Atezolizumab plus bevacizumab in unresectable hepatocellular carcinoma: results from a German real-world cohort. Aliment Pharmacol Ther 2023;57:1313–1325.
[36] Collins GS, Reitsma JB, Altman DG, et al. Transparent reporting of a multivariable prediction model for individual prognosis or diagnosis (TRIPOD): the TRIPOD statement. Ann Intern Med 2015;162:55–63.
[37] Moons KGM, Altman DG, Reitsma JB, et al. Transparent Reporting of a multivariable prediction model for Individual Prognosis or Diagnosis (TRIPOD): explanation and elaboration. Ann Intern Med 2015;162 :W1–W73.
[38] Åberg F, Luukkonen PK, But A, et al. Development and validation of a model to predict incident chronic liver disease in the general population: the CLivD score. J Hepatol 2022;77:302–311.
[39] Marra G, Wood SN. Practical variable selection for generalized additive models. Comput Stat Data Anal 2011;55:2372–2387.
[40] Blanche P, Dartigues J-F, Jacqmin-Gadda H. Estimating and comparing time-dependent areas under receiver operating characteristic curves for censored event times with competing risks. Stat Med 2013;32:5381–5397.
[41] Uno H, Cai T, Pencina MJ, et al. On the C-statistics for evaluating overall adequacy of risk prediction procedures with censored survival data. Stat Med 2011;30:1105–1117.
[42] Bettinger D, Sturm L, Pfaff L, et al. Refining prediction of survival after TIPS with the novel Freiburg index of post-TIPS survival. J Hepatol 2021;74:1362–1372.
[43] Oken MM, Creech RH, Tormey DC, et al. Toxicity and response criteria of the eastern cooperative oncology group. Am J Clin Oncol 1982;5:649–655.
[44] **Teng W**, **Lin C-C**, Su C-W, et al. Combination of CRAFITY score with Alpha-fetoprotein response predicts a favorable outcome of atezolizumab plus bevacizumab for unresectable hepatocellular carcinoma. Am J Cancer Res 2022;12:1899–1911.
[45] Hatanaka T, Naganuma A, Hiraoka A, et al. The hepatocellular carcinoma modified Gustave Roussy Immune score (HCC-GRIm score) as a novel prognostic score for patients treated with atezolizumab and bevacizumab: a multicenter retrospective analysis. Cancer Med 2023;12:4259–4269.
[46] **Persano M**, **Rimini M**, Tada T, et al. Identification of atezolizumab plus bevacizumab prognostic index via recursive partitioning analysis in HCC: the ABE index. Anticancer Res 2023;43:1599–1610.
[47] Kuzuya T, Kawabe N, Hashimoto S, et al. Early changes in alpha-fetoprotein are a useful predictor of efficacy of atezolizumab plus bevacizumab treatment in patients with advanced hepatocellular carcinoma. Oncology 2022;100:12–21.
[48] **Lu L**, **Zheng P**, **Pan Y**, et al. Trajectories of α-fetoprotein and unresectable hepatocellular carcinoma outcomes receiving atezolizumab plus bevacizumab: a secondary analysis of IMbrave150 study. Br J Cancer 2023;129:620–625.
[49] Campani C, Bamba-Funck J, Campion B, et al. Baseline ALBI score and early variation of serum AFP predicts outcomes in patients with HCC treated by atezolizumab-bevacizumab. Liver Int 2023;43:708–717.

Keywords:Hepatocellular carcinoma; Cirrhosis; Systemic therapy; Prediction model; Outcome; PD-L1; VEGF; Development; Validation; CABLE score; Immunotherapy; Immune checkpoint inhibitors.

Received 15 April 2024; received in revised form 28 November 2024; accepted 29 November 2024; Available online 5 December 2024

Journal of Hepatology, Volume 7

Supplemental information

Evaluation of prognostic scores in patients with HCC undergoing first-line immunotherapy with atezolizumab and bevacizumab

Simon Johannes Gairing, Philipp Mildenberger, Jennifer Gile, Fabian Artusa, Bernhard Scheiner, Catherine Leyh, Sabine Lieb, Friedrich Sinner, Vincent Jörg, Thorben Fruendt, Vera Himmelsbach, Nada Abedin, Cennet Sahin, Katrin Böttcher, Jasmin Schuhbaur, Simon Labuhn, James Korolewicz, Claudia A.M. Fulgenzi, Antonio D'Alessio, Valentina Zanuso, Florian Hucke, Natascha Röhlen, Najib Ben Khaled, Eleonora Ramadori, Lukas Müller, Arndt Weinmann, Roman Kloeckner, Peter Robert Galle, Nguyen H. Tran, Sudhakar K. Venkatesh, Andreas Teufel, Matthias Ebert, Enrico N. De Toni, Dirk-Thomas Waldschmidt, Jens U. Marquardt, Dominik Bettinger, Markus Peck-Radosavljevic, Andreas Geier, Florian P. Reiter, Lorenza Rimassa, David J. Pinato, Christoph Roderburg, Thomas Ettrich, Michael Bitzer, Veit Scheble, Ursula Ehmer, Marie-Luise Berres, Fabian Finkelmeier, Maria Angeles Gonzalez-Carmona, Johann von Felden, Kornelius Schulze, Marino Venerito, Florian van Bömmel, Leonie S. Jochheim, Matthias Pinter, Raphael Mohr, Sumera I. Ilyas, Irene Schmidtmann, and Friedrich Foerster

Evaluation of prognostic scores in patients with HCC undergoing first-line immunotherapy with atezolizumab and bevacizumab

Simon Johannes Gairing, Philipp Mildenberger, Jennifer Gile, Fabian Artusa, Bernhard Scheiner, Catherine Leyh, Sabine Lieb, Friedrich Sinner, Vincent Jörg, Thorben Fruendt, Vera Himmelsbach, Nada Abedin, Cennet Sahin, Katrin Böttcher, Jasmin Schuhbaur, Simon Labuhn, James Korolewicz, Claudia A.M. Fulgenzi, Antonio D'Alessio, Valentina Zanuso, Florian Hucke, Natascha Röhlen, Najib Ben Khaled, Eleonora Ramadori, Lukas Müller, Arndt Weinmann, Roman Kloeckner, Peter Robert Galle, Nguyen H. Tran, Sudhakar K. Venkatesh, Andreas Teufel, Matthias Ebert, Enrico N. De Toni, Dirk-Thomas Waldschmidt, Jens U. Marquardt, Dominik Bettinger, Markus Peck-Radosavljevic, Andreas Geier, Florian P. Reiter, Lorenza Rimassa, David J. Pinato, Christoph Roderburg, Thomas Ettrich, Michael Bitzer, Veit Scheble, Ursula Ehmer, Marie-Luise Berres, Fabian Finkelmeier, Maria Angeles Gonzalez-Carmona, Johann von Felden, Kornelius Schulze, Marino Venerito, Florian van Bömmel, Leonie S. Jochheim, Matthias Pinter, Raphael Mohr, Sumera I. Ilyas, Irene Schmidtmann, Friedrich Foerster

Table of contents

Supplementary methods 2

Supplementary tables 7

Supplementary figures 22

TRIPOD checklist 26

Supplementary methods

For statistical analysis, R (version 4.1.3, a language and environment for statistical computing. R Foundation for Statistical Computing, Vienna, Austria. https://www.R-project.org/.), and RStudio (version 2023.06.2+561, Integrated Development for R. RStudio, PBC, Boston, MA, http://www.rstudio.com/.) were used.

Study design and patients

The training set included patients from Berlin (DE), Essen (DE), Mainz (DE), Frankfurt (DE), Ulm (DE), Freiburg (DE), Munich (TUM, DE), Bonn (DE), Magdeburg (DE), Aachen (DE), Vienna (AT), and Rochester (US). The external validation set was derived from Lübeck (DE), Würzburg (DE), Cologne (DE), Düsseldorf (DE), Mannheim (DE), Tübingen (DE), Munich (LMU, DE), Leipzig (DE), Hamburg (DE), Klagenfurt (AT), Milan (IT), London (UK).

All patients ≥ 18 years old with a radiologically and/or histologically confirmed uHCC undergoing immunotherapy with a+b were screened for eligibility. Exclusion criteria were: a+b not 1L systemic therapy, Child-Pugh C, ECOG (Eastern Cooperative Oncology Group) status > 2, no follow-up data available, simultaneous brachytherapy, no baseline laboratory values within 30 days prior to the treatment start available, mixed hepatocellular/cholangiocellular carcinoma histology, simultaneous chemotherapy due to second malignancy, and a history of liver transplantation (details on how many patients are excluded respectively are given in **Fig. 1**). Some patient data from some centers have been used as part of other research projects [1–7].

Outcome

The outcome of interest was death from any cause. Information on patients' vital status was obtained from hospital records. Baseline was defined at the start of immunotherapy with a+b. Patients still alive at the time of data cut-off were censored on the day of last contact. Overall survival was calculated from the day of the first cycle of a+b until death or last contact. Patients who underwent liver transplantation following immunotherapy with a+b were censored on the day of transplantation. As only two patients underwent liver transplantation, no competing risk model was used.

Candidate predictors

Candidate predictors included patient characteristics, tumor characteristics and several laboratory values all of which were determined at baseline. **Table S1** provides an overview of variables which have been extracted for all patients. The candidate predictors were selected based on available evidence in the literature and because they are easily and cost-effective available for every HCC patient before treatment start. If laboratory values were not determined at the day of the first a+b cycle, values up to 30 days before treatment start were accepted. If multiple measurements for a laboratory value were available within 30 days, the one closest to the day of the first cycle was selected. The assessment of predictors was not blinded. The presence of cirrhosis was determined by histology or typical findings on imaging, endoscopy and/or medical history.

Response assessment

Tumor response following treatment with a+b was assessed by computed tomography or magnetic resonance imaging. Response was graded as complete response (CR), partial response (PR), stable disease (SD), progressive disease (PD) or mixed response (MR) according to the modified Response Evaluation Criteria in Solid Tumors

(mRECIST) or, if not available, according to the local standard. MR was defined as progression at one site, while shrinking at another. The objective response rate (ORR) included patients with CR and PR, the disease control rate (DCR) included patients with CR, PR, SD, and MR.

Sample size

Given the retrospective study design and the routinely collected data, all patients meeting the eligibility criteria without fulfilling exclusion criteria were included. Consequently, a formal sample size calculation was not conducted.

Calculation of scores

The ALBI score was calculated as follows: ALBI = ($\log_{10}$ bilirubin [µmol/L] × 0.66) + (albumin [g/L] × -0.085) [8]. Regarding the CRAFITY score, CRP levels ≥1 mg/dl and AFP levels AFP ≥100 ng/ml were each scored with one point, yielding three risk groups [1]. The PNI was calculated using the following equation: PNI = (albumin [g/L]) + (0.005 × total lymphocyte count per mm^3) [9, 10]. The NLR was calculated by dividing the total neutrophil count per nL by the total lymphocyte count per nL, the PLR by dividing the platelet count per nL by the total lymphocyte count per nL [11]. The EZ-ALBI score was calculated by (bilirubin [mg/dl] – (9 × albumin [g/dL])) [12]. mALBI grades were defined as described in Hiraoka et al. [13]. The Glasgow Prognostic Score (GPS) was calculated as detailed in [14]. Seven centers did not provide Child-Pugh stages for patients without cirrhosis. Here, the Child-Pugh stage was calculated using albumin, INR, and bilirubin.

Statistical analysis

For descriptive statistics, continuous data are provided as median with range (min – max). Categorical data are given as frequencies with percentages. For the comparison of the training and validation set, a Chi square test was used for categorial data and a Mann-Whitney U test or Students t-test for continuous data depending on data distribution. The reverse Kaplan-Meier method was used to estimate median follow-up time.

Modification of candidate predictors

AFP, creatinine, and neutrophils had few extreme values and were therefore trimmed as follows: AFP: 13 cases with an AFP level > 100,000 ng/ml were set to 100,000 ng/ml; creatinine: 10 cases with a creatinine level > 3 mg/dl were set to 3 mg/dl; neutrophils: 2 cases with absolute neutrophil counts > 20/nl were set to 20/nl. AFP, INR, bilirubin, creatinine, CRP, and platelets were log_{10}-transformed due to their skewed distributions.

Missing data

Candidate predictors with less than 10% of missing values were imputed in 5 multiple imputations. This was done with predictive mean matching in the R-package mice. The proportion of missing data in each candidate predictor are given in **Table 1**.

The variables with more than 0% but ≤ 10% missingness were INR, albumin, bilirubin, creatinine, AFP, neutrophils and platelets (**Table 1**). These variables, along with age, sex, center, cirrhosis, pretreatment, ECOG, extrahepatic spread (EHS), macrovascular invasion (MVI), time-to-event and status were used to derive missing values in this context. CRP, lymphocytes and monocytes had more than 10% missing values in the training set. Thus, these variables were neither imputed nor included in the initial model building. After building a preliminary model based on the imputed data set, we analyzed

whether these variables could enhance the predictive performance on the complete-case training set. This approach was similar to that of Åberg et al. when building the CLivD score [15]. Regarding the outcome (death from any cause), no data were missing.

Supplementary tables

Table S1: Candidate predictors at baseline.

Patient characteristics	Tumor characteristics	Laboratory values
Gender (male (1), female (2))	Presence of extrahepatic spread (0/1)	Alpha-fetoprotein (AFP), ng/ml
Age (years)	Presence of macrovascular invasion (0/1)	International Normalized Ratio (INR)
Etiology		Albumin (g/dl)
Presence of cirrhosis (0/1)		Bilirubin (mg/dl)
Pretreatments		Creatinine (mg/dl)
ECOG performance status (0, 1, 2, 3, 4)		C-reactive protein (CRP), mg/l
Child-Pugh stage (A, B, C)		Neutrophils (X/nl and in %)
BCLC stage (0, A, B, C, D)		Lymphocytes (X/nl and in %)
		Monocytes (X/nl and in %)
		Platelets (X/nl)

Table S2. Reasons for discontinuation of a+b.

	**Total cohort (n = 683)**
Ongoing therapy	173 (25.3%)
Progression	274 (40.1%)
Death	87 (12.7%)
Toxicity	58 (8.5%)
Patient request	22 (3.2%)
Unknown	22 (3.2%)
Decompensation/liver function decline	10 (1.5%)
Deterioration of general health	7 (1.0%)
Lost to follow-up	7 (1.0%)
CR	6 (0.9%)
Secondary surgical resection	5 (0.7%)
Toxicity/progression	3 (0.4%)
Bleeding and/or thrombotic event	3 (0.4%)
Liver transplantation	2 (0.3%)
Toxicity/death	1 (0.1%)
Toxicity/patient request	1 (0.1%)
Planned liver transplantation	1 (0.1%)
Insurance issues	1 (0.1%)

Abbreviations: CR, complete response.

Table S3. Radiological response in the training and validation cohort.

	Total cohort (N=683)	Training set (N=526)	Validation set (N=157)
**Tumor response**			
CR	24 (4.9%)	18 (5.2%)	6 (4.2%)
PR	129 (26.6%)	91 (26.5%)	38 (26.8%)
SD	187 (38.6%)	132 (38.5%)	55 (38.7%)
PD	140 (28.9%)	97 (28.3%)	43 (30.3%)
MR	5 (1.0%)	5 (1.5%)	0 (0%)
Missing	198 (29.0%)	183 (34.8%)	15 (9.6%)
**ORR**			
SD+PD	332 (68.5%)	234 (68.2%)	98 (69.0%)
CR+PR	153 (31.5%)	109 (31.8%)	44 (31.0%)
Missing	198 (29.0%)	183 (34.8%)	15 (9.6%)
**DCR**			
PD	140 (28.9%)	97 (28.3%)	43 (30.3%)
CR+PR+SD	345 (71.1%)	246 (71.7%)	99 (69.7%)
Missing	198 (29.0%)	183 (34.8%)	15 (9.6%)

Abbreviations: CR, complete response; PR, partial response; SD, stable disease; PD, progressive disease; MR, mixed response; ORR, objective response rate; DCR, disease control rate.

Table S4. Univariable Cox proportional hazards models.

Variable	Explained Deviance	Chi² test (p-value)
Age (years)	2.77%	0.4
Gender (male vs female)	2.20%	0.2
Etiology		
9 categories[a]	3.03%	0.2
Nonviral vs viral vs mixed etiology	1.93%	0.8
MASLD/MASH vs others	1.93%	0.3
Cirrhosis (yes vs no)	2.48%	0.041
Pretreatment (yes vs no)	2.65%	<0.001
ECOG	5.65%	<0.001
BCLC	3.92%	<0.001
Child-Pugh stage	6.42%	<0.001
EHS (yes vs no)	2.48%	0.002
MVI (yes vs no)	3.39%	<0.001
AFP, log10 (ng/ml)	3.45%	<0.001
Albumin (g/dl)	9.30%	<0.001
Bilirubin, log10 (mg/dl)	6.61%	<0.001
Creatinine, log10 (mg/dl)	2.56%	0.002
CRP, log10 (mg/l)	7.58%	<0.001
INR, log10	3.39%	<0.001
Platelets, log10 (/nl)	2.66%	0.007
Neutrophils (/nl)	3.48%	<0.001
Lymphocytes (/nl)	5.70%	<0.001
Monocytes (/nl)	2.19%	<0.001

[a]unknown/no underlying liver disease, HBV, HCV, alcohol, MASLD/MASH/metabolic, mixed etiology (viral + non-viral), mixed etiology (only non-viral), mixed (only viral), other etiologies. A Chi² test was carried out to assess statistical significance.

Table S5. Adjusted Cox PH model of the Model$_i$.

Variables	Beta[a]	95% CI	p-value
INR_{log10}	1.5	-0.28, 3.3	0.10
$Bilirubin_{log10}$	1.3	0.78, 1.8	<0.001
Pretreatment			
no	—	—	
yes	-0.29	-0.56, -0.02	0.035
ECOG			
0	—	—	
1	0.50	0.21, 0.79	<0.001
2	0.94	0.43, 1.5	<0.001
EHS			
no	—	—	
yes	0.47	0.20, 0.73	<0.001
s(Albumin)			<0.001
s($Creatinine_{log10}$)			0.032

Adjusted Cox PH model. [a] log(HR). Abbreviations: CI: confidence interval; INR: International Normalized Ratio; ECOG: Eastern Cooperative Oncology Group; EHS: extrahepatic spread; s: smoothing spline.

Table S6. Baseline characteristics of the complete-case training set.

	Overall (N=320)
**Age (years)**	
Median [Min, Max]	67.0 [25.0, 89.5]
**Gender**	
male	260 (81.3%)
female	60 (18.8%)
**ECOG**	
0	142 (44.4%)
1	152 (47.5%)
2	26 (8.1%)
**Cirrhosis**	
no	67 (20.9%)
yes	253 (79.1%)
**Etiology**	
unknown/no liver disease	36 (11.3%)
HBV	41 (12.8%)
HCV	53 (16.6%)
Harmful alcohol use	83 (25.9%)
MASLD/MASH	64 (20.0%)
Mixed (viral and non-viral)	16 (5.0%)
Mixed (only non-viral)	15 (4.7%)
Mixed (only viral)	1 (0.3%)
Other	11 (3.4%)
**BCLC**	
A	6 (1.9%)
B	70 (21.9%)
C	244 (76.3%)
**Pretreatment**	
no	148 (46.3%)
yes	172 (53.8%)
**History of surgery**	
no	255 (79.7%)
yes	65 (20.3%)
**History of ablation**	
no	284 (88.8%)
yes	36 (11.3%)
**History of TACE/TAE**	
no	219 (68.4%)
yes	101 (31.6%)
**History of SIRT/TARE**	
no	291 (90.9%)
yes	29 (9.1%)
**History of radiatio/SBRT**	
no	313 (97.8%)
yes	7 (2.2%)
**History of brachytherapy**	
no	310 (96.9%)
yes	10 (3.1%)
**Macrovascular invasion (MVI)**	
no	161 (50.3%)
yes	159 (49.7%)
**Extrahepatic spread (EHS)**	
no	199 (62.2%)
yes	121 (37.8%)
**Child-Pugh**	
A	229 (71.6%)
B	91 (28.4%)

	Overall (N=320)
**ALBI score**	
Median [Min, Max]	-2.31 [-3.61, -0.733]
**ALBI grade**	
1	103 (32.2%)
2	200 (62.5%)
3	17 (5.3%)
**mALBI grade**	
1	103 (32.2%)
2a	64 (20.0%)
2b	136 (42.5%)
3	17 (5.3%)
**EZ-ALBI**	
Median [Min, Max]	-31.4 [-45.2, -14.6]
**CRAFITY score**	
low	92 (28.8%)
intermediate	138 (43.1%)
high	90 (28.1%)
**GPS**	
GPS 0	113 (35.3%)
GPS 1	125 (39.1%)
GPS 2	82 (25.6%)
**PNI**	
Median [Min, Max]	42.3 [23.0, 63.1]
**NLR**	
Median [Min, Max]	3.69 [0.337, 117]
**PLR**	
Median [Min, Max]	152 [33.3, 3300]
**Bilirubin (mg/dl)**	
Median [Min, Max]	0.827 [0.160, 12.2]
**Albumin (g/dl)**	
Median [Min, Max]	3.65 [1.70, 5.16]
**INR**	
Median [Min, Max]	1.16 [0.900, 3.90]
**Creatinine (mg/dl)**	
Median [Min, Max]	0.870 [0.386, 8.00]
**CRP (mg/l)**	
Median [Min, Max]	11.2 [0.400, 279]
**AFP (ng/ml)**	
Median [Min, Max]	79.0 [1.00, 2070000]
**Platelets per nl**	
Median [Min, Max]	160 [34.0, 760]
**Neutrophils per nl**	
Median [Min, Max]	4.15 [0.550, 15.8]
**Lymphocytes per nl**	
Median [Min, Max]	1.08 [0.0700, 3.59]
**Monocytes per nl**	
Median [Min, Max]	0.584 [0.0490, 2.08]
Missing	1 (0.3%)

Abbreviations: ECOG, Eastern Cooperative Oncology Group; HBV, hepatitis B virus; HCV, hepatitis C virus; MASLD, Metabolic dysfunction–associated steatotic liver disease; MASH, Metabolic dysfunction–associated steatohepatitis; BCLC, Barcelona Clinic Liver Cancer; HCC; hepatocellular carcinoma, TACE, transarterial chemoembolization; TAE, transarterial embolization; SIRT, selective internal radiation therapy; TARE, transarterial radioembolization; SBRT, stereotactic body radiation therapy; ALBI, albumin-bilirubin; mALBI, modified ALBI; EZ-ALBI, easy-ALBI; CRAFITY, CRP and AFP in immunotherapy; GPS, Glasgow Prognostic Score; PNI, prognostic nutritional index; NLR, neutrophil-to-

lymphocyte ratio, PLR, platelet-to-lymphocyte ratio; INR, International Normalized Ratio; CRP, C-reactive protein; AFP, alpha-fetoprotein.

Table S7. Comparison of variables included in different prognostic models.

	Albumin	Bilirubin	Lymphocytes	Neutrophils	Platelets	CRP	AFP	Creatinine	INR	ECOG	EHS	Pretreatment
**CABLE score**	X	X	X			X				X	X	
**Model$_i$**	X	X						X	X	X	X	X
**ALBI [8]**	X	X										
**EZ-ALBI [12]**	X	X										
**mALBI [13]**	X	X										
**PNI [9, 10]**	X		X									
**CRAFITY [1]**						X	X					
**NLR [11]**			X	X								
**PLR [11]**			X		X							
**GPS [14]**	X					X						

Abbreviations: ALBI, albumin-bilirubin; EZ-ALBI, easy ALBI; mALBI, modified ALBI; PNI, prognostic nutritional index; CRAFITY, CRP and AFP in Immunotherapy; NLR, neutrophiles to lymphocytes ratio; PLR, platelets to lymphocytes ratio; GPS, Glasgow Prognostic Score; CRP, C-reactive protein; AFP, alpha-fetoprotein; INR, International Normalized Ratio; ECOG, Eastern Cooperative Oncology Group; EHS, extrahepatic spread.

Table S8. Time-dependent AUC of the CABLE score and Model$_i$ compared to several other prognostic models after 6, 12 and 18 months in the training set (internal validation).

Models	6 months	12 months	18 months
**CABLE score**	0.774	0.792	0.815
**Model$_i$**	0.761	0.75	0.793
**ALBI**	0.719	0.715	0.74
**EZ-ALBI**	0.715	0.715	0.732
**ALBI grades**	0.654	0.635	0.653
**mALBI**	0.677	0.660	0.679
**PNI**	0.722	0.735	0.774
**CRAFITY**	0.676	0.693	0.607
**GPS**	0.626	0.689	0.702
**NLR**	0.656	0.643	0.721
**PLR**	0.607	0.62	0.647

Abbreviations: CABLE: CRP, albumin, bilirubin, lymphocytes, ECOG and EHS; Model$_i$: Cox PH model derived from the imputed training set; ALBI: albumin-bilirubin; PNI: prognostic nutritional index; CRAFITY: CRP and AFP in immunotherapy; EZ-ALBI: Easy-ALBI; mALBI: modified ALBI; GPS: Glasgow prognostic score; NLR: neutrophil-to-lymphocyte ratio; PLR: platelet-to-lymphocyte ratio.

Table S9. Time-dependent AUC of the CABLE score compared to several other prognostic models after 6, 12 and 18 months in the validation set (external validation).

Models	6 months	12 months	18 months
**CABLE score**	0.835	0.713	0.754
**$Model_i$**	0.845	0.714	0.721
**ALBI**	0.839	0.735	0.745
**EZ-ALBI**	0.839	0.743	0.762
**PNI**	0.764	0.732	0.752
**CRAFITY**	0.745	0.634	0.708
**ALBI grades**	0.748	0.675	0.726
**mALBI**	0.816	0.728	0.748
**GPS**	0.804	0.658	0.651
**NLR**	0.542	0.529	0.586
**PLR**	0.433	0.485	0.577

Abbreviations: CABLE: CRP, albumin, bilirubin, lymphocytes, ECOG and EHS; $Model_i$: Cox PH model derived from the imputed training set; ALBI: albumin-bilirubin; PNI: prognostic nutritional index; CRAFITY: CRP and AFP in immunotherapy; EZ-ALBI: Easy-ALBI; mALBI: modified ALBI; GPS: Glasgow prognostic score; NLR: neutrophil-to-lymphocyte ratio; PLR: platelet-to-lymphocyte ratio.

Table S10. Uno's C indices for the validation set with bootstrapped confidence intervals.

Model	6 months	12 months	18 months
CABLE score	0.778 [0.703-0.852]	0.706 [0.627-0.786]	0.706 [0.643-0.769]
Model$_i$	0.79 [0.702-0.879]	0.711 [0.628-0.795]	0.703 [0.629-0.778]
ALBI	0.783 [0.711-0.855]	0.722 [0.642-0.803]	0.719 [0.645-0.793]
EZ-ALBI	0.785 [0.694-0.875]	0.723 [0.654-0.793]	0.72 [0.644-0.796]
ALBI grade	0.714 [0.634-0.795]	0.659 [0.591-0.727]	0.665 [0.617-0.714]
mALBI grade	0.766 [0.695-0.838]	0.706 [0.631-0.781]	0.704 [0.636-0.771]
PNI	0.722 [0.624-0.82]	0.703 [0.634-0.772]	0.702 [0.631-0.774]
CRAFITY	0.719 [0.622-0.817]	0.62 [0.543-0.697]	0.629 [0.559-0.7]
GPS	0.767 [0.689-0.846]	0.675 [0.606-0.743]	0.653 [0.593-0.714]
NLR	0.53 [0.375-0.685]	0.518 [0.413-0.624]	0.532 [0.417-0.647]
PLR	0.565 [0.434-0.696]	0.535 [0.422-0.647]	0.513 [0.413-0.612]

Abbreviations: CABLE: CRP, albumin, bilirubin, lymphocytes, ECOG and EHS; Model$_i$: Cox PH model derived from the imputed training set; ALBI: albumin-bilirubin; PNI: prognostic nutritional index; CRAFITY: CRP and AFP in immunotherapy; EZ-ALBI: Easy-ALBI; mALBI: modified ALBI; GPS: Glasgow prognostic score; NLR: neutrophil-to-lymphocyte ratio; PLR: platelet-to-lymphocyte ratio.

Table S11. Performance of the CABLE score in relevant subgroups of the validation cohort.

Subgroup	n	HR CABLE (95%-CI)
All	157	1.75 [1.397; 2.192]
Child A	114	1.915 [1.274; 2.88]
Child B	43	1.086 [0.75; 1.572]
EHS: no	105	1.641 [1.272; 2.116]
EHS: yes	52	2.173 [1.321; 3.575]
MVI: no	97	1.86 [1.284; 2.694]
MVI: yes	60	1.709 [1.256; 2.326]
ECOG: 0	92	1.89 [1.264; 2.825]
ECOG: 1+2	65	1.677 [1.185; 2.373]
Etiology: non-viral	120	1.669 [1.289; 2.161]
Etiology: viral	31	2.38 [1.364; 4.152]

Abbreviations: HR: hazard ratio; CABLE: CRP, albumin, bilirubin, lymphocytes, ECOG and EHS; CI: confidence interval; EHS: extrahepatic spread; MVI: macrovascular invasion; ECOG: Eastern Cooperative Oncology Group.

Table S12. Uno's C indices for the training set in the subgroup of patients with Child-Pugh A with bootstrapped confidence intervals.

Model	3 months	6 months	12 months	18 months
**CABLE score**	0.769 [0.679-0.859]	0.727 [0.649-0.806]	0.727 [0.67-0.783]	0.718 [0.648-0.787]
**$Model_i$**	0.721 [0.644-0.797]	0.72 [0.647-0.794]	0.704 [0.644-0.763]	0.701 [0.642-0.76]
**ALBI**	0.657 [0.556-0.757]	0.651 [0.569-0.732]	0.641 [0.578-0.705]	0.645 [0.577-0.713]
**EZ-ALBI**	0.637 [0.511-0.763]	0.636 [0.542-0.731]	0.627 [0.558-0.697]	0.629 [0.57-0.688]
**ALBI grade**	0.577 [0.49-0.663]	0.593 [0.507-0.679]	0.584 [0.524-0.643]	0.593 [0.538-0.648]
**mALBI grade**	0.592 [0.485-0.699]	0.611 [0.533-0.689]	0.593 [0.523-0.663]	0.605 [0.541-0.67]
**PNI**	0.66 [0.553-0.768]	0.649 [0.563-0.735]	0.655 [0.579-0.732]	0.649 [0.584-0.714]
**CRAFITY**	0.731 [0.64-0.821]	0.697 [0.626-0.769]	0.672 [0.617-0.727]	0.628 [0.57-0.686]
**GPS**	0.603 [0.513-0.693]	0.576 [0.512-0.639]	0.591 [0.537-0.645]	0.584 [0.532-0.636]
**NLR**	0.713 [0.631-0.795]	0.629 [0.547-0.711]	0.611 [0.536-0.686]	0.596 [0.516-0.677]
**PLR**	0.67 [0.53-0.81]	0.632 [0.557-0.707]	0.613 [0.543-0.683]	0.595 [0.537-0.652]

Abbreviations: CABLE: CRP, albumin, bilirubin, lymphocytes, ECOG and EHS; $Model_i$: Cox PH model derived from the imputed training set; ALBI: albumin-bilirubin; PNI: prognostic nutritional index; CRAFITY: CRP and AFP in immunotherapy; EZ-ALBI: Easy-ALBI; mALBI: modified ALBI; GPS: Glasgow prognostic score; NLR: neutrophil-to-lymphocyte ratio; PLR: platelet-to-lymphocyte ratio.

Table S13. Logistic regression models for the prediction of ORR and DCR in the validation cohort.

Model	AUC (ORR)	AUC (DCR)
CABLE score	0.614 [0.517;0.711]	0.613 [0.509;0.716]
$Model_i$	0.625 [0.529;0.721]	0.592 [0.48;0.704]
ALBI	0.644 [0.545;0.742]	0.636 [0.535;0.737]
EZ-ALBI	0.648 [0.549;0.747]	0.658 [0.559;0.757]
ALBI grade	0.595 [0.508;0.683]	0.627 [0.536;0.717]
mALBI grade	0.599 [0.508;0.69]	0.633 [0.538;0.728]
PNI	0.631 [0.53;0.733]	0.653 [0.558;0.748]
CRAFITY	0.565 [0.47;0.659]	0.598 [0.503;0.694]
GPS	0.606 [0.529;0.682]	0.623 [0.531;0.715]
NLR	0.608 [0.506;0.71]	0.471 [0.366;0.576]
PLR	0.59 [0.491;0.69]	0.514 [0.408;0.62]

Logistic regression models. Abbreviations: ORR: objective response rate; DCR: disease control rate; CABLE: CRP, albumin, bilirubin, lymphocytes, ECOG and EHS; $Model_i$: Cox PH model derived from the imputed training set; ALBI: albumin-bilirubin; PNI: prognostic nutritional index; CRAFITY: CRP and AFP in immunotherapy; EZ-ALBI: Easy-ALBI; mALBI: modified ALBI; GPS: Glasgow prognostic score; NLR: neutrophil-to-lymphocyte ratio; PLR: platelet-to-lymphocyte ratio.

Table S14. Univariable models to evaluate the association of variables with response to a+b (ORR).

Variable	Explained Deviance	Chi² test (p-value)
Age (years)	2.39 %	0.458
Gender (male vs female)	2.24 %	0.9
Etiology		
9 categories[a]	3.14 %	0.8
Nonviral vs viral vs mixed etiology	2.4 %	0.745
MASLD/MASH vs others	2.26 %	0.58
Cirrhosis (yes vs no)	2.53 %	0.353
Pretreatment (yes vs no)	2.87 %	0.164
**ECOG**	**4.11 %**	**0.0249**
BCLC	3.05 %	0.242
Child-Pugh stage	2.57 %	0.21
EHS (yes vs no)	2.17 %	0.254
**MVI (yes vs no)**	**3.39 %**	**0.0107**
AFP, log10 (ng/ml)	4.52 %	0.253
Albumin (g/dl)	5.17 %	0.197
Bilirubin, log10 (mg/dl)	2.45 %	0.35
Creatinine, log10 (mg/dl)	3.21 %	0.386
CRP, log10 (mg/l)	2.4 %	0.0942
INR, log10	2.3 %	0.442
**Platelets, log10 (/nl)**	**7.05 %**	**0.0157**
**Neutrophils (/nl)**	**5.04 %**	**0.0027**
Lymphocytes (/nl)	1.11 %	0.196
Monocytes (/nl)	0.44 %	0.397

[a]unknown/no underlying liver disease, HBV, HCV, alcohol, MASLD/MASH/metabolic, mixed etiology (viral + non-viral), mixed etiology (only non-viral), mixed (only viral), other etiologies. A Chi² test was used for assessing statistical significance.

Supplementary figures

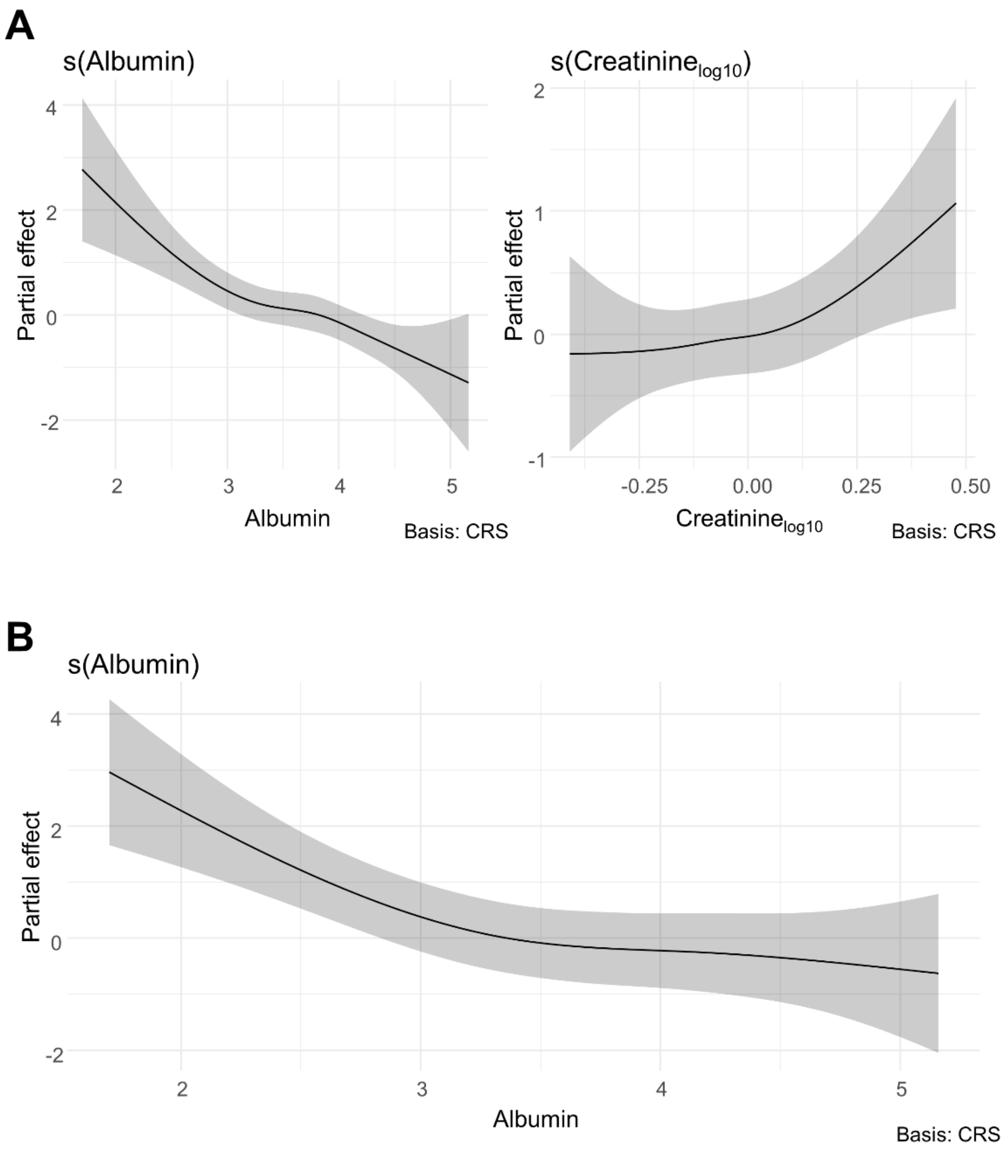

Fig. S1: Smoothing splines of albumin and creatinine$_{log10}$ in the Model$_i$ (A) and of albumin in the CABLE score (B).

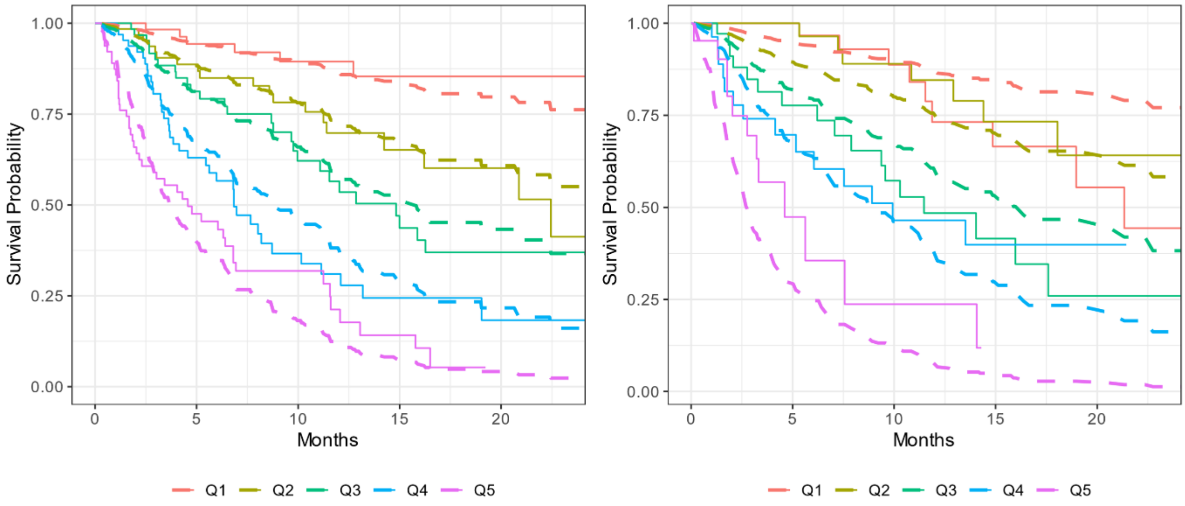

Fig S2. Calibration plots showing the observed (solid line) vs. predicted (dashed line) OS in the training set (left plot) and the test set (right plot) after stratifying patients into Quintiles (Q1-Q5) according to their prognostic score calculated by the CABLE score.

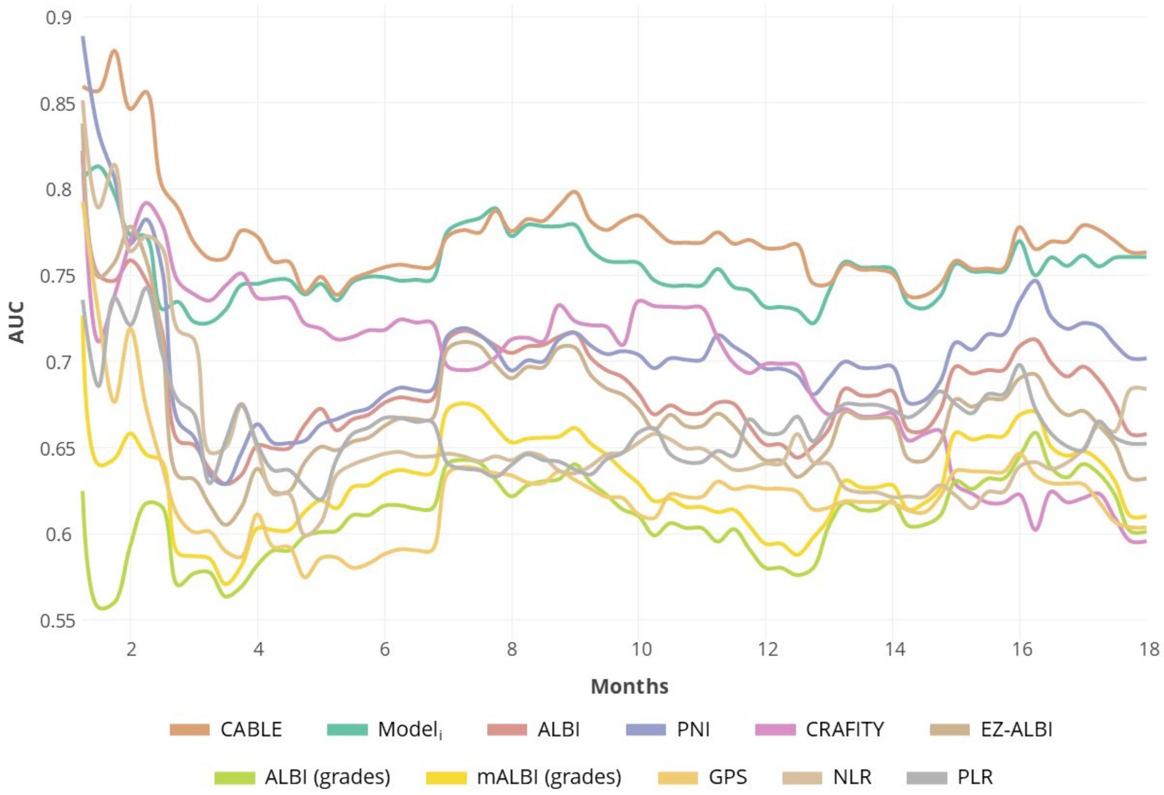

Fig. S3. Time-dependent AUC of the CABLE score and Model$_i$ in comparison with established prediction models in the training set in the subgroup of patients with Child-Pugh A.

Abbreviations: CABLE: **C**RP, **a**lbumin, **b**ilirubin, **l**ymphocytes, **E**COG and **E**HS; Model$_i$: Cox PH model derived from the imputed training set; ALBI: albumin-bilirubin; PNI: prognostic nutritional index; CRAFITY: CRP and AFP in immunotherapy; EZ-ALBI: Easy-ALBI; mALBI: modified ALBI; GPS: Glasgow prognostic score; NLR: neutrophil-to-lymphocyte ratio; PLR: platelet-to-lymphocyte ratio.

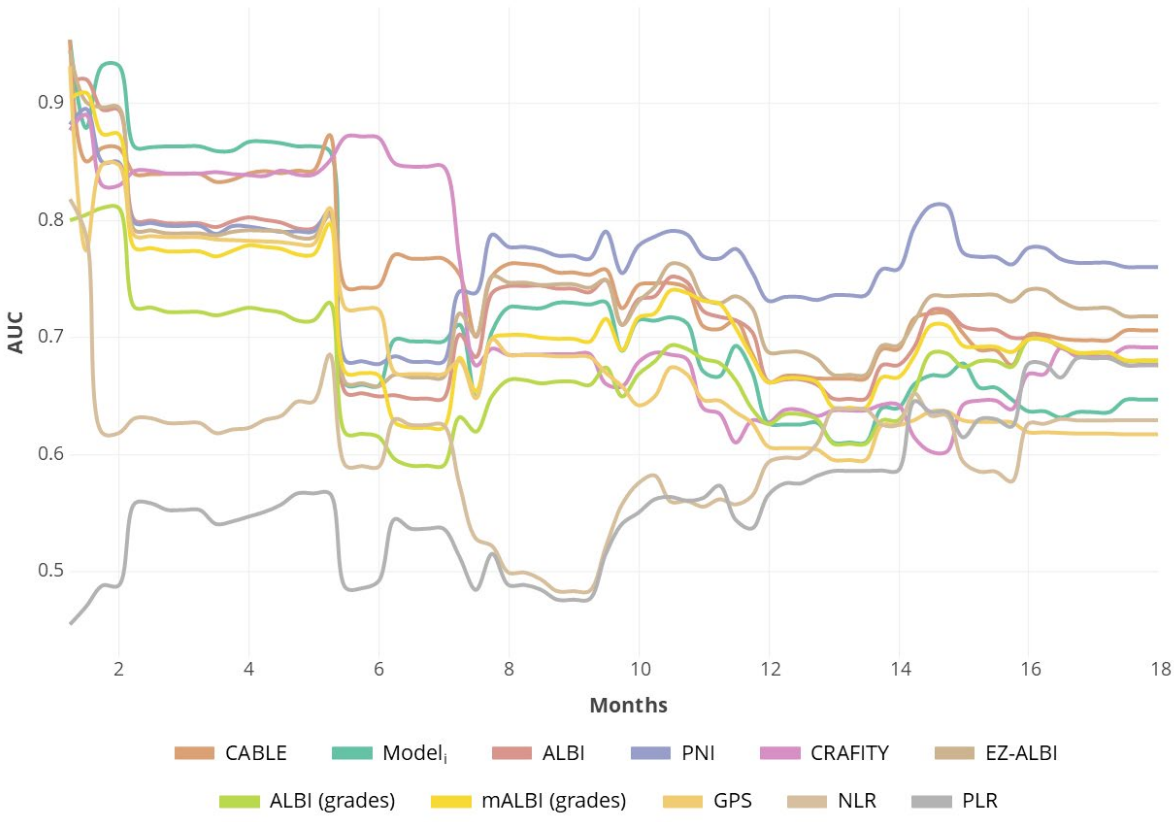

Fig. S4. Time-dependent AUC of the CABLE score and Model$_i$ in comparison with established prediction models in the validation set in the subgroup of patients with Child-Pugh A.

Abbreviations: CABLE: **C**RP, **a**lbumin, **b**ilirubin, **l**ymphocytes, **E**COG and **E**HS; Model$_i$: Cox PH model derived from the imputed training set; ALBI: albumin-bilirubin; PNI: prognostic nutritional index; CRAFITY: CRP and AFP in immunotherapy; EZ-ALBI: Easy-ALBI; mALBI: modified ALBI; GPS: Glasgow prognostic score; NLR: neutrophil-to-lymphocyte ratio; PLR: platelet-to-lymphocyte ratio.

TRIPOD checklist

Section/Topic	Item		Checklist Item	Page
**Title and abstract**				
Title	1	D;V	Identify the study as developing and/or validating a multivariable prediction model, the target population, and the outcome to be predicted.	1 (developme nt/validation not possible due to limitation in title length)
Abstract	2	D;V	Provide a summary of objectives, study design, setting, participants, sample size, predictors, outcome, statistical analysis, results, and conclusions.	8
**Introduction**				
Background and objectives	3a	D;V	Explain the medical context (including whether diagnostic or prognostic) and rationale for developing or validating the multivariable prediction model, including references to existing models.	10-11
	3b	D;V	Specify the objectives, including whether the study describes the development or validation of the model or both.	11
**Methods**				
Source of data	4a	D;V	Describe the study design or source of data (e.g., randomized trial, cohort, or registry data), separately for the development and validation data sets, if applicable.	12
	4b	D;V	Specify the key study dates, including start of accrual; end of accrual; and, if applicable, end of follow-up.	12
Participants	5a	D;V	Specify key elements of the study setting (e.g., primary care, secondary care, general population) including number and location of centres.	12 + Suppl. 2
	5b	D;V	Describe eligibility criteria for participants.	Suppl. 2
	5c	D;V	Give details of treatments received, if relevant.	Suppl. 2
Outcome	6a	D;V	Clearly define the outcome that is predicted by the prediction model, including how and when assessed.	Suppl. 2-3
	6b	D;V	Report any actions to blind assessment of the outcome to be predicted.	n/a
Predictors	7a	D;V	Clearly define all predictors used in developing or validating the multivariable prediction model, including how and when they were measured.	Suppl. 3
	7b	D;V	Report any actions to blind assessment of predictors for the outcome and other predictors.	n/a
Sample size	8	D;V	Explain how the study size was arrived at.	Suppl. 4
Missing data	9	D;V	Describe how missing data were handled (e.g., complete-case analysis, single imputation, multiple imputation) with details of any imputation method.	Suppl. 5-6
Statistical analysis methods	10a	D	Describe how predictors were handled in the analyses.	12-14 + Suppl. 4-6
	10b	D	Specify type of model, all model-building procedures (including any predictor selection), and method for internal validation.	12-14 + Suppl. 4-6
	10c	V	For validation, describe how the predictions were calculated.	12-14 + Suppl. 4
	10d	D;V	Specify all measures used to assess model performance and, if relevant, to compare multiple models.	13-14
	10e	V	Describe any model updating (e.g., recalibration) arising from the validation, if done.	n/a
Risk groups	11	D;V	Provide details on how risk groups were created, if done.	14
Development vs. validation	12	V	For validation, identify any differences from the development data in setting, eligibility criteria, outcome, and predictors.	12 + Suppl. 2
**Results**				
Participants	13a	D;V	Describe the flow of participants through the study, including the number of participants with and without the outcome and, if applicable, a summary of the follow-up time. A diagram may be helpful.	Fig 1
	13b	D;V	Describe the characteristics of the participants (basic demographics, clinical features, available predictors), including the number of participants with missing data for predictors and outcome.	Table 1
	13c	V	For validation, show a comparison with the development data of the distribution of important variables (demographics, predictors and outcome).	Table 1
Model development	14a	D	Specify the number of participants and outcome events in each analysis.	Fig 1
	14b	D	If done, report the unadjusted association between each candidate predictor and outcome.	Table S4
Model specification	15a	D	Present the full prediction model to allow predictions for individuals (i.e., all regression coefficients, and model intercept or baseline survival at a given time point).	Web calculator
	15b	D	Explain how to the use the prediction model.	19-20
Model performance	16	D;V	Report performance measures (with CIs) for the prediction model.	16-19
Model-updating	17	V	If done, report the results from any model updating (i.e., model specification, model performance).	n/a
**Discussion**				

Limitations	18	D;V	Discuss any limitations of the study (such as nonrepresentative sample, few events per predictor, missing data).	23
Interpretation	19a	V	For validation, discuss the results with reference to performance in the development data, and any other validation data.	21-22
	19b	D;V	Give an overall interpretation of the results, considering objectives, limitations, results from similar studies, and other relevant evidence.	24
Implications	20	D;V	Discuss the potential clinical use of the model and implications for future research.	24
**Other information**				
Supplementary information	21	D;V	Provide information about the availability of supplementary resources, such as study protocol, Web calculator, and data sets.	Suppl.
Funding	22	D;V	Give the source of funding and the role of the funders for the present study.	5

References

Author names in bold designate shared co-first authorship

[1] Scheiner B, Pomej K, Kirstein MM et al. Prognosis of patients with hepatocellular carcinoma treated with immunotherapy - development and validation of the CRAFITY score. Journal of Hepatology 2022;76(2):353–63.

[2] **Fulgenzi CAM, Cheon J,** D'Alessio A et al. Reproducible safety and efficacy of atezolizumab plus bevacizumab for HCC in clinical practice: Results of the AB-real study. European journal of cancer (Oxford, England 1990) 2022;175:204–13.

[3] Himmelsbach V, Pinter M, Scheiner B et al. Efficacy and Safety of Atezolizumab and Bevacizumab in the Real-World Treatment of Advanced Hepatocellular Carcinoma: Experience from Four Tertiary Centers. Cancers 2022;14(7):1722.

[4] **Castro T de, Jochheim LS,** Bathon M et al. Atezolizumab and bevacizumab in patients with advanced hepatocellular carcinoma with impaired liver function and prior systemic therapy: a real-world experience. Therapeutic advances in medical oncology 2022;14:17588359221080298.

[5] D’Alessio A, Fulgenzi CAM, Nishida N et al. Preliminary evidence of safety and tolerability of atezolizumab plus bevacizumab in patients with hepatocellular carcinoma and Child-Pugh A and B cirrhosis: a real-world study. Hepatology 2022.

[6] Sinner F, Pinter M, Scheiner B et al. Atezolizumab Plus Bevacizumab in Patients with Advanced and Progressing Hepatocellular Carcinoma: Retrospective Multicenter Experience. Cancers 2022;14(23).

[7] Ben Khaled N, Mörtl B, Beier D et al. Changing treatment landscape associated with improved survival in advanced hepatocellular carcinoma: a nationwide,

population-based study. European journal of cancer (Oxford, England 1990) 2023;192:113248.

[8] Johnson PJ, Berhane S, Kagebayashi C et al. Assessment of liver function in patients with hepatocellular carcinoma: a new evidence-based approach-the ALBI grade. JCO 2015;33(6):550–8.

[9] Onodera T, Goseki N, Kosaki G. Prognostic nutritional index in gastrointestinal surgery of malnourished cancer patients. Nihon Geka Gakkai zasshi 1984;85(9):1001–5.

[10] Nozoe T, Ninomiya M, Maeda T et al. Prognostic nutritional index: a tool to predict the biological aggressiveness of gastric carcinoma. Surgery today 2010;40(5):440–3.

[11] Wu YL, Fulgenzi CAM, D'Alessio A et al. Neutrophil-to-Lymphocyte and Platelet-to-Lymphocyte Ratios as Prognostic Biomarkers in Unresectable Hepatocellular Carcinoma Treated with Atezolizumab plus Bevacizumab. Cancers 2022;14(23).

[12] Kariyama K, Nouso K, Hiraoka A et al. EZ-ALBI Score for Predicting Hepatocellular Carcinoma Prognosis. Liver Cancer 2020;9(6):734–43.

[13] Hiraoka A, Kumada T, Tsuji K et al. Validation of Modified ALBI Grade for More Detailed Assessment of Hepatic Function in Hepatocellular Carcinoma Patients: A Multicenter Analysis. Liver Cancer 2019;8(2):121–9.

[14] Tada T, Kumada T, Hiraoka A et al. New prognostic system based on inflammation and liver function predicts prognosis in patients with advanced unresectable hepatocellular carcinoma treated with atezolizumab plus bevacizumab: A validation study. Cancer medicine 2023;12(6):6980–93.

[15] Åberg F, Luukkonen PK, But A et al. Development and validation of a model to predict incident chronic liver disease in the general population: The CLivD score. Journal of Hepatology 2022;77(2):302–11.
